# Supplementary material for: Haplotype analyses reveal novel insights into tomato history and domestication driven by long-distance migrations and latitudinal adaptations
Source: Hortic Res. 2022 Feb 19;9:uhac030. doi: 10.1093/hr/uhac030 (PMC8976693; doi:10.1093/hr/uhac030)
Supplement: Web_Material_uhac030 [file web_material_uhac030.zip › Supplementary figure 16.pdf]

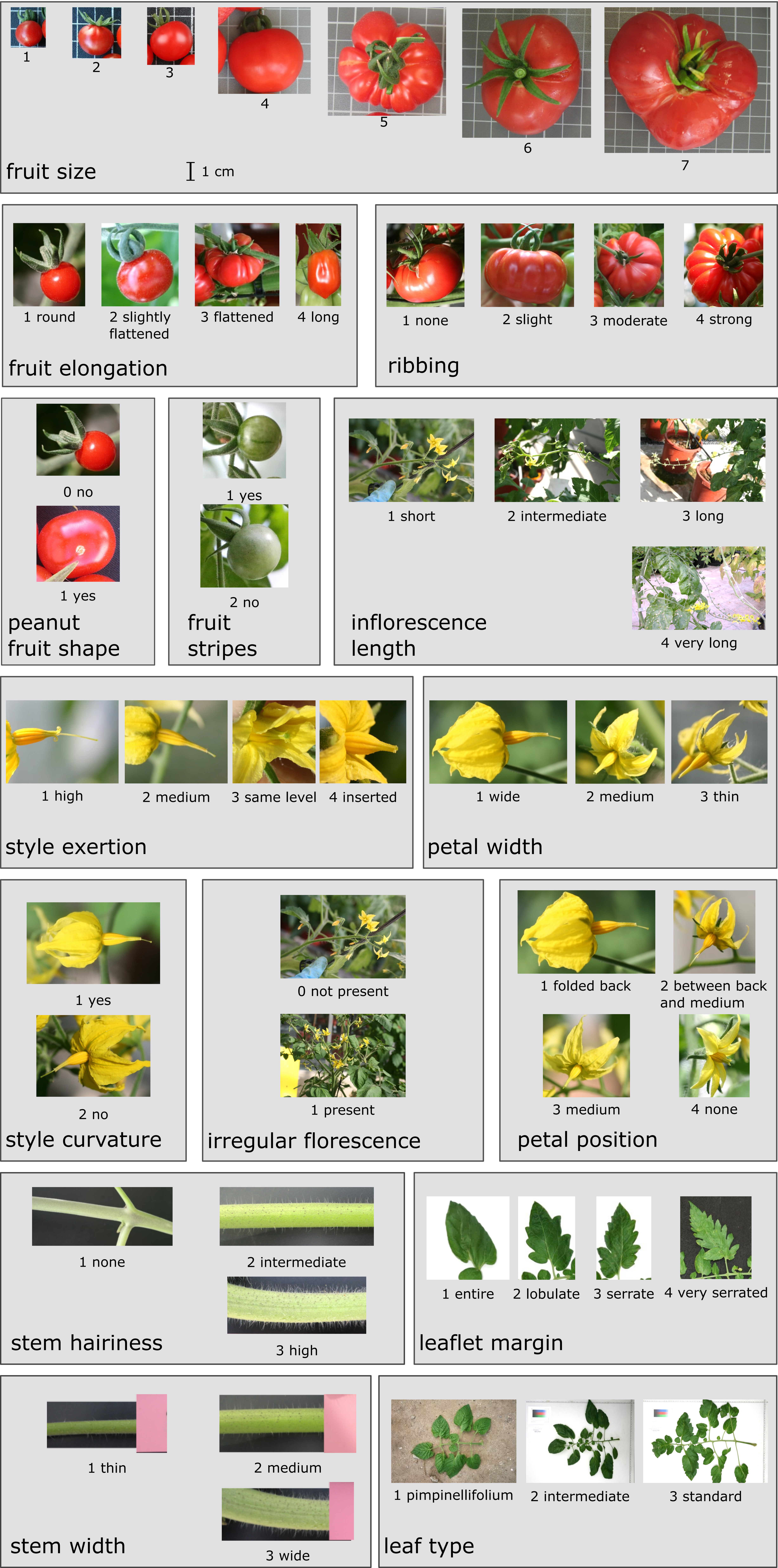

1
2
3
4
5
6
7
fruit size
1 cm
1 round
2 slightly flattened
3 flattened
4 long
fruit elongation
1 none
2 slight
3 moderate
4 strong
ribbing
0 no
1 yes
peanut fruit shape
1 yes
2 no
fruit stripes
1 short
2 intermediate
3 long
4 very long
inflorescence length
1 high
2 medium
3 same level
4 inserted
style exertion
1 wide
2 medium
3 thin
petal width
1 yes
2 no
style curvature
0 not present
1 present
irregular florescence
1 folded back
2 between back and medium
3 medium
4 none
petal position
1 none
2 intermediate
3 high
stem hairiness
1 entire
2 lobulate
3 serrate
4 very serrated
leaflet margin
1 thin
2 medium
3 wide
stem width
1 pimpinellifolium
2 intermediate
3 standard
leaf type
