# Supplementary material for: Haplotype analyses reveal novel insights into tomato history and domestication driven by long-distance migrations and latitudinal adaptations
Source: Hortic Res. 2022 Feb 19;9:uhac030. doi: 10.1093/hr/uhac030 (PMC8976693; doi:10.1093/hr/uhac030)
Supplement: Web_Material_uhac030 [file web_material_uhac030.zip › Supplementary figure 9.pdf]

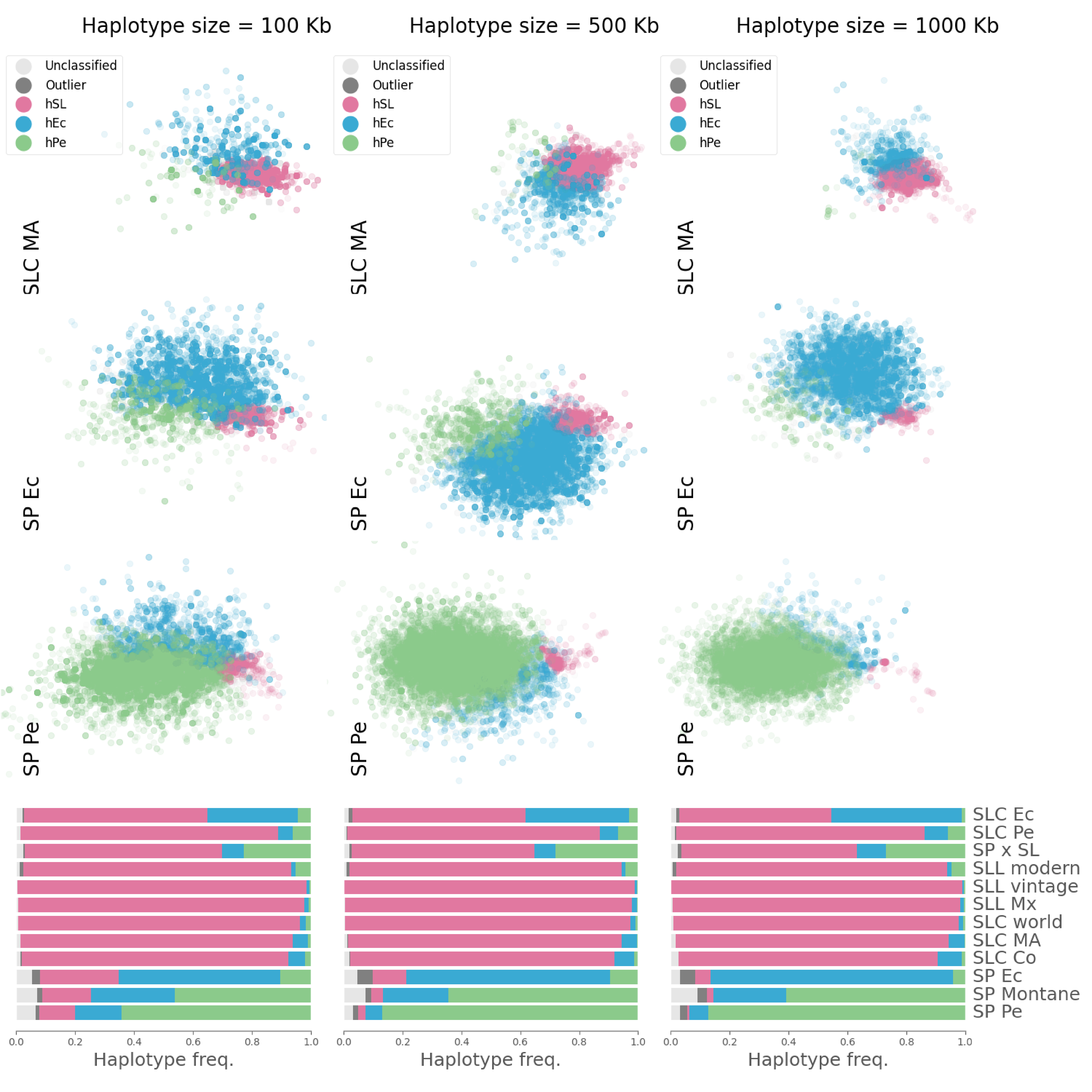

Haplotype size = 100 Kb
Haplotype size = 500 Kb
Haplotype size = 1000 Kb
Unclassified
Outlier
hSL
hEc
hPe
SLC MA
SP Ec
SP Pe
SLC Ec
SLC Pe
SP x SL
SLL modern
SLL vintage
SLL Mx
SLC world
SLC MA
SLC Co
SP Ec
SP Montane
SP Pe
0.0
0.2
0.4
0.6
0.8
1.0
Haplotype freq.
