# Supplementary material for: Haplotype analyses reveal novel insights into tomato history and domestication driven by long-distance migrations and latitudinal adaptations
Source: Hortic Res. 2022 Feb 19;9:uhac030. doi: 10.1093/hr/uhac030 (PMC8976693; doi:10.1093/hr/uhac030)
Supplement: Web_Material_uhac030 [file web_material_uhac030.zip › Supplementary Table 1.pdf]

Taxonomic abreviations

SP	*Solanum pimpinellifolium*
SL	*S. lycopersicum*
SLC	*S. lycopersicum* var. *cerasiforme*
SLL	*S. lycopersicum* var. *lycopersicum*

Populations

SP Pe	Peruvian *S. pimpinellifolium*
SP Ec	Ecuadorian *S. pimpinellifolium*
SLC Ec	Ecuadorian *S. lycopersicum* var. *cerasiforme*
SLC Pe	Peruvian *S. lycopersicum* var. *cerasiforme*
SLC Pe N	Northern Peruvian *S. lycopersicum* var. *cerasiforme*
SLC Pe S	Southern Peruvian *S. lycopersicum* var. *cerasiforme*
SLC Co	Colombian *S. lycopersicum* var. *cerasiforme*
SLC MA	Mesoamerican *S. lycopersicum* var. *cerasiforme*
SLC world	non Mesoamerican or west South American *S. lycopersicum* var. *cerasiforme*
SLL Mx	Mexican *S. lycopersicum* var. *lycopersicum*

Morphological types

SP Pe	typical of the Peruvian *S. pimpinellifolium* accessions
SP intermediate	Intermediate type, with intermediate characteristics between SP Pe and SP Ec
SP Ec	typical of the Ecuadorian *S. pimpinellifolium* accessions
SLC small	small fruited *S. l. cerasiforme* type
SLC big	big fruited *S. l. cerasiforme* type
SLC Ec	*S. l. cerasiforme* type charasteristic of Ecuador
SLL	S. l. lycopersicum type

Haplotypes

hPe	Haplotype kind typically found in Peruvian *S. pimpinellifolium*
hEc	Haplotype kind typically found in Ecuadorian *S. pimpinellifolium* and Ecuadorian *S. l. ceras*
hSL	Haplotype kind typically found in *S. lycopersicum*

Analyses

LD	Linkage Disequilibrium
PCoA	Principal Coordinate Analysis
PCA	Principal Component Analysis
CH	Calinski-Harabasz

iforme
