# Supplementary material for: Haplotype analyses reveal novel insights into tomato history and domestication driven by long-distance migrations and latitudinal adaptations
Source: Hortic Res. 2022 Feb 19;9:uhac030. doi: 10.1093/hr/uhac030 (PMC8976693; doi:10.1093/hr/uhac030)
Supplement: Web_Material_uhac030 [file web_material_uhac030.zip › Supplementary Table 2.pdf]

Accession: Accession ID
Taxon: Ta[illegible] S. pimpine[illegible] S. lycopers[illegible] S. lycopersicum var. lycopersicum (SLL)
Country: Country of origin
Collecting_source: classification of the environment in which the accession was collected. For details
Morphological classification: Classification based only on morphological traits. For details see figure 5
fruit_elor[illegible] slightly fla[illegible] flattened: long:4
fruit_sha[illegible] peanut: 2 slightly fla[illegible] flattened: long:5
fruit_size sp mediun[illegible] slc: 4 bigger: 5 b[illegible] biggest: 7
infloresc[illegible] long: 3 intermedi[illegible] short: 1 irregular: 0
infloresc[illegible] intermedi[illegible] long: 3 very long: 4
leaf_type intermedi[illegible] standard: 3
leaflet_m[illegible] lobulate: 2 serrate: 3 very serrated: 4
peanut_f[illegible] present: 1
petal_po[illegible] between b[illegible] medium: 2[illegible] none: 1
petal_wi[illegible] medium: 2[illegible] thin: 1
presence present:1
ribbing: r[illegible] slight: 2 moderate: strong: 4
stem_hai[illegible] intermedi[illegible] high: 3
stem_wi[illegible] medium: 2[illegible] wide: 3
stripy_fr[illegible] no: 1
style_cur[illegible] no: 1
style_exs[illegible] medium: 2[illegible] same level inserted: 4

Accession	Taxon	Country	Latitude	Longitude	Collecting_	Morpholog	fruit_elong	fruit_shape
BGV00612[illegible]	SP	ECU	-3.95194	-79.4356	natural	sp_interme	1	1
BGV00616[illegible]	SP	ECU	-3.71778	-79.5803		sp_interme	2	3
BGV00618[illegible]	SP	ECU	-3.31722	-79.8033		sp_interme	1	1
BGV00618[illegible]	SP	ECU	-3.33472	-79.6969	disturbed	sp_interme	2	3
BGV00618[illegible]	SP	ECU	-3.31222	-79.6286	disturbed			
BGV00618[illegible]	SP	ECU	-3.31806	-79.5881	disturbed	sp_ec	1	2
BGV00619[illegible]	SP	ECU	-2.59667	-79.4739	disturbed	sp_ec	1	2
BGV00619[illegible]	SP	ECU	-2.46833	-79.4528		sp_interme	1	1
BGV00619[illegible]	SP	ECU	-2.46833	-79.4528		sp_pe	1	2
BGV00620[illegible]	SP	ECU	-3.30806	-79.3481	disturbed	sp_interme	1	1
BGV00621[illegible]	SP	ECU	-3.31556	-79.3539	natural	sp_interme	2	3
BGV00628[illegible]	SP	PER	-5.995	-79.7175			1	1
BGV00632[illegible]	SP	PER	-4.89222	-80.3753	natural_or	sp_pe	1	1
BGV00632[illegible]	SP	PER	-5.15417	-80.1681	natural_or	sp_pe	2	3
BGV00633[illegible]	SP	PER	-5.28722	-79.9581	natural_or	sp_pe	1	2
BGV00633[illegible]	SP	PER	-5.18694	-80.6258	natural	sp_interme	1	1
BGV00633[illegible]	SP	PER	-5.29889	-79.9431	natural	sp_pe	1	1
BGV00633[illegible]	SP	PER	-5.32944	-79.9183	natural	sp_pe	2	3
BGV00633[illegible]	SP	PER	-5.34611	-79.8492	disturbed	sp_pe	1	1
BGV00633[illegible]	SP	PER	-5.43528	-79.7436	natural_or	sp_pe	2	3
BGV00634[illegible]	SP	PER	-5.43306	-79.7422			1	1
BGV00634[illegible]	SP	PER	-5.28167	-80.6842	natural_or	sp_pe	1	1

BGV006343	SP	PER	-5.32222	-80.7136				
BGV006347	SP	PER	-5.27833	-80.1058	natural	sp_pe	1	2
BGV006348	SP	PER	-5.39056	-80.0567	natural	sp_pe	2	3
BGV006349	SP	PER	-5.44	-80.0317	natural_or	sp_pe	2	3
BGV006352	SP	PER	-5.55389	-79.9781	natural	sp_pe	1	1
BGV006353	SP	PER	-5.58917	-79.9703	natural	sp_pe	2	3
BGV006354	SP	PER	-5.59833	-79.9517	natural	sp_pe	1	1
BGV006355	SP	PER	-5.59833	-79.9517	natural_or	sp_pe	2	3
BGV006356	SP	PER	-5.70417	-79.8861	natural_or_disturbed		1	1
BGV006357	SP	PER	-5.81806	-79.8331	natural_or	sp_interme	2	3
BGV006363	SP	PER	-5.995	-79.7175	disturbed		2	3
BGV006368	SP	PER	-5.995	-79.7175	disturbed	sp_interme	1	1
BGV006370	SP	PER	-4.85028	-80.8492	disturbed	sp_pe	1	1
BGV006398	SP	PER	-5.43306	-79.7422	natural_or	sp_pe	2	3
BGV006400	SP	PER	-5.58917	-79.9703	natural	sp_pe	1	2
BGV006413	SP	PER	-5.28333	-80.6872	natural_or_disturbed		1	1
BGV006420	SP	PER	-4.81028	-80.2961	natural	sp_pe	1	1
BGV006452	SP	PER	-5.14417	-80.2789	disturbed	sp_pe	2	3
BGV006454	SP	PER	-5.1475	-80.2703	natural	sp_pe	2	3
BGV006457	SP	PER	-5.17194	-80.1386	disturbed	sp_pe	1	1
BGV006458	SP	PER	-5.26722	-80.1033	natural			
BGV006459	SP	PER	-5.37222	-80.0572	natural			
BGV006461	SP	PER	-5.41861	-80.0347	natural_or_disturbed		2	3
BGV006462	SP	PER	-5.58306	-79.9642	natural_or	sp_pe	1	2
BGV006464	SP	PER	-5.58306	-79.9642	natural_or_disturbed			
BGV006465	SP	PER	-5.58306	-79.9642	disturbed		1	1
BGV006466	SP	PER	-5.59056	-79.9617	natural		1	1
BGV006467	SP	PER	-5.92278	-79.7647	disturbed		1	1
BGV006468	SP	PER	-5.99444	-79.705	natural_or_disturbed			
BGV006469	SP	PER	-5.99444	-79.705	natural_or	sp_pe	1	1
BGV006470	SP	PER	-5.99444	-79.705	natural_or	sp_pe	1	1
BGV006471	SP	PER	-5.99444	-79.705	natural_or	sp_pe	2	3
BGV006472	SP	PER	-5.99444	-79.705	natural_or	sp_interme	1	1
BGV006473	SP	PER	-5.99444	-79.705	natural_or	sp_pe	1	2
BGV006474	SP	PER	-5.99444	-79.705	natural	sp_pe	1	2
BGV006476	SP	PER	-6.01389	-79.6781	natural_or_disturbed			
BGV006477	SP	PER	-6.01389	-79.6781	natural_or_disturbed			
BGV006478	SP	PER	-6.03472	-79.6769	natural_or	sp_interme	1	1
BGV006485	SP	PER	-6.16	-79.7006	natural_or	sp_pe	2	3
BGV006492	SP	PER	-7.19667	-79.4283	disturbed	sp_pe	2	3
BGV006493	SP	PER	-7.19667	-79.4283	disturbed	sp_interme	1	1
BGV006498	SP	PER	-7.30583	-79.4458	disturbed	sp_interme	1	1
BGV006499	SP	PER	-7.30694	-79.4458	natural_or	sp_ec	1	2
BGV006500	SP	PER	-7.30694	-79.4458	natural_or	sp_pe	2	3
BGV006504	SP	PER	-7.31972	-79.3703	disturbed	sp_interme	1	1

BGV00650	SP	PER	-7.22222	-79.2142	disturbed	sp_interme	1	1
BGV00651	SP	PER	-7.22222	-79.2142	natural_or_	sp_interme	1	1
BGV00651	SP	PER	-7.225	-78.975	disturbed	sp_ec	1	1
BGV00651	SP	PER	-7.225	-78.975	disturbed		1	1
BGV00660	SP	PER	-5.10833	-79.9083	disturbed	sp_pe	1	1
BGV00660	SP	PER	-5.025	-79.8917	natural_or_	sp_pe	2	3
BGV00663	SP	PER	-5.50833	-80.5083	disturbed	sp_pe	1	1
BGV00664	SP	PER	-4.91917	-80.6353	disturbed	sp_pe	1	1
BGV00664	SP	PER	-4.88722	-80.4606	natural_or_	sp_pe	1	1
BGV00664	SP	PER	-4.88833	-80.3703	natural	sp_pe	2	3
BGV00665	SP	PER	-4.66917	-79.8911	disturbed	sp_interme	2	3
BGV00666	SP	PER	-4.70694	-79.8481	natural		2	3
BGV00666	SP	PER	-4.67222	-79.8881	natural_or_	sp_pe	2	3
BGV00666	SP	PER	-4.70694	-79.8481	natural		1	1
BGV00669	SP	ECU	-4.14361	-79.8472	natural_or_	sp_interme	1	1
BGV00669	SP	ECU	-4.04361	-79.6886	natural_or_	sp_interme	1	1
BGV00671	SP	ECU	-3.30528	-79.3519	disturbed	sp_interme	1	1
BGV00677	SP	ECU			semi-cultiv	sp_ec	1	2
BGV00706	SP	ECU	0.761667	-78.2517	disturbed	sp_ec	1	2
BGV00706	SP	ECU	0.746667	-78.2436	disturbed	sp_ec	1	2
BGV00706	SP	ECU	0.746667	-78.2436	disturbed	sp_ec	1	2
BGV00706	SP	ECU	0.746667	-78.2436		sp_ec	1	2
BGV00706	SP	ECU	0.746667	-78.2436		sp_ec	1	2
BGV00706	SP	ECU	0.746667	-78.2436		sp_ec		
BGV00707	SP	ECU	0.746667	-78.2436		sp_ec	1	2
BGV00707	SP	ECU	0.746667	-78.2436		sp_ec	1	2
BGV00707	SP	ECU	0.746667	-78.2436		sp_ec	1	2
BGV00707	SP	ECU	0.788611	-78.2922	natural_or_	sp_ec	1	2
BGV00708	SP	ECU	0.788611	-78.2922	natural_or_	sp_ec	1	2
BGV00708	SP	ECU	0.788611	-78.2922	natural_or_	sp_ec	1	2
BGV00708	SP	ECU	0.850556	-78.4253	disturbed	sp_ec	1	2
BGV00709	SP	ECU	0.883056	-78.5	semi-cultiv	sp_ec	1	2
BGV00710	SP	ECU	1.063056	-79.2014	disturbed	sp_ec	1	2
BGV00710	SP	ECU	0.875278	-79.7753	disturbed	sp_ec	1	2
BGV00710	SP	ECU	0.875278	-79.7753	disturbed	slc_small	2	3
BGV00710	SP	ECU	0.875278	-79.7753	disturbed			
BGV00710	SP	ECU	0.875278	-79.7753	disturbed	sp_ec	1	2
BGV00710	SP	ECU	0.989444	-79.5336	disturbed		1	1
BGV00711	SP	ECU	0.995	-79.5611	disturbed	sp_ec	1	2
BGV00711	SP	ECU	0.995	-79.5611	disturbed		1	2
BGV00711	SP	ECU	0.995	-79.5611	disturbed	sp_ec	1	2
BGV00711	SP	ECU	0.993333	-79.5556		sp_ec	1	2
BGV00712	SP	ECU	0.993333	-79.5556		sp_ec	1	2
BGV00712	SP	ECU	0.858333	-79.8756	semi-cultiv	sp_ec	2	3
BGV00713	SP	ECU	-0.24722	-79.1553	semi-cultiv	sp_ec	1	2

BGV00713[illegible]	SP	ECU	-0.24417	-79.2778		sp_ec	1	2
BGV00713[illegible]	SP	ECU	-0.24389	-79.2742		sp_ec	1	2
BGV00714[illegible]	SP	ECU	-0.22194	-79.4889	natural_or_	sp_ec	1	2
BGV00714[illegible]	SP	ECU	-0.21028	-79.4994	disturbed	sp_ec	1	2
BGV00714[illegible]	SP	ECU	-0.18389	-79.5208	disturbed	sp_ec	2	3
BGV00715[illegible]	SP	ECU	-0.17833	-79.5319	disturbed	sp_ec	2	3
BGV00715[illegible]	SP	ECU	-0.12583	-79.5853	natural_or_	sp_ec	3	4
BGV00715[illegible]	SP	ECU	-0.08444	-79.6831	semi-cultiv	sp_ec	1	2
BGV00715[illegible]	SP	ECU	-0.09111	-79.7581	natural_or_	sp_interme	2	3
BGV00715[illegible]	SP	ECU	-0.09111	-79.7581	natural_or_	sp_ec	1	2
BGV00715[illegible]	SP	ECU	-0.09222	-79.7606	disturbed	sp_ec	1	2
BGV00716[illegible]	SP	ECU	0.005278	-79.8833	disturbed	sp_ec	1	2
BGV00716[illegible]	SP	ECU	0.005278	-79.8833	disturbed	sp_ec	1	2
BGV00716[illegible]	SP	ECU	0.035833	-79.9506	disturbed			
BGV00716[illegible]	SP	ECU	0.035833	-79.9506	disturbed	sp_ec	1	2
BGV00718[illegible]	SP	ECU	0.071111	-80.0569	disturbed	sp_ec	1	2
BGV00718[illegible]	SP	ECU	0.012222	-80.0897	disturbed	sp_ec	1	2
BGV00718[illegible]	SP	ECU	0.012222	-80.0897	disturbed	sp_ec	1	2
BGV00718[illegible]	SP	ECU	0.012222	-80.0897	disturbed	sp_ec	1	2
BGV00718[illegible]	SP	ECU	0.012222	-80.0897	disturbed	sp_ec	1	2
BGV00719[illegible]	SP	ECU	-0.04806	-80.1364	disturbed			
BGV00719[illegible]	SP	ECU	-0.12556	-80.2169	natural_or_	sp_ec	1	2
BGV00719[illegible]	SP	ECU	-0.30111	-80.3292	weed_or_s	sp_ec	1	2
BGV00720[illegible]	SP	ECU	-0.32972	-80.3458	disturbed	sp_ec	1	2
BGV00720[illegible]	SP	ECU	-0.42306	-80.4481	natural_or_	sp_ec		
BGV00720[illegible]	SP	ECU	-0.4325	-80.4531	natural_or_	sp_ec	1	2
BGV00720[illegible]	SP	ECU	-0.44944	-80.4511	disturbed			
BGV00720[illegible]	SP	ECU	-0.71333	-80.4167	disturbed	sp_ec	1	1
BGV00721[illegible]	SP	ECU	-0.71333	-80.4167	disturbed	sp_ec	1	1
BGV00721[illegible]	SP	ECU	-0.71333	-80.4167	disturbed	sp_ec	1	2
BGV00721[illegible]	SP	ECU	-0.71333	-80.4167	disturbed	sp_ec	1	2
BGV00721[illegible]	SP	ECU	-0.71333	-80.4167	disturbed	slc_ec	2	3
BGV00721[illegible]	SP	ECU	-0.71333	-80.4167	disturbed	sp_ec	2	3
BGV00722[illegible]	SP	ECU	-0.82889	-80.4944	natural_or_	sp_ec	1	2
BGV00722[illegible]	SP	ECU	-0.86	-80.4694	semi-cultiv	sp_ec	1	2
BGV00722[illegible]	SP	ECU	-0.97444	-80.6383	disturbed			
BGV00723[illegible]	SP	ECU	-0.97444	-80.6383	disturbed	sp_intermediate		
BGV00723[illegible]	SP	ECU	-0.97444	-80.6383	disturbed	sp_interme	2	3
BGV00723[illegible]	SP	ECU	-0.97444	-80.6383	disturbed	sp_ec	1	2
BGV00724[illegible]	SP	ECU	-1.07056	-80.1856	semi-cultivated		1	2
BGV00724[illegible]	SP	ECU	-1.07194	-80.1811	disturbed			
BGV00725[illegible]	SP	ECU	-1.04306	-80.0881		sp_ec	2	3
BGV00725[illegible]	SP	ECU	-1.04083	-80.0864		sp_ec	1	2
BGV00725[illegible]	SP	ECU	-1.04889	-80.0903		sp_ec	1	2
BGV00726[illegible]	SP	ECU	-1.33833	-80.7003	weed_or_semi-cultivated			

BGV00726	SP	ECU	-1.33833	-80.7003		sp_ec	2	3
BGV00726	SP	ECU	-1.58917	-80.8417	disturbed			
BGV00727	SP	ECU	-1.58917	-80.8417		sp_ec	1	2
BGV00727	SP	ECU	-1.58917	-80.8417	disturbed	sp_ec	1	2
BGV00727	SP	ECU	-1.58917	-80.8417	disturbed			
BGV00727	SP	ECU	-1.69806	-80.7803	natural_or_	sp_ec	2	3
BGV00727	SP	ECU	-2.25333	-80.7889	disturbed			
BGV00727	SP	ECU	-2.26861	-80.7553	disturbed	sp_ec	1	1
BGV00728	SP	ECU	-2.415	-80.4069	natural_or_disturbed			
BGV00728	SP	ECU	-2.11667	-79.6	disturbed	sp_ec	1	2
BGV00729	SP	ECU	-2.66667	-79.6167	disturbed			
BGV00730	SP	ECU	-2.66667	-79.6167		slc_small	4	5
BGV00730	SP	ECU	-2.98056	-79.7269		sp_interme	1	1
BGV00730	SP	ECU	-2.98056	-79.7269		sp_interme	1	1
BGV00730	SP	ECU	-2.98056	-79.7269		sp_interme	1	1
BGV00733	SP	ECU	-3.32278	-79.8322	semi-cultiv	sp_pe	1	1
BGV00734	SP	ECU	-3.32806	-79.7889		sp_pe	1	1
BGV00734	SP	ECU	-3.32139	-79.7164		sp_interme	1	1
BGV00735	SP	ECU	-3.32139	-79.7164		sp_interme	2	3
BGV00735	SP	ECU	-3.32389	-79.6711				
BGV00735	SP	ECU	-3.32417	-79.6628	disturbed			
BGV00735	SP	ECU	-3.32417	-79.6628	disturbed	sp_interme	2	3
BGV00735	SP	ECU	-3.32417	-79.6628	disturbed		1	1
BGV00736	SP	ECU	-3.32417	-79.6628		sp_interme	1	1
BGV00736	SP	ECU	-3.32472	-79.6589	natural_or_	sp_interme	1	1
BGV00736	SP	ECU	-3.32833	-79.6081	natural_or_	sp_interme	1	1
BGV00794	SP	PER	-6.74167	-79.6417			1	1
BGV00794	SP	PER	-7.15833	-79.4417			1	1
BGV00794	SP	PER	-7.25833	-79.125		sp_interme	1	1
BGV00794	SP	PER	-7.175	-79.025			2	3
BGV00799	SP	PER	-13.0583	-72.025		slc_small	2	3
BGV00799	SLC	PER	-13.0083	-72.5417			2	3
BGV00799	SP	PER				sp_interme	1	1
BGV00799	SP	PER					1	1
BGV00799	SP	PER					1	1
BGV00799	SP	PER				sp_interme	1	1
BGV00799	SP	PER				sp_interme	1	1
BGV00799	SP	PER					1	1
BGV00800	SP	PER	-13.0083	-72.5417		sp_ec	1	1
BGV00800	SP	PER				slc_small	1	1
BGV00803	SP	ECU	-1.475	-80.1083	disturbed			
BGV00804	SP	ECU				sp_ec	1	1
BGV00929	SP	ECU	-0.63667	-90.3975	disturbed	sp_ec	1	2
BGV00930	SP	ECU	-0.65889	-90.28		sp_ec	2	3
BGV00930	SP	ECU	-0.52778	-90.3225	disturbed	sp_ec	1	2

BGV00931(	SP	ECU	-0.56139	-90.3336		sp_ec	1	2
BGV01371!	SP	PER	-9.93194	-78.2208		sp_pe	1	1
BGV01371	SP	PER	-14.8256	-74.9556	disturbed	sp_interme	1	1
BGV01372(	SP	PER	-14.5803	-74.8872	disturbed	sp_interme	1	1
BGV01372	SP	PER	-14.5644	-74.845	disturbed			
BGV01372	SP	PER	-14.5528	-74.8161	natural_or_	sp_interme	1	1
BGV01379	SP	PER	-17.8892	-70.9797			1	1
BGV01538	SP	PER	-5.88556	-78.1761		sp_interme	1	2
GLP-05	SP	ECU			natural	sp_ec	1	1
GLP-07	SP	ECU				sp_ec	2	3
LA2182	SP	PER	-5.93083	-78.6628	disturbed	sp_interme	1	2
LA2188	SP	PER	-5.99167	-78.1917	disturbed	sp_interme	1	1
PE-73	SP	PER				sp_pe	1	1
PE-74	SP	PER					1	1
PT-218	SP	PER	-7.22083	-79.1808	natural_or_disturbed		1	1
PT-259	SP	PER	-7.21417	-78.7767	natural_or_disturbed		1	1
PT-261	SP	PER	-7.2175	-78.7881	natural_or_disturbed		1	1
PT-31	SP	PER			disturbed	sp_interme	1	1
PT-43	SP	PER			disturbed	sp_interme	1	1
PT-53	SP	PER			disturbed	sp_interme	2	3
PT-60	SP	PER			disturbed	sp_interme	1	1
T-46	SP	PER			disturbed	sp_pe	1	1
BGV00589	SLC	ECU	-4.04167	-78.8917	weed_or_s	slc_small	1	1
BGV00591	SLC	ECU	-3.84167	-78.7583		slc_big	3	4
BGV00622	SLC	ECU	-3.50833	-78.5083		slc_ec	1	2
BGV00622	SLC	ECU	-3.06306	-78.4864		slc_ec	3	4
BGV00623(	SLC	ECU	-3.06306	-78.4864		slc_ec	3	4
BGV00623	SLC	ECU	-2.48139	-78.1619		slc_ec	3	4
BGV00623	SLC	ECU	-2.6125	-78.2225			2	3
BGV00623	SLC	ECU	-4.02694	-78.8828		slc_small	1	2
BGV00623!	SLC	ECU	-3.06306	-78.4864		slc_small	3	4
BGV00675	SLC	ECU	-0.91167	-77.8072	semi-cultivated_or_cu		3	4
BGV00676	SLC	ECU	-0.95722	-77.8158	natural_or_	slc_small	2	3
BGV00676	SLC	ECU	-1.035	-77.7858	natural_or_disturbed		3	4
BGV00677!	SLC	ECU	-1.03611	-77.6911	disturbed	slc_small	1	2
BGV00677	SLC	ECU	-1.03611	-77.6911	semi-cultivated		1	1
BGV00677	SLC	ECU	-1.04667	-77.7953	semi-cultiv	slc_ec	1	2
BGV00679	SLC	ECU	-1.06722	-77.7892	semi-cultivated		2	3
BGV00680(	SLC	ECU	-1.12278	-77.8206	semi-cultiv	slc_ec	2	3
BGV00682!	SLC	ECU	-1.23778	-77.8803	semi-cultiv	sll	3	4
BGV00682	SLC	ECU	-1.31833	-77.8889	semi-cultiv	slc_ec	3	4
BGV00685	SLC	ECU	-1.70028	-77.8425	semi-cultiv	slc_big	3	4
BGV00685	SLC	ECU	-1.72389	-77.8806	semi-cultiv	sp_ec	1	2
BGV00686!	SLC	ECU	-1.80917	-77.8292	semi-cultiv	slc_ec	1	1
BGV00686	SLC	ECU	-1.85056	-77.8225	semi-cultiv	slc_small	2	3

BGV00688	SLC	ECU	-2.30361	-78.1189	semi-cultiv	slc_ec	1	2
BGV00689	SLC	ECU	-2.45056	-78.1697	semi-cultiv	slc_ec	1	2
BGV00689	SLC	ECU	-2.54222	-78.1667	semi-cultiv	slc_ec	3	4
BGV00690	SLC	ECU	-2.54222	-78.1667	semi-cultiv	slc_ec	3	4
BGV00690	SLC	ECU	-2.61528	-78.1983	cultivated	sp_ec	1	2
BGV00690	SLC	ECU			semi-cultiv	slc_ec	1	2
BGV00690	SLC	ECU	-2.62056	-78.1986	semi-cultiv	slc_ec	3	4
BGV00691	SLC	ECU	-2.75167	-78.3047	semi-cultiv	sp_ec	1	2
BGV00692	SLC	ECU	-3.66611	-78.6103	semi-cultivated		3	4
BGV00693	SLC	ECU	-3.67056	-78.6122	semi-cultiv	slc_big	3	4
BGV00693	SLC	ECU	-3.78833	-78.7514	semi-cultiv	slc_big	3	4
BGV00701	SLC	ECU	-1.39833	-78.3839	semi-cultivated		3	4
BGV00701	SLC	ECU	-1.40444	-78.3008	semi-cultivated_or_cu		3	4
BGV00702	SLC	ECU	-2.38889	-78.1697		slc_small	3	4
BGV01263	SLC	ECU	-2.74167	-79.925		slc_ec	2	3
BGV00798	SLC	PER	-9.04167	-74.4917		sll	3	4
BGV00798	SLC	PER	-9.04167	-74.4917		slc_big	3	4
BGV00798	SLC	PER	-13.6417	-72.925		slc_small	3	4
BGV00799	SLC	PER					2	3
BGV00803	SLC	PER			disturbed	slc_small	2	3
BGV00803	SLC	PER			cultivated	slc_small	1	1
BGV00804	SLC	PER			disturbed	slc_small	4	5
BGV00804	SLC	PER			disturbed		3	4
BGV00804	SLC	PER						
BGV00806	SLC	PER	-10.575	-75.4917		slc_big	3	4
BGV00806	SLC	PER_N	-6.50833	-76.3583	market	slc_big	3	4
BGV00806	SLC	PER	-8.125	-79.0417			4	5
BGV00807	SLC	PER			disturbed	sll	3	4
BGV00809	SLC	PER_N	-6.14167	-77.0917	semi-cultiv	slc_big	3	4
BGV00809	SLC	PER_N	-6.19167	-76.8417	disturbed			
BGV00809	SLC	PER_N	-6.35833	-76.6583	semi-cultiv	slc_big	3	4
BGV00810	SLC	PER	-5.90833	-77.7917	cultivated	slc_big	3	4
BGV00810	SLC	PER	-14.2544	-69.225	semi-cultiv	slc_big	3	4
BGV00810	SLC	PER			disturbed	slc_small	2	3
BGV00810	SLC	PER			disturbed	slc_small	1	1
BGV00818	SLC	PER	-9.29167	-79.9917			2	3
BGV01262	SLC	PER				sll	3	4
BGV01264	SLC	PER	-13.5583	-72.625		slc_small	3	4
BGV01315	SLC	PER	-11.9828	-76.7675		slc_big	3	4
BGV01316	SLC	PER_N	-6.225	-76.8583		slc_small	3	4
BGV01316	SLC	PER	-13.6083	-70.4583		sp_ec	1	2
BGV01394	SLC	PER	-13.075	-71.175		sll	3	4
BGV01394	SLC	PER_N	-6.05833	-76.975		slc_big	3	4
L00150	SLC	PER						
LA-2309	SLC	PER_N			disturbed			

BGV01572!	SLC	PER_N	-6.05222	-76.9403	semi-cultiv	slc_big	4	5
BGV01573:	SLC	PER_N	-6.1475	-76.8383	semi-cultiv	slc_small	2	3
BGV01605(	SLC	PER_N	-6.315	-76.7011	cultivated	slc_big	3	4
BGV01572(	SLC	PER_N	-6.52611	-76.3011	semi-cultiv	slc_big	3	4
BGV01605:	SLC	PER_N	-6.42028	-76.5175	semi-cultiv	sll	3	4
BGV01605:	SLC	PER_N	-6.43361	-76.5075	cultivated	slc_big	3	4
BGV01605:	SLC	PER_N	-6.44917	-76.4728	cultivated	slc_big	3	4
BGV01573‹	SLC	PER_N	-6.44917	-76.4728	cultivated	sll	3	4
BGV01605‹	SLC	PER_N	-6.44944	-76.4717	cultivated	slc_big	3	4
BGV01605!	SLC	PER_N	-6.44944	-76.4717	cultivated	slc_big	3	4
BGV01605(	SLC	PER_N	-6.43778	-76.5806	cultivated	slc_big	3	4
BGV01572:	SLC	PER_N	-6.32444	-76.6831	cultivated	slc_big	3	4
BGV00458‹	SLC	COL	20.19167	-98.0583		slc_big	1	1
BGV00790:	SLC	MEX	20.175	-96.0583	natural_or_	slc_small	1	1
BGV00790:	SLC	MEX	21.05833	-98.5083	market	slc_small	1	1
BGV00790:	SLC	MEX	21.14167	-98.425	weed_or_s	slc_small	1	1
BGV00790!	SLC	MEX	21.90833	-99.1583	natural_or_	slc_small	1	2
BGV00791(	SLC	MEX	21.99167	-98.9917	natural_or_	slc_small	1	2
BGV00791:	SLC	MEX	21.25833	-99.125	natural_or_	slc_small	1	2
BGV00791:	SLC	MEX	21.34167	-99.0417	natural_or_	slc_small	2	3
BGV00792(	SLC	MEX	21.20833	-99.5417	semi-cultivated		1	2
BGV00792:	SLC	MEX	24.725	-107.792	natural_or_	slc_small	1	2
BGV00792:	SLC	MEX	25.75833	-108.825	natural_or_	slc_small	4	5
BGV00792:	SLC	MEX	25.70833	-109.025	disturbed	slc_small	2	3
BGV00792!	SLC	MEX	25.975	-109.342	natural_or_	slc_small	2	3
BGV00793(	SLC	MEX	26.05833	-109.375	natural_or_	slc_small	1	1
BGV00793:	SLC	MEX	22.39167	-105.458	natural_or_	slc_small	1	1
BGV00793:	SLC	MEX	21.80833	-105.342	natural_or_	slc_small	1	1
BGV00793‹	SLC	MEX	21.80833	-105.342	natural_or_	slc_small	1	1
BGV00793!	SLC	MEX			natural_or_disturbed			
BGV00805:	SLC	MEX	20.45833	-90.025	disturbed	slc_small	2	3
BGV00805:	SLC	MEX	19.04167	-96.2417	natural	sp_ec	1	2
BGV00806:	SLC	MEX	16.85833	-99.9083		sll	3	4
BGV00806:	SLC	MEX	22.225	-97.8583	market	slc_small	1	2
BGV00807(	SLC	MEX	4.491667	-76.3083		slc_small	1	1
BGV00811(	SLC	COL	10.025	-84.225	semi-cultiv	slc_small	2	3
BGV00821:	SLC	CRI	11.075	-85.6417		slc_small	1	1
BGV00821!	SLC	CRI	13.825	-89.4083		slc_small	3	4
BGV00822:	SLC	SLV	13.925	-87.2083			1	1
BGV00822:	SLC	HND	12.05833	-85.4583		slc_small	2	3
BGV00822‹	SLC	NIC	12.025	-84.6583		sll	3	4
BGV00822!	SLC	NIC				slc_small	1	1
BGV00834!	SLC	SLV				slc_small	2	3
BGV00834:	SLC	HND				slc_small	2	3
BGV00834:	SLC	CRI				slc_small	2	3

BGV008354	SLC	CRI	20.45833	-97.325		slc_big	3	4
BGV012614	SLC	MEX					2	3
BGV012615	SLC	COL			disturbed	slc_small	1	1
BGV012627	SLC	COL	9.908333	-83.6917		slc_small	2	3
BGV013134	SLC	CRI				slc_small	1	2
BGV013175	SLC	COL	25.81444	-108.981	disturbed	slc_small	2	3
BGV015728	SLC	MEX	25.99056	-109.269		slc_small	2	3
BGV015729	SLC	MEX				slc_big	2	3
LA1712	SLC	CRI	20.175	-98.0583	disturbed	slc_small	1	2
MEX-059	SLC	MEX	20.275	-97.9583	market	slc_big	2	3
MEX-062	SLC	MEX	20.175	-96.0583	cultivated	sll	3	4
MEX-068_F	SLC	MEX	20.175	-96.0583	market	sll	3	4
MEX-069	SLC	MEX			market	sll	3	4
MEX-117	SLC	MEX			market			
BGV012620	SLC	COL				slc_small	1	1
PI-129088	SLC	COL				slc_big	3	4
PI-406890	SLC	HND				slc_small	1	1
PI-487625	SLC	CRI					1	2
BGV007854	SLL	MEX	21.29167	-89.2583	cultivated	sll	3	4
BGV007857	SLL	MEX	21.09167	-89.625	cultivated	sll	3	4
BGV007860	SLL	MEX	21.25833	-89.0417	market	sll	3	4
BGV007861	SLL	MEX	21.50833	-89.0083	weed_or_s	sll	4	5
BGV007862	SLL	MEX	21.225	-88.8083	weed_or_s	sll	4	5
BGV007863	SLL	MEX	21.225	-89.8083	weed_or_s	sll	1	1
BGV007864	SLL	MEX	21.225	-89.8083	weed_or_s	sll	3	4
BGV007865	SLL	MEX	21.225	-89.8083	weed_or_s	sll	3	4
BGV007866	SLL	MEX	21.00833	-90.0083		sll	3	4
BGV007867	SLL	MEX	20.875	-89.7417		sll	3	4
BGV007868	SLL	MEX	21.00833	-90.0083		sll	3	4
BGV007869	SLL	MEX	20.50833	-89.5083		sll	3	4
BGV007870	SLL	MEX	20.49167	-89.7083		sll	3	4
BGV007871	SLL	MEX	20.49167	-89.7083		sll	3	4
BGV007872	SLL	MEX	20.30833	-89.425		sll	3	4
BGV007873	SLL	MEX	20.50833	-89.5083		sll	3	4
BGV007875	SLL	MEX	20.80833	-89.4083	weed_or_s	sll	3	4
BGV007876	SLL	MEX	20.69167	-88.225		sll	3	4
BGV007878	SLL	MEX	20.325	-90.0417		sll	3	4
BGV007930	SLL	MEX	20.975	-89.6417		sll	3	4

see supplemental figure 19.
[illegible].

fruit_size	inflorescen	inflorescen	leaf_type	leaflet_ma	peanut_fru	petal_posit	petal_widt	presence_c
1	3	3		1	0			0
3	4	4	2	1	0	4	1	0
1	4	4	1	1	0	4	1	0
1	2	2	1	1	0	4	1	0
								0
1	1	1	1	1	1	4	1	0
1	1	1	1	1	1	4	2	0
1	2	2	1	1	0	4	3	0
1	4	4	1	1	1	4	3	0
1	1	1	1	1	0	3	2	0
1	4	4	2		0	4	3	0
1					0			0
1	4	4	1	2	0	4	3	0
1	4	4	1	1	0	4	3	0
1	4	4	1	1	1	4	3	0
1	1	1	1		0			0
1			1	1	0	4	3	0
1	4	4	1	2	0	4	3	0
1	4	4	1	1	0	4	3	0
1	4	4	1	2	0	4	3	0
1	4	4	1	1	0	4	3	0
1					0	4	3	0

								0
1	4	4	1	2	1	4	3	0
1	4	4	1	1	0	4	3	0
1	4	4	1	2	0	4	3	0
1	4	4	1	2	0	4	3	0
1	4	4	1	1	0	4	3	0
1				1	0	4		0
1	4	4	1	1	0	4	3	0
1					0			0
1			1	2	0			0
2	3	3	2	1	0	3	1	0
1	3	3	1	1	0			0
1	4	4	1	1	0	4	2	0
1	4	4		1	0	4	2	0
2	4	4	2	1	1	4	3	0
1					0			0
1	4	4	1	1	0	4	3	0
1	4	4	1	1	0	4	3	0
1	4	4	1	1	0	4	3	0
1	4	4	1	1	0	4	3	0
								0
								0
1					0			0
1	4	4	1	1	1	4	3	0
								0
1					0			0
1					0			0
1					0			0
			1	1				0
1	4	4	1	1	0	3	3	0
1	4	4	1	1	0	4	3	0
1	4	4	1	1	0	4	3	0
1	4	4			0	4	3	0
1	4	4	1	1	1	4	3	0
1	4	4	1	1	1	4	3	0
								0
								0
1	3	3	2	2	0	4	2	0
1	3	3	2	1	0	4	3	0
1	3	3	1	1	0	4	2	0
1					0			0
1					0	4		0
1	2	2			1			0
1	4	4	1	1	0	4	3	0
1					0	4		0

1			1	1	0	4	1	0
1	3	3		1	0			0
1			1	1	0			0
1					0			0
1	4	4	1	1	0	4	3	0
1	4	4	1	1	0	4	3	0
1	4	4	1	1	0	4	3	0
1				1	0	4	2	0
1	4	4	1	2	0	4	3	0
1	4	4	1	2	0	4	2	0
1	2	2	1	1	0	4	1	0
1	4	4	1	1	0	4	1	0
1	4	4	1	1	0	4	1	0
1					0			0
1	1	1	1	1	0			0
2	3	3			0	4	3	0
1	1	1	1	1	0	3	2	0
2	4	4	1	1	1	3	1	0
3	1	1	2	1	1	4	2	0
3	1	1	2	1	1	3	2	0
3	1	1		1	1			0
3	1	1	2	1	1	3	2	0
3	1	1	2	1	1	2	2	0
3	1	1	2	1			1	0
3	1	1	2	1	1	4	2	0
2	1	1	2	1	1			0
2	1	1	2	1	1			0
2	1	1	2	1	1			0
1	1	1			1			0
2	2	2	1	1	1	4	1	0
2	1	1		1	1			0
1	1	1	1	1	1			0
2	1	1		1	1		1	0
1	3	3	2	1	1	3	2	0
1	1	1	2	2	0	2	1	0
	2	2	2	1		3	2	0
2	1	1	1	1	1	4	1	0
1	2	2	2	1	0	3	1	0
1	2	2	2	2	1	3	2	0
1	2	2	3	2	1	3	1	0
2	2	2	3	2	1	3	1	0
2	1	1	2	1	1	3	1	0
2	1	1	3	1	1	3	1	0
1			1	1	0			0
	2	2	2	1	1	3	1	0

	2	2	2	1	1	3	1	0
	2	2	2	1	1	4	1	0
2	2	2	2	1	1	4	1	0
2	2	2	2	1	1	4	1	0
2	2	2	2	1	0	4	2	0
2	1	1	1	1	0	4	2	0
3	2	2	2	1	0	4	1	0
2	1	1	1	1	1	3	1	0
2	3	3	1	1	0	4	1	0
3	1	1	1	1	1	4	2	0
3	1	1	1	1	1	4	1	0
3	1	1	2	1	1	4	1	0
3	2	2	2	1	1		1	0
								0
3	2	2	2	1	1	4	1	0
3	2	2	2	1	1	4	1	0
3	1	1	2	1	1	4	2	0
3	1	1	2	1	1	3	1	0
2	2	2	1	1	1	3	1	0
3	1	1	2	1	1	3	2	0
								0
3	1	1	2	1	1	3	1	0
	2	2	2	1	1	4	1	0
2	1	1	1	1	1	4	1	0
2	2	2	1	1		4	1	0
3	1	1	2	2	1	4	1	0
								0
2	2	2	2	3	0	4	1	0
2	1	1	2	1	0	4	1	0
3	1	1			1			0
2	1	1			1	3	1	0
3					0			0
3	1	1		1	0	3	1	0
3	1	1	2	3	1	4	1	0
2	1	1	2	1	1	4	3	0
								0
	3	3	1	1		3	1	0
2	2	2		1	0	4	2	0
3	1	1	1	1	1	4	2	0
3	0		1	1	1	4	3	1
								0
3	1	1	1	1	0	4	3	0
3	1	1			1			0
3	1	1	1	1	1	4	1	0
								0

3	2	2		1	0	4	2	0
								0
3	0		2	1	1			1
3	1	1	1	1	1	4	2	0
								0
2	1	1		1	0		1	0
								0
1	2	2	1	1	0	4	1	0
								0
2	3	3	3	1	1	3	1	0
								0
4	2	2	3	1	0	2	1	0
1	3	3	1	1	0	4	1	0
1	3	3	2	1	0	4	1	0
1	4	4	1	1	0	4	1	0
1	4	4	1	1	0	4	3	0
1	4	4	1	1	0	4	3	0
2	4	4	2	1	0	3	1	0
2	4	4	1	1	0			0
							2	0
								0
2	4	4	1	1	0	4	1	0
1	4	4		1	0	4	2	0
2	4	4	1	1	0	4	1	0
1	4	4	2	1	0	4	1	0
1	4	4	1	1	0	3	2	0
1					0			0
1					0			0
1	3	3		1	0	3	2	0
1	1	1	2	2	0			0
1	1	1	2	2	0	3	1	0
3	3	3	3	2	0	3	2	0
1	1	1	1	2	0			0
1	1	1	3	1	0			0
1	1	1			0			0
1	3	3	1	1	0			0
1	3	3	3	2	0	4	2	0
1	1	1			0			0
1	1	1		2	0	4	2	0
1	1	1		2	0	2	1	0
								0
1	1	1	1	1	0	4	1	0
2	1	1	2	1	1	3	1	0
1	1	1	3	1	0	3	1	0
2	1	1	2	1	1	2	2	0

2	1	1	2	1	1	2	2	0
1	4	4	1	1	0	4	3	0
1	4	4	1	1	0	4	1	0
1	3	3	1	1	0	3	2	0
								0
1					0			0
1					0			0
1	3	3	1	1	1	4	1	0
1	2	2	2	1	0	3	2	0
1	1	1	2	1	0	3	2	0
1	1	1			1	4	1	0
2	3	3	1	1	0	4	1	0
1	3	3	2		0	3	2	0
1	1	1	2		0			0
1					0			0
1					0			0
1					0			0
1					0			0
1				1	0			0
1	3	3	1	1	0	4	2	0
1	3	3	1	1	0	4	1	0
2	4	4	1	1	0			0
4	1	1	3	2	0	1	1	0
4	1	1	3	2	0	1	2	0
4	3	3	3	3	1	2	1	0
4	0		2	2	0	3	1	1
4	2	2	3	3	0	3	2	0
4	2	2			0	3	1	0
2				2	0			0
3	1	1	3	2	1	3	2	0
4	1	1	3	2	0	3	1	0
4	1	1	3	3	0	2	2	0
1	1	1	3	2	0	2	1	0
4	1	1	3	3	0	1	1	0
2	1	1	3	3	1	3	1	0
4	2	2	3	3	0	2	1	0
5	1	1	3	3	1	4	1	0
4	1	1	3	3	0	2	1	0
4	2	2	1	2	0	3	1	0
5	1	1	3	3	0	3	2	0
4	1	1	2	2	0	3	2	0
5	2	2	2	2	0	1	1	0
3	1	1		1	1	3	1	0
2	4	4	2	3	0			0
3	2	2	2	2	0	1	2	0

4	3	3	3	2	1	1	1	0
4	3	3	3		1	3	1	0
4	3	3	2	2	0	3	1	0
4	3	3	2	1	0	4	2	0
4	3	3	3	2	1	4	2	0
4	3	3	3	2	1	3	1	0
4	2	2			0	3	2	0
4	2	2	2	1	1	3	1	0
5	0		3	3	0	1	2	1
4	0		3	2	0	1	2	1
5	0		3	2	0	1	1	1
4	0		3	2	0	3	1	1
4	0		3	2	0	4	1	1
4	2	2	2	2	0	2	1	0
4	1	1	3	2	0	4	2	0
5	0		3	3	0	3	1	1
4	1	1	3	4	0	1	1	0
4	1	1	3	2	0	3	2	0
5	3	3	3	1	0		2	0
4	1	1	3	3	0	3	1	0
4	1	1	3	2	0	3	2	0
4	2	2	3	2	0	3	1	0
2	2	2	3	2	0	3	1	0
								0
4	1	1	3	4	0	1	1	0
5	1	1	3	2	0	1	1	0
4	1	1	2	2	0	1	2	0
4	0		3	2	0	3	2	1
4	0		3	3	0	1	1	1
								0
4	0		3	3	0	1	1	1
5	2	2	3	3	0	1	1	0
4	0		3	2	0	2	2	1
3	0		3	3	0	3	2	1
2	1	1	3	2	0	3	2	0
3	0		3	2	0		2	1
5	0		3	2	0	3	1	1
4	1	1	3	2	0	1	1	0
2	0		3	2	0	1	1	1
4	1	1	2	3	0	2	1	0
3	4	4	1	1	1	2	1	0
4	0		3	3	0	3	2	1
4	0		3	4	0	1	1	1
								0
								0

4	2	2	3	3	0	1	1	0
4	2	2	3	4	0	2	1	0
5	2	2	3	3	0	2	1	0
5	1	1	3	3	0	1	1	0
5	0		3	2	0	2	1	1
4	1	1	3	3	0	3	1	0
4	0		3	4	0	1	1	1
5	0		3	4	0	2	1	1
5	2	2	3	2	0	3	1	0
5	1	1	3	3	0	3	1	0
5	1	1	3	2	0	2	1	0
5	1	1	3	2	0	2	1	0
5	0		3	2	0	2	2	1
2	2	2	3	3	0	3	3	0
2	2	2	3	2	0	3	2	0
2	1	1	3	2	0	2	2	0
3	1	1	3	2	1	2		0
3	3	3	3	1	1	2	1	0
3	1	1	3	2	1	3	2	0
2	2	2	3	2	0	3	2	0
4	0		3	2	1	3	2	1
3	1	1	3	2	1	3	1	0
2	1	1	3	2	0	3	2	0
1	2	2	3	2	0	2	1	0
1	2	2	3	3	0	3	1	0
1	2	2	3	3	0	2	2	0
1	1	1	3	2	0	3	2	0
2	1	1	2	2	0	3	1	0
2	0		2	2	0	2	1	1
								0
2	1	1	3	2	0	2	2	0
3	1	1	2	2	1	3	2	0
4	0		3	2	0	2	1	1
3	1	1	3	2	1	2	1	0
2	1	1	3	2	0	2	2	0
2	2	2	3	2	0	2	1	0
3	3	3	3	2	0	2	1	0
2	2	2	3	3	0	2	2	0
3	0		2	2	0			1
2	1	1	3	2	0	2	2	0
5	0		3	2	0	2	2	1
1	2	2	3	4	0	2	1	0
3	2	2	3	2	0	3	1	0
2	2	2	3	2	0	1	1	0
4	3	3	3	3	0	2	2	0

5	1	1	3	3	0		1	0
2	1	1	3	1	0	3	3	0
2	1	1	2	2	0	3	2	0
4	2	2	3	2	0	2	2	0
2	2	2	3	2	1	3	2	0
4	3	3	3	2	0	2	1	0
1	2	2	3	3	0	2	1	0
1	0		3	4	0	2	1	1
3	1	1	3	2	1	2	2	0
4	0		3	2	0	2	1	1
5	0		3	2	0	2	3	1
5	0		3	2	0	2	2	1
5	0		3	2	0	2	1	1
								0
2	3	3	2	3	0	2	1	0
5	0		3	2	0	2	2	1
1	2	2	3	2	0	2	1	0
5	2	2	3	3	1	3	1	0
7	0		3	2	0	2	1	1
7	0		3	3	0	3	1	1
7	0		3	2	0		1	1
6	2	2	3	2	0	1	1	0
6	0		3	2	0	3	1	1
6	0		3	2	0	3	1	1
7	0		3	2	0	2	2	1
7	0		3	2	0	3	2	1
7	0		3	3	0	2	1	1
7	0		3	3	0	1	1	1
7	0		3	3	0	1	1	1
7	0		3	3	0	1	1	1
7	0		3	3	0	4	2	1
7	0		3	3	0	3	2	1
7	0		3	3	0	3	1	1
7	0		3	3	0	3	2	1
6	0		3	3	0	2	1	1
6	0		3	2	0	1	1	1
7	0		3	3	0	3	2	1
7	0		3	2	0	3	2	1

ribbing	stem_hairi	stem_widt	stripy_fruit	style_curva	style_exsertion
1		1			
1	3	1		1	3
1	3	1		1	4
1	3	1		1	2
1	3	1	1	1	2
1	2	1	1	1	2
1	1	1	1	1	4
1	3	1		1	4
1	3	1	2	1	2
1	3	1		1	2
1					
1	1	1		1	
1	1	1	2	1	4
1	1	1	2		4
1	1	1			
1		1	2		4
1	1	1	1		4
1	1	1	1	2	4
1	3	1	1	2	4
1	3	1	2	2	4
1	1	1	2	2	4

1	2	1	2	2	4
1	1	1	1	2	4
1	1	1	2	2	4
1	1	1		2	4
1	1	1		1	4
1		1	2		4
1	3	1	1	1	4
1		1			
1		1			
1		1	1	1	2
1	1	1			
1	1	1	1	1	4
1	1	1		2	3
1	1	1		1	4
1					
1	1	1		1	4
1	2	1		2	2
1	2	1		2	4
1	1	1	2	2	4
1					
1	1	1		2	4
1		1			
1		1			
1		1			
	1				
1	1	1			4
1	1	1	1		3
1	1	1	1	1	3
1	1	1	1	1	
1	1	1		2	4
1	1	1	1		4
1					
1					
1	1	1	1	1	2
1	1	1		1	4
1	1	1		2	4
1		1	2		
1		1	2		
1	1	1	1		
1	1	1		2	4
1		1			

1	3	1	1		3
1	2	1	2		
1	1	1			
1		1			
1	1	1		2	4
1	1	1			4
1	1	1	1	1	4
1	1	1	2		4
1	1	1		1	4
1	1	1	2	1	4
1	1	1		1	4
1	3	1	2	1	4
1	1	1		2	4
1					
1	3	1			
1	3	1		1	4
1	3	1		1	2
1	1	1	1	1	4
1	1	1	1	1	2
1	1	1	1	1	2
1	1	1	1	1	2
1	1	1	1	1	2
1	1	1	1	1	2
1	1	1	1	1	1
1	1	1	1	1	2
1	1	1	1		
1	1	1	1		
1	1	1	1		
1	1	1	1		
1	1	1	1	1	1
1	1	1	1		
1	1	1	1		
1	1	1	1		
1	2	1	1	1	2
1	2	1	1	1	1
	2	1		1	1
1	1	1	1	1	3
1	2	1		1	1
1	2	1		1	3
1	2	1		1	1
1	1	1	1	1	2
1	1	1		1	1
1	1	1	1	1	1
1	1	1			
1	1	1		1	3

1	2	1	1	1	1
1	1	1	1	1	3
1	1	1	1	1	3
1	1	1	1	1	3
1	1	1	1	1	
1	2	1	1	1	3
1	1	1	1	1	3
1	1	1	1	1	3
1	1	1	1	1	3
1	1	1	1	1	3
1	1	1	1	1	3
1	1	1	1	1	2
1	1	1	1	1	
1	1	1	1	1	2
1	1	1	1	1	3
1	1	1	1	1	3
1	1	1	1	1	3
1	1	1	1	1	2
1	1	1	1	1	3
1	1	1	1	1	2
1	1	1	1	1	3
1	1	1	1	1	3
1	1	1	1	1	3
1	1	1	1	1	2
1	1	1	1	1	3
1	1	1	1	1	3
1	1	1			
1	1	1	1	1	1
1	1	1			
1	1	1		1	3
1	1	1		1	2
1	1	1	1	1	4
	2	1		1	2
1	2	1		1	2
1	1	1	1	1	2
1	1	1	1	1	3
1	1	1	1	1	3
1	1	1	1		
1	1	1		1	3

1	1	1		1	3
1	1	1	1		
1	1	1		1	3
1	1	1		1	3
1	1	1	1	1	2
	1				
1	1	1		1	2
1	2	1	1	1	1
1	1	1		1	4
1	2	1		1	4
1	1	1		1	4
1	2	1	1	1	3
1	3	1		1	3
1	1	1		1	2
1	3	1	1		
	3				
1	3	1		1	3
1	3	1		2	3
1	3	1	1	1	3
1	3	1		1	3
1	3	1		1	2
1		1			
1		1			
1	1	1			
1	2	1			
1	2	1		1	2
1	2		1	1	2
1	1	1			
1	3	1			
1	3	1			
1	3	1			
1	1	1		1	3
1	2	1			
1	1	1		1	3
1	1	1		1	2
1	1	1	1	1	3
1	1	1	1	1	3
1	1	1	1	1	3
1	1	1	1	1	3

1	1	1	1	1	3
1	1	1	2	1	3
1	1	1	2	1	1
1	1	1		1	3
1		1	2		
1		1			
1	1	1	1	1	4
1	1	1	1	1	3
1	1	1	1	1	3
1	2	1	2	2	3
1	1	1	2	1	3
1	1	1		2	3
1	3	1			
1		1			
1		1			
1		1			
1		1	2		
1		1	2		
1	3	1		1	2
1	3	1		1	1
1	1	1	2		
1	2	1	1	1	1
4	2		1	1	2
1	1	1	1	1	1
1	1	1	1	1	1
1	1	1	1	1	2
3	2	1	1	1	2
1					
1	2	1	1	1	1
1	2	1	1	1	1
4	1	1	1	1	1
1	2	2	1	1	2
1	1	2	1	1	2
1	2	1	1	1	2
1	1	1	1	1	2
1	1	1	1	1	2
1	1	1	1	1	2
1	1	2	1	1	1
4	1	1	1	1	3
3	1	1	1	1	2
4	2	2	1	1	2
1	1	1	1	1	3
1	1	1	1		
1	2	1		1	2

1	1	1	1	1	1
1	1	1	1	1	1
2	1	1		1	1
1	1	2	1	1	2
1	2	1	1	1	2
1	1	1	1	1	3
2	1	1	1	1	2
1	1	1		1	2
3	3	2	1	1	1
1	2	2	1	1	1
2	2	1	1	1	1
1	2	2	1	1	2
2	2	2	1	1	1
2	2	1		1	1
1	1	1	1	1	1
4	1		1	1	
4	2		1	1	1
1	2	2	1	1	1
1	2	2	1	1	
1	2		1	1	1
1	2	1	1	1	1
1	2	2	1	1	1
1	1	2	1	1	2
3	3	1	1	1	2
3	2	2	1	1	1
1	1	1	1	1	1
3	1	2	1	1	1
2	2	2	1	1	1
1	2	2	1	1	2
2	2	2	1	1	1
1	2	2	1	1	1
1	2	1	1	1	1
1	2	2	1	1	1
1	2	2	1	1	
1	1	2	1	1	2
1	2	1	1	1	1
2	1	2	1	1	1
1	2		1	1	1
1	1	1		1	2
1	1	2	1	1	
2	2	2	1	1	1

1	2	2	1	1	1
1		1	1	1	1
2	2	2	1	1	2
2	2	2	1	1	1
2	2	2	1	1	2
3	2	2	1	1	2
1	2	2	1	1	1
4	2	1	1	1	1
3	2	2	1	1	1
3	2	2	1	1	1
2	2	2	1	1	1
3	2	2	1	1	2
1	2	2	1	1	1
1	3	2	1	1	1
1	2	2	1	1	1
1	2	1	1	1	
1	2	2		1	1
1	2	1		1	1
1	2			1	1
1	2	2		1	1
1	2	2	1	1	1
1	2	1	1	1	1
1	2		1	1	1
1	1	1		1	1
1	1	1	1	1	2
1	2	1		1	1
1	1	1	1	1	1
1	1	1		1	1
1	2	1	1	1	1
1	2	1	1	1	1
1	1	1	1	1	2
2	2	2	1	1	2
1	2	1	1	1	1
1	2	1	1	1	1
1	2	2	1	1	1
1	2	1	1	1	1
1	2	2	1	1	2
1	3	2	1		
1	2	2	1	1	1
2	2	2	1	1	1
1	2		1	1	2
1	2	2		1	1
1	2	2	1	1	1
1	2	2	1	1	1

2	2	2	1	1	
1	3	1	1	1	1
1	2	1	1	1	1
1	2	2	1	1	1
1	2		1	1	1
1	2	2	1	1	1
1	1	1	1	1	1
1	2	2	1	1	2
1	2		1	1	1
1		2	1	1	1
2	2	2	1	1	2
4	2	2	1	1	2
4	3	2	1	1	1
1	2		1	1	1
1	2	2	1	1	1
1	2	1	1	1	2
2	2	2	1	1	1
1	3	3	1	1	1
2	2	3	1	1	2
2	2	3	1	1	
2	2	3	1	1	1
1	2	3	1	1	1
1	2	3	1	1	2
3	3	3	1	1	1
1	2	3	1	1	2
1	2	3	1	1	2
1	2	3	1	1	1
4	2	3	1	1	2
3	2	3	1	1	2
1	2	2	1	1	1
1	2	2	1	1	1
1	2	3	1	1	1
2	2	2	1	1	2
1	2	2	1	1	1
1	2	2	1	1	
3	2	3	1	1	3
2	2		1	1	2
