# Supplementary material for: Haplotype analyses reveal novel insights into tomato history and domestication driven by long-distance migrations and latitudinal adaptations
Source: Hortic Res. 2022 Feb 19;9:uhac030. doi: 10.1093/hr/uhac030 (PMC8976693; doi:10.1093/hr/uhac030)
Supplement: Web_Material_uhac030 [file web_material_uhac030.zip › Supplementary Table 3.pdf]

Accession: Accession ID
Reference: Study of origin in which the sequencing is described
Passport taxon: Taxon stated in the passport data
Country: Country of origin
Mean cov.: Mean sequencing coverage after mapping
Mean MAPQ57 cov.: Mean sequencing coverage after mapping and filtering for a MAPQ equal or gre

Accession	Reference	Passport ta	Country	Latitude	Longitude	Mean cov.	Mean MAP	Genetic cla
ECU1032		S. lycopersi	ECU	-1.06722	-77.7892	6.290206	4.773634	slc_ec
ECU1516B		S. pimpinel	ECU	-1.69806	-80.7803	5.122011	3.651709	sp_ec
ECU570B			PER	-5.32944	-79.9183	5.648901	3.819051	sp_pe
LA0147	Causse 201	S. lycopersi	HND			20.11394	15.9871	sll_vint
LA1420	Causse 201	S. lycopersi	ECU	0.084722	-76.8828	10.12529	7.861149	slc_ec
cervil	Causse 2013					16.45748	12.41594	sp_x_sl
criollo	Causse 2013					9.470092	7.434973	
ferum	Causse 2013					9.833279	7.621824	sll_modern
levovil	Causse 2013					8.131821	6.143277	sll_vint
plovdiv	Causse 2013					10.31229	7.762658	sp_x_sl
stupicke	Causse 2013					10.2872	8.038259	sll_vint
yellow_pea	Strickler 2015					50.34378	38.78124	slc_world
EA00027	The 100 Tomato Genome Sequencing Consortium 2014					38.74391	29.4348	sp_x_sl
EA00157	The 100 To	S. lycopersicum				46.08327	34.92426	sll_vint
EA00325	The 100 To	S. lycopersi	RUS			37.72171	29.14192	sp_x_sl
EA00371	The 100 To	S. lycopersi	AUS			40.03538	30.94481	sll_modern
EA00375	The 100 To	S. lycopersi	RUS			37.37839	26.83694	sp_x_sl
EA00448	The 100 Tomato Genome Sequencing Consortium 2014					38.85034	30.63557	sll_vint
EA00465	The 100 To	S. lycopersi	USA			40.53812	31.95526	sll_vint
EA00526	The 100 To	S. lycopersi	PER	-7.22722	-79.4294	44.36463	32.52323	sp_x_sl
EA00674	The 100 Tomato Genome Sequencing Consortium 2014					46.48766	31.41638	sp_x_sp
EA00676	The 100 To	S. pimpinel	PER	-6.37	-79.79	41.89911	29.29402	sp_x_sp
EA00892	The 100 To	S. lycopersi	GTM			47.41343	37.3008	sll_vint
EA00940	The 100 To	S. lycopersi	USA			45.7389	35.26512	sll_vint
EA00990	The 100 Tomato Genome Sequencing Consortium 2014					42.13117	32.30683	sll_vint
EA01019	The 100 To	S. lycopersi	USA			43.32497	32.08627	sll_vint
EA01037	The 100 To	S. lycopersi	BEL			43.11411	32.02663	sll_vint
EA01049	The 100 To	S. lycopersicum				47.22515	36.1636	sll_vint
EA01088	The 100 Tomato Genome Sequencing Consortium 2014					47.85318	36.2501	sll_vint
EA01155	The 100 To	S. lycopersicum				42.61347	33.05589	sll_vint
EA01640	The 100 To	S. lycopersi	USA			44.49844	34.68393	sll_vint
EA01835	The 100 To	S. lycopersi	ITA			46.85362	35.81915	sll_vint
EA01854	The 100 Tomato Geno		ITA			45.98742	35.35735	sll_vint
EA01965	The 100 Tomato Genome Sequencing Consortium 2014					47.0179	34.56839	
EA02054	The 100 To	S. lycopersi	USA			45.7195	34.34647	sll_modern
EA02617	The 100 To	S. lycopersi	GBR			44.77496	35.52151	sll_vint
EA02655	The 100 To	S. lycopersicum				44.57235	34.09932	sll_modern
EA02724	The 100 To	S. lycopersi	NLD			43.80378	31.73672	sll_vint

EA02960	The 100 To	S. pimpinel	ARG			47.48579	36.31478	sp_x_sl
EA02994	The 100 To	S. pimpinel	CZE			46.68141	30.38199	sp_pe
EA03058	The 100 To	S. lycopersi	GTM			47.45083	37.73987	
EA03107	The 100 To	S. lycopersi	PER			46.69679	32.24584	sp_pe
EA03221	The 100 To	S. lycopersi	ESP			46.95192	36.07702	sll_vint
EA03222	The 100 To	S. lycopersi	CUB			43.46692	34.86563	sll_vint
EA03306	The 100 Tomato Geno		CUB			47.09557	34.09279	sp_x_sl
EA03362	The 100 To	S. lycopersi	DEU			39.53392	30.73469	sll_vint
EA03701	The 100 To	S. lycopersi	RUS			44.6085	35.0417	sll_vint
EA04243	The 100 To	S. lycopersi	CHN			47.3593	37.17736	slc_world
EA04710	The 100 To	S. lycopersi	COL			43.07651	32.50401	sp_x_sl
EA04828	The 100 To	S. lycopersi	CHN			48.19621	37.17475	sll_vint
EA04861	The 100 To	S. lycopersi	TUR			47.54212	35.42477	sll_vint
EA04939	The 100 To	S. lycopersi	ZAF			48.24395	37.54846	slc_world
EA05170	The 100 To	S. lycopersi	GTM			46.31122	35.32024	slc_ma
EA05581	The 100 Tomato Geno		USA			44.52117	32.64406	sll_vint
EA05701	The 100 To	S. lycopersi	MEX			47.61142	36.66521	sll_vint
EA05891	The 100 To	S. lycopersi	PER	-12.8917	-72.75	42.44627	31.79335	sp_x_sl
EA06086	The 100 To	S. lycopersi	CRI	9.85	-83.8833	37.59975	28.70868	sp_x_sl
TR00003	The 100 To	S. lycopersi	JPN			44.34649	32.46502	sll_moderr
TR00018	The 100 To	S. lycopersi	USA			45.02079	34.32511	sll_vint
TR00019	The 100 To	S. lycopersicum				44.94597	34.20911	slc_pe
TR00020	The 100 To	S. lycopersi	USA			47.80439	37.06847	sll_vint
TR00021	The 100 To	S. lycopersi	USA			46.78261	33.57008	sll_vint
TR00022	The 100 To	S. lycopersi	FRA			42.38315	33.44944	sll_vint
TR00023	The 100 To	S. lycopersi	USA			45.33554	35.04977	sll_vint
TR00026	The 100 To	S. lycopersi	USA	21.31278	-156.339	42.09373	30.45461	
TR00027	The 100 To	S. lycopersi	ECU	0.085	-76.9925	45.05977	32.76579	
TR00028	The 100 Tomato Genome Sequencing Consortium 2014					46.15852	30.70891	sp_pe
AlisaCraig	The Tomat	S. lycopersi	USA			40.26259	31.16377	sll_vint
Moneymak	The Tomat	S. lycopersi	USA			41.40572	33.85235	sll_vint
heinz1706g	The Tomat	S. lycopersicum				48.61135	36.39322	sll_vint
TS-1	Zhu 2018	S. lycopersi	USA			5.35271	3.763528	
TS-10	Zhu 2018	S. lycopersi	USA			9.372846	6.288431	
TS-100	Zhu 2018	S. lycopersi	ITA			11.13578	8.716468	sll_vint
TS-103	Zhu 2018	S. lycopersicum				8.995854	6.890433	sll_moderr
TS-104	Zhu 2018	S. lycopersi	ITA			8.613222	6.581211	sll_moderr
TS-105	Zhu 2018	S. lycopersi	CRI			9.834105	7.319971	sp_x_sl
TS-106	Zhu 2018	S. lycopersi	CRI			8.452226	6.608894	slc_ma
TS-109	Zhu 2018	S. lycopersicum				9.903328	7.756924	sp_x_sl
TS-11	Zhu 2018					4.913316	3.357945	
TS-110	Zhu 2018	S. lycopersi	CHN			7.830162	5.583009	sll_vint
TS-111	Zhu 2018	S. lycopersicum				7.992565	6.170995	
TS-112	Zhu 2018	S. lycopersi	RUS			10.38878	8.337111	sll_vint
TS-113	Zhu 2018	S. lycopersicum				8.433366	6.701966	sll_vint

TS-114	Zhu 2018	S. lycopersi	RUS			6.315749	4.945949	
TS-115	Zhu 2018	S. lycopersicum				9.788508	5.881757	
TS-116	Zhu 2018					7.989182	4.66006	
TS-117	Zhu 2018	S. lycopersi	ITA			6.388362	5.038158	sll_vint
TS-118	Zhu 2018					7.24479	4.339768	
TS-12	Zhu 2018					4.976714	3.407496	
TS-120	Zhu 2018	S. lycopersicum				9.859134	7.38937	slc_world
TS-127	Zhu 2018	S. lycopersi	PER	-7.22722	-79.4294	6.385502	4.653474	sp_x_sl
TS-128	Zhu 2018	S. lycopersi	USA			7.972631	6.077719	
TS-129	Zhu 2018	S. lycopersi	PER	-6.03333	-76.9667	6.945948	5.160793	slc_pe
TS-13	Zhu 2018					4.969786	3.352668	
TS-131	Zhu 2018	S. lycopersi	CUB			6.364971	4.858223	slc_world
TS-132	Zhu 2018	S. lycopersi	FRA			7.178491	5.439723	sll_vint
TS-135	Zhu 2018	S. lycopersi	CHL	-18.4167	-70.15	7.697376	5.413877	sll_vint
TS-137	Zhu 2018	S. lycopersi	ITA			10.70103	8.306282	sll_vint
TS-138	Zhu 2018	S. lycopersi	ITA			8.397213	6.472534	sll_modern
TS-139	Zhu 2018	S. lycopersi	USA			9.884786	7.516015	sll_vint
TS-14	Zhu 2018					5.053681	3.247031	
TS-140	Zhu 2018	S. lycopersi	ITA			8.420015	6.119978	sll_modern
TS-141	Zhu 2018	S. lycopersi	ITA			10.96918	8.551359	
TS-142	Zhu 2018	S. lycopersi	ITA			9.740352	7.573471	sll_vint
TS-148	Zhu 2018	S. lycopersi	PER	-13.1167	-72.3667	7.037965	5.103516	sp_x_sl
TS-152	Zhu 2018	S. lycopersi	BRA			6.729772	5.120014	sll_vint
TS-154	Zhu 2018	S. lycopersi	MEX	20.48333	-88.2833	8.180043	6.287216	slc_ma
TS-156	Zhu 2018	S. pimpinel	PER	-5.77583	-78.7831	8.862365	6.404387	sp_montar
TS-157	Zhu 2018					8.389272	6.29426	
TS-158	Zhu 2018	S. lycopersi	PER	-12.8667	-72.7167	11.499	8.70543	sp_x_sl
TS-159	Zhu 2018	S. lycopersi	RUS			9.005093	7.000804	sll_vint
TS-16	Zhu 2018					5.019045	3.253122	
TS-160	Zhu 2018	S. lycopersi	RUS			8.937969	6.984121	
TS-161	Zhu 2018	S. lycopersi	RUS			8.722885	7.039257	sll_vint
TS-162	Zhu 2018	S. lycopersi	AZE			7.635229	6.117253	sll_modern
TS-163	Zhu 2018	S. lycopersi	FRA			5.711919	4.44334	sll_vint
TS-165	Zhu 2018	S. lycopersi	MEX			8.050956	6.387643	slc_ma
TS-167	Zhu 2018					7.049493	4.737987	
TS-168	Zhu 2018	S. lycopersi	ITA			8.303411	6.359712	sll_modern
TS-169	Zhu 2018	S. lycopersi	ITA			8.522533	6.884222	sll_vint
TS-17	Zhu 2018					4.89015	3.087949	
TS-170	Zhu 2018	S. lycopersi	ITA			7.1683	5.669569	sll_vint
TS-172	Zhu 2018	S. lycopersi	CAN			9.033924	7.143593	sll_vint
TS-173	Zhu 2018	S. lycopersi	RUS			8.998921	6.917337	sll_vint
TS-174	Zhu 2018	S. lycopersicum				9.609553	7.442867	sll_vint
TS-175	Zhu 2018	S. lycopersi	RUS			8.360597	6.222841	sll_vint
TS-177	Zhu 2018	S. lycopersicum				7.693119	6.017081	sll_vint
TS-178	Zhu 2018	S. lycopersi	ESP			9.592814	7.615556	sll_vint

TS-18	Zhu 2018					4.989931	3.137829	
TS-180	Zhu 2018	S. lycopersi	RUS			6.905836	5.301753	
TS-181	Zhu 2018	S. lycopersi	MEX	18.46667	-96.6	9.397266	7.497572	
TS-183	Zhu 2018	S. lycopersi	DEU			10.2253	8.373369	sll_vint
TS-184	Zhu 2018	S. lycopersi	PER	-6.48778	-76.36	6.789998	5.320088	slc_pe
TS-186	Zhu 2018	S. lycopersi	USA			7.693018	5.084104	sll_modern
TS-187	Zhu 2018	S. lycopersicum var. cerasiforme				8.15102	6.347534	sp_x_sl
TS-189	Zhu 2018	S. lycopersi	PHL			7.92937	5.9755	sll_vint
TS-19	Zhu 2018					4.882572	3.097154	
TS-190	Zhu 2018	S. lycopersi	ITA			8.51257	6.474379	sll_modern
TS-191	Zhu 2018	S. lycopersi	ITA			7.827609	6.020877	sll_vint
TS-192	Zhu 2018	S. lycopersi	RUS			8.706913	6.929207	sll_vint
TS-193	Zhu 2018	S. lycopersi	ITA			7.360483	5.676662	sll_vint
TS-194	Zhu 2018	S. lycopersicum				7.155431	5.228131	sll_vint
TS-197	Zhu 2018	S. lycopersi	LBN			8.652667	6.750187	sll_vint
TS-198	Zhu 2018	S. lycopersicum				7.239373	5.68374	sll_vint
TS-2	Zhu 2018	S. lycopersi	GBR			4.925917	3.371507	sll_vint
TS-20	Zhu 2018					4.938757	3.144189	
TS-200	Zhu 2018	S. lycopersi	ITA			7.165396	5.479426	
TS-201	Zhu 2018	S. lycopersi	SLV			6.753212	5.4709	sll_vint
TS-203	Zhu 2018	S. lycopersi	ITA			6.599753	5.043987	sll_vint
TS-204	Zhu 2018	S. lycopersi	USA			10.83598	7.545981	sll_modern
TS-205	Zhu 2018	S. lycopersicum var. cerasiforme				9.15766	7.130688	sp_x_sl
TS-209	Zhu 2018					7.222754	5.614057	
TS-21	Zhu 2018					4.870251	2.956541	
TS-210	Zhu 2018	S. lycopersi	USA			11.45949	7.295651	sll_modern
TS-211	Zhu 2018	S. lycopersi	USA			6.86842	5.201086	sll_modern
TS-212	Zhu 2018	S. lycopersi	USA			7.933281	4.778014	sll_modern
TS-213	Zhu 2018	S. lycopersi	ITA			7.962358	6.177148	sll_vint
TS-216	Zhu 2018					6.209352	2.849961	
TS-218	Zhu 2018	S. lycopersi	BRA			5.473458	3.600052	
TS-22	Zhu 2018					4.75765	2.905505	
TS-220	Zhu 2018					10.4429	7.808157	
TS-221	Zhu 2018	S. lycopersi	MEX			6.796862	4.368118	slc_world
TS-224	Zhu 2018	S. lycopersi	ECU	-2.19355	-79.9167	5.765428	3.643784	slc_ec
TS-225	Zhu 2018					9.300754	7.307296	
TS-229	Zhu 2018	S. lycopersi	BRA	-12.75	-38.5667	8.272608	6.236401	slc_ma
TS-23	Zhu 2018					4.814163	2.988605	
TS-231	Zhu 2018	S. lycopersicum var. cerasiforme				9.157851	7.201675	sp_x_sl
TS-234	Zhu 2018	S. lycopersicum				5.907916	4.819303	sll_vint
TS-236	Zhu 2018					5.227218	4.07084	
TS-239	Zhu 2018	S. lycopersi	USA			5.570111	4.458009	sll_modern
TS-240	Zhu 2018	S. lycopersi	CUB			9.091577	6.568071	slc_ma
TS-242	Zhu 2018	S. lycopersi	PER	-13.1583	-74.2239	6.387825	4.856012	slc_pe
TS-243	Zhu 2018	S. lycopersi	MEX			7.468771	6.084005	slc_world

TS-244	Zhu 2018	S. pimpinel	PER	-7.33333	-79.5833	7.102535	4.188431	sp_x_sp
TS-245	Zhu 2018	S. lycopersi	CZE			8.099199	6.562062	sll_modern
TS-246	Zhu 2018	S. lycopersicum				7.536469	6.121135	sll_vint
TS-247	Zhu 2018	S. lycopersi	PER			9.819523	7.878837	slc_pe
TS-248	Zhu 2018	S. lycopersicum				7.993916	6.142465	sp_x_sl
TS-249	Zhu 2018	S. lycopersi	MEX	20.96667	-88.3833	7.076303	5.348257	sll_mx
TS-250	Zhu 2018	S. lycopersi	RUS			9.018047	7.236572	slc_world
TS-251	Zhu 2018	S. lycopersi	ESP			7.530156	6.202733	sll_vint
TS-252	Zhu 2018	S. lycopersi	ITA			7.842792	6.199613	sll_vint
TS-253	Zhu 2018	S. lycopersi	USA			5.70919	4.195373	
TS-254	Zhu 2018	S. lycopersicum				6.013731	4.705283	sp_x_sl
TS-255	Zhu 2018	S. lycopersi	RUS			9.411606	7.334234	sll_vint
TS-256	Zhu 2018	S. lycopersi	PER	-6.22278	-76.8192	9.142168	6.459591	slc_pe
TS-258	Zhu 2018	S. lycopersicum var. cerasiforme				7.349106	4.826711	
TS-259	Zhu 2018	S. lycopersi	ITA			6.091065	4.632588	sll_vint
TS-261	Zhu 2018	S. lycopersi	BRA	-19.45	-44.2333	7.856482	5.376393	sll_vint
TS-262	Zhu 2018	S. pimpinel	ECU	-3.99	-79.36	7.193504	5.24929	sp_x_sl
TS-273	Zhu 2018	S. lycopersi	PER	-13.6417	-72.9083	7.159161	5.396481	slc_pe
TS-280	Zhu 2018	S. lycopersicum var. cerasiforme				8.080026	6.296512	slc_world
TS-282	Zhu 2018	S. lycopersi	UKR			7.407728	5.501251	sll_vint
TS-283	Zhu 2018	S. lycopersicum var. cerasiforme				5.555097	4.226468	sll_vint
TS-284	Zhu 2018	S. lycopersi	BRA			7.846745	6.198424	slc_world
TS-285	Zhu 2018	S. lycopersi	USA			9.122908	7.211979	
TS-287	Zhu 2018	S. lycopersicum				9.076849	6.865499	
TS-293	Zhu 2018	S. lycopersicum				7.461828	6.09486	
TS-294	Zhu 2018					10.69434	8.40328	
TS-296	Zhu 2018	S. lycopersicum				8.373542	5.474301	sp_x_sl
TS-299	Zhu 2018	S. lycopersi	ECU	-3.45444	-78.5553	7.03434	5.459551	
TS-3	Zhu 2018	S. lycopersi	ISR			5.404972	3.720191	sll_modern
TS-302	Zhu 2018	S. lycopersi	BRA			8.809795	6.226372	slc_world
TS-304	Zhu 2018	S. lycopersi	PER	-6.50139	-76.3656	6.866016	5.227905	slc_pe
TS-305	Zhu 2018	S. lycopersicum				6.814291	4.517148	sll_modern
TS-306	Zhu 2018	S. lycopersicum				6.467496	4.342525	sll_modern
TS-307	Zhu 2018	S. lycopersicum				5.687458	4.019418	
TS-308	Zhu 2018	S. lycopersicum				7.684131	5.363988	sll_modern
TS-309	Zhu 2018	S. lycopersicum				6.709021	4.551794	sll_modern
TS-310	Zhu 2018	S. lycopersicum				6.775996	4.731657	sll_modern
TS-311	Zhu 2018	S. lycopersicum				5.949946	4.115846	sll_modern
TS-312	Zhu 2018	S. lycopersicum				6.186579	4.179771	sll_modern
TS-313	Zhu 2018	S. lycopersicum				6.76947	4.768131	
TS-314	Zhu 2018	S. lycopersicum				10.098	6.696716	
TS-315	Zhu 2018	S. lycopersicum				11.0202	7.801391	sll_modern
TS-316	Zhu 2018	S. lycopersicum				12.73711	9.206976	sll_modern
TS-317	Zhu 2018	S. lycopersicum				11.95635	8.567774	sll_modern
TS-318	Zhu 2018	S. lycopersicum				8.79687	6.317111	sll_modern

TS-319	Zhu 2018	S. lycopersicum				7.921523	5.606295	sll_modern
TS-320	Zhu 2018	S. lycopersicum				6.582061	4.762521	sll_modern
TS-321	Zhu 2018	S. lycopersicum				9.994496	7.383045	sll_modern
TS-4	Zhu 2018	S. lycopersicum				5.054769	3.411256	sll_modern
TS-400	Zhu 2018	S. lycopersi	CHN			17.25166	12.59731	sll_modern
TS-409	Zhu 2018	S. lycopersi	GTM			9.577766	6.655489	sp_x_sl
TS-41	Zhu 2018	S. lycopersi	SLV			7.610874	5.806496	slc_ma
TS-410	Zhu 2018	S. pimpinel	CAN			6.933592	4.937567	sp_x_sl
TS-411	Zhu 2018	S. pimpinel	PER	-11.1193	-77.3514	8.156004	5.6956	sp_x_sp
TS-413	Zhu 2018	S. pimpinel	ECU	-2.33093	-79.4046	8.74694	6.025027	sp_ec
TS-414	Zhu 2018	S. pimpinel	PER	-11.9708	-76.7917	6.218775	4.339179	sp_pe
TS-415	Zhu 2018	S. pimpinel	PER	-8.925	-78.5667	9.653065	6.675512	sp_pe
TS-416	Zhu 2018	S. pimpinellifolium				9.588473	6.76783	sp_x_sp
TS-417	Zhu 2018	S. pimpinel	PER	-15.4564	-74.4458	7.521679	5.217308	sp_pe
TS-418	Zhu 2018	S. pimpinel	PER	-7.2	-78.9833	7.198208	4.920813	sp_x_sl
TS-419	Zhu 2018	S. pimpinel	PER	-6.06322	-79.0976	7.637653	5.088978	sp_x_sp
TS-42	Zhu 2018	S. lycopersi	RUS			9.987221	7.677317	
TS-420	Zhu 2018	S. pimpinel	PER	-5.5875	-78.5506	7.091806	4.833637	sp_montar
TS-421	Zhu 2018	S. pimpinel	PER	-5.925	-78.05	7.330935	4.723529	sp_x_sp
TS-422	Zhu 2018	S. pimpinellifolium				6.879513	4.688205	sp_pe
TS-423	Zhu 2018	S. pimpinel	PER	-5.65	-78.5	6.895044	4.853506	sp_x_sl
TS-424	Zhu 2018	S. pimpinel	PER	-8.10565	-79.0746	5.707249	4.012892	sp_pe
TS-425	Zhu 2018	S. pimpinel	PER	-7.39043	-79.5622	7.21986	5.093622	sp_pe
TS-427	Zhu 2018	S. pimpinel	PER	-10.075	-78.1464	5.970895	4.44292	sp_x_sl
TS-428	Zhu 2018					2.665955	1.937699	
TS-429	Zhu 2018	S. pimpinel	PER	-6.72744	-79.7794	8.743559	6.035969	sp_x_sp
TS-43	Zhu 2018	S. lycopersicum				6.882706	5.54446	sll_vint
TS-430	Zhu 2018	S. pimpinel	PER	-12.9561	-72.6701	8.820847	6.193939	sp_x_sl
TS-431	Zhu 2018	S. pimpinel	PER	-12.9561	-72.6701	8.141322	5.981957	sp_x_sl
TS-433	Zhu 2018	S. pimpinel	CAN			5.85086	4.192209	sp_x_sl
TS-434	Zhu 2018	S. pimpinel	PER	-7.71667	-79.1167	7.529347	5.119514	sp_pe
TS-435	Zhu 2018	S. pimpinel	PER	-9.27	-78.47	7.171942	4.844385	sp_pe
TS-436	Zhu 2018	S. lycopersicum var. cerasiforme				6.872005	5.19821	sp_x_sl
TS-437	Zhu 2018	S. pimpinel	PER	-7.33333	-79.5833	5.762464	3.888623	sp_pe
TS-438	Zhu 2018	S. pimpinellifolium				8.293793	5.709376	
TS-439	Zhu 2018	S. pimpinel	PER	-3.8	-80.7	8.167887	5.440818	sp_pe
TS-44	Zhu 2018	S. lycopersi	ITA			9.893562	7.560805	sll_vint
TS-45	Zhu 2018	S. lycopersi	USA			6.185951	4.75333	sll_vint
TS-450	Zhu 2018	S. lycopersicum				24.6526	18.57154	sp_x_sl
TS-46	Zhu 2018	S. lycopersicum				6.54005	5.122254	sll_vint
TS-47	Zhu 2018	S. lycopersi	RUS			7.222044	5.700599	sll_vint
TS-48	Zhu 2018	S. lycopersi	MEX			7.360223	5.54992	sll_vint
TS-5	Zhu 2018	S. lycopersi	EGY			5.427563	3.785895	sll_vint
TS-50	Zhu 2018	S. pimpinel	ECU	-2.73333	-79.9167	7.169173	4.956336	
TS-501	Zhu 2018	S. lycopersicum				7.033654	5.436686	sll_vint

TS-502	Zhu 2018	S. lycopersicum				6.817663	5.116015	sll_vint
TS-503	Zhu 2018	S. lycopersicum				6.371136	4.735666	sll_vint
TS-504	Zhu 2018	S. lycopersicum				10.66902	7.925156	sll_vint
TS-505	Zhu 2018	S. lycopersicum				7.522756	5.750115	sll_vint
TS-506	Zhu 2018	S. lycopersicum				6.951275	5.148934	sll_vint
TS-507	Zhu 2018	S. lycopersicum				6.303779	4.784707	sll_vint
TS-508	Zhu 2018	S. lycopersicum				6.900392	5.081956	sll_vint
TS-509	Zhu 2018	S. lycopersicum				6.546564	4.700388	sp_x_sl
TS-51	Zhu 2018	S. lycopersicum				5.805564	4.499632	sll_vint
TS-510	Zhu 2018	S. lycopersicum				7.074671	5.663359	
TS-511	Zhu 2018	S. lycopersicum				6.737499	5.127205	sll_vint
TS-512	Zhu 2018	S. lycopersicum				6.56992	5.194639	sll_vint
TS-513	Zhu 2018					6.389001	4.593077	
TS-514	Zhu 2018	S. lycopersicum				7.008335	5.427904	sll_vint
TS-515	Zhu 2018	S. lycopersicum				6.973843	5.402138	sll_vint
TS-516	Zhu 2018	S. lycopersicum				5.97511	4.638053	
TS-517	Zhu 2018	S. lycopersicum				7.652798	5.904604	sll_vint
TS-518	Zhu 2018	S. lycopersicum				6.72479	5.3697	sll_vint
TS-519	Zhu 2018	S. lycopersicum				8.765222	6.867691	sll_vint
TS-520	Zhu 2018	S. lycopersicum				7.127148	5.233944	sp_x_sl
TS-521	Zhu 2018	S. lycopersicum				7.704996	6.273299	sll_vint
TS-522	Zhu 2018	S. lycopersicum				6.495406	5.017647	sll_vint
TS-523	Zhu 2018					7.09741	4.911097	
TS-524	Zhu 2018	S. lycopersicum				7.487121	5.516713	sll_vint
TS-525	Zhu 2018	S. lycopersicum				8.008592	6.012327	sll_vint
TS-526	Zhu 2018	S. lycopersicum				6.973902	5.301999	sll_vint
TS-527	Zhu 2018	S. lycopersicum				6.863073	5.467177	
TS-528	Zhu 2018	S. lycopersicum				8.117889	5.918984	sp_x_sl
TS-529	Zhu 2018	S. lycopersicum				6.423268	5.003086	sll_vint
TS-53	Zhu 2018	S. lycopersi	ECU	-3.93528	-79.6461	8.53349	6.126491	slc_ec
TS-530	Zhu 2018	S. lycopersicum				7.352617	5.818297	sll_vint
TS-531	Zhu 2018	S. lycopersicum				7.990392	6.227484	sll_vint
TS-532	Zhu 2018	S. lycopersicum				11.68823	8.886508	
TS-533	Zhu 2018	S. lycopersicum				6.576956	5.012401	sll_vint
TS-534	Zhu 2018	S. lycopersicum				6.779245	5.306065	sll_vint
TS-535	Zhu 2018	S. lycopersicum				8.642871	6.738885	sll_vint
TS-536	Zhu 2018	S. lycopersicum				7.135739	5.468458	slc_ma
TS-537	Zhu 2018	S. lycopersicum				8.405567	6.437083	sp_x_sl
TS-538	Zhu 2018	S. lycopersicum				6.116597	4.543036	sll_vint
TS-539	Zhu 2018	S. lycopersicum				6.559221	5.190319	sll_vint
TS-54	Zhu 2018					5.063334	3.251905	
TS-540	Zhu 2018	S. lycopersicum				8.396022	6.040256	sll_vint
TS-541	Zhu 2018	S. lycopersicum				7.905504	6.032681	slc_world
TS-542	Zhu 2018	S. lycopersicum				5.865561	4.674613	sll_vint
TS-543	Zhu 2018	S. lycopersicum				6.127879	4.713926	sll_vint

TS-544	Zhu 2018	S. lycopersicum				5.745985	4.436129	sll_vint
TS-545	Zhu 2018	S. lycopersicum				6.095784	4.54695	sp_x_sl
TS-546	Zhu 2018	S. lycopersicum				6.365118	5.112181	sll_vint
TS-547	Zhu 2018	S. lycopersicum				5.849709	4.619641	sll_vint
TS-548	Zhu 2018	S. lycopersicum				5.862187	4.551485	
TS-549	Zhu 2018	S. lycopersicum				6.356031	4.991422	sll_vint
TS-55	Zhu 2018	S. lycopersicum				8.50197	6.66522	sll_vint
TS-550	Zhu 2018	S. lycopersicum				5.326117	4.181359	
TS-551	Zhu 2018	S. lycopersicum				6.747709	5.271955	sll_vint
TS-552	Zhu 2018	S. lycopersicum				8.583239	6.572414	sll_vint
TS-553	Zhu 2018	S. lycopersicum				6.07138	4.681281	sll_vint
TS-554	Zhu 2018	S. lycopersicum				7.232751	5.699415	sll_vint
TS-555	Zhu 2018	S. lycopersicum				7.513978	5.869568	sll_vint
TS-556	Zhu 2018	S. lycopersicum				10.02799	8.632885	sll_vint
TS-557	Zhu 2018	S. lycopersicum				6.884922	5.447808	sll_vint
TS-558	Zhu 2018	S. lycopersicum				6.295916	4.812162	sll_vint
TS-559	Zhu 2018	S. lycopersicum				6.404607	5.090295	sll_vint
TS-56	Zhu 2018	S. lycopersi	PER	-13.55	-72.6167	8.262272	6.069217	slc_pe
TS-560	Zhu 2018	S. lycopersicum				8.042788	6.434752	sll_vint
TS-561	Zhu 2018	S. lycopersicum				6.039904	4.851859	sll_vint
TS-562	Zhu 2018	S. lycopersicum				6.667366	5.133454	sll_vint
TS-563	Zhu 2018	S. lycopersicum				7.423343	5.517008	sll_vint
TS-564	Zhu 2018	S. lycopersicum				6.441014	4.917136	sll_vint
TS-565	Zhu 2018	S. lycopersicum				6.172489	4.76499	sll_vint
TS-566	Zhu 2018	S. lycopersicum				6.809517	5.387677	sll_vint
TS-567	Zhu 2018	S. lycopersicum				7.419675	5.629188	sll_vint
TS-568	Zhu 2018	S. lycopersicum				7.051829	5.562176	sll_vint
TS-569	Zhu 2018	S. lycopersicum				7.392251	5.728478	sll_vint
TS-57	Zhu 2018	S. lycopersi	PER	-12.6167	-73.7917	8.741418	6.696516	sp_x_sl
TS-570	Zhu 2018	S. lycopersicum				7.836803	5.963776	sll_vint
TS-571	Zhu 2018	S. lycopersicum				14.96413	11.67783	sll_vint
TS-572	Zhu 2018	S. lycopersicum				6.819501	5.229034	sll_vint
TS-573	Zhu 2018	S. lycopersicum				7.092042	5.316514	sll_vint
TS-574	Zhu 2018	S. lycopersicum				6.731144	5.010229	
TS-575	Zhu 2018	S. lycopersicum				6.737298	5.171408	sll_vint
TS-576	Zhu 2018	S. lycopersicum				7.620718	5.768388	sll_vint
TS-577	Zhu 2018	S. lycopersicum				8.737231	6.447961	sll_modern
TS-578	Zhu 2018	S. lycopersicum				6.405783	4.648014	sll_vint
TS-579	Zhu 2018	S. lycopersicum				6.51176	4.723912	sll_vint
TS-58	Zhu 2018	S. lycopersi	RUS			10.07397	7.874144	sll_vint
TS-580	Zhu 2018	S. lycopersicum				17.96203	12.99707	sll_vint
TS-581	Zhu 2018	S. lycopersicum				7.898636	6.166557	sll_vint
TS-582	Zhu 2018	S. lycopersicum				8.176951	5.964493	sp_x_sl
TS-583	Zhu 2018	S. lycopersicum				6.559866	4.986324	sll_vint
TS-584	Zhu 2018	S. lycopersicum				7.68474	5.823404	sll_vint

TS-585	Zhu 2018	S. lycopersicum		8.083752	6.036129	sp_x_sl
TS-586	Zhu 2018	S. lycopersicum		6.022443	4.565038	sp_x_sl
TS-587	Zhu 2018	S. lycopersicum		6.572929	5.03907	sll_vint
TS-588	Zhu 2018	S. lycopersicum		7.98752	6.16881	sll_vint
TS-589	Zhu 2018	S. lycopersicum		6.611322	4.996611	sll_vint
TS-59	Zhu 2018	S. lycopersi	BGR	8.234412	6.151523	sll_modern
TS-590	Zhu 2018	S. lycopersicum		6.954864	5.117046	sp_x_sl
TS-591	Zhu 2018	S. lycopersicum		6.975685	5.458043	sll_vint
TS-592	Zhu 2018	S. lycopersicum		6.586987	4.710351	sll_vint
TS-593	Zhu 2018	S. lycopersicum		7.704979	5.748296	sll_vint
TS-594	Zhu 2018	S. lycopersicum		9.124383	6.500457	slc_ma
TS-595	Zhu 2018	S. lycopersicum		10.5685	8.367329	sll_vint
TS-596	Zhu 2018	S. lycopersicum		6.813818	5.072281	sll_vint
TS-597	Zhu 2018	S. lycopersicum		9.663934	7.190701	sll_vint
TS-598	Zhu 2018	S. lycopersicum		6.914264	5.272339	sp_x_sl
TS-599	Zhu 2018	S. lycopersicum		6.087358	4.883879	
TS-6	Zhu 2018	S. lycopersi	ITA	4.330686	3.104111	sll_vint
TS-60	Zhu 2018	S. lycopersi	USA	6.769568	5.153494	sll_vint
TS-600	Zhu 2018	S. lycopersicum		5.841812	4.373821	sll_vint
TS-601	Zhu 2018	S. lycopersicum		6.581522	4.479742	sp_x_sl
TS-602	Zhu 2018	S. lycopersicum		6.70975	4.897917	sll_modern
TS-603	Zhu 2018	S. lycopersicum		6.111565	4.70794	sll_vint
TS-604	Zhu 2018	S. lycopersicum		9.282464	7.142101	sll_vint
TS-605	Zhu 2018	S. lycopersicum		8.173895	6.31902	sll_vint
TS-606	Zhu 2018	S. lycopersicum		6.676845	5.019054	sll_vint
TS-607	Zhu 2018	S. lycopersicum		6.650357	5.024223	sll_vint
TS-608	Zhu 2018	S. lycopersicum		7.756409	5.539113	sll_vint
TS-609	Zhu 2018	S. lycopersicum		6.161294	4.615219	sll_vint
TS-610	Zhu 2018	S. lycopersicum		12.91345	9.882407	sll_vint
TS-611	Zhu 2018	S. lycopersicum		6.656125	5.060136	sll_vint
TS-612	Zhu 2018	S. lycopersicum		5.808189	4.367797	sll_vint
TS-613	Zhu 2018	S. lycopersicum		6.484748	4.734886	sll_vint
TS-614	Zhu 2018	S. lycopersicum		5.88537	3.965028	sp_x_sl
TS-615	Zhu 2018	S. lycopersicum		7.580661	5.519065	sll_vint
TS-616	Zhu 2018	S. lycopersicum		7.281657	5.747414	sll_vint
TS-617	Zhu 2018	S. lycopersicum		6.661686	5.020791	sll_modern
TS-618	Zhu 2018	S. lycopersicum		7.339497	5.348464	sll_vint
TS-619	Zhu 2018	S. lycopersicum		6.425899	4.68787	sll_vint
TS-620	Zhu 2018	S. lycopersicum		8.332196	6.161688	sp_x_sl
TS-621	Zhu 2018	S. lycopersicum		7.581015	5.837662	sll_vint
TS-622	Zhu 2018	S. lycopersicum		7.732103	5.788794	sll_modern
TS-623	Zhu 2018	S. lycopersicum		6.570514	4.630968	sp_x_sl
TS-624	Zhu 2018	S. lycopersicum		7.10933	5.26462	sll_vint
TS-625	Zhu 2018	S. lycopersicum		13.99053	10.93841	sll_vint
TS-626	Zhu 2018	S. lycopersicum		6.369365	4.629312	sp_x_sl

TS-627	Zhu 2018	S. lycopersicum				6.725114	5.042659	sll_vint
TS-628	Zhu 2018	S. lycopersicum				8.423688	6.16326	sp_x_sl
TS-629	Zhu 2018	S. lycopersicum				5.832567	4.475621	slc_world
TS-63	Zhu 2018	S. lycopersicum				8.304761	6.323868	slc_world
TS-630	Zhu 2018	S. lycopersicum				7.071706	5.357015	sll_modern
TS-631	Zhu 2018	S. lycopersicum				6.332989	4.737107	sll_modern
TS-632	Zhu 2018	S. lycopersicum				7.028867	5.180642	sll_modern
TS-633	Zhu 2018	S. lycopersicum				6.531192	4.700799	sll_modern
TS-634	Zhu 2018	S. lycopersicum				6.839644	5.165351	sll_modern
TS-635	Zhu 2018	S. lycopersicum				7.118516	5.459641	sll_modern
TS-636	Zhu 2018	S. lycopersicum				6.57628	4.928114	sll_modern
TS-637	Zhu 2018	S. lycopersicum				7.062955	5.41399	sll_modern
TS-638	Zhu 2018	S. lycopersicum				6.884948	5.455268	sll_modern
TS-639	Zhu 2018	S. lycopersicum				6.834546	5.226755	
TS-64	Zhu 2018	S. lycopersi	RUS			6.453487	4.657469	
TS-640	Zhu 2018	S. lycopersicum				16.3802	11.57779	sll_modern
TS-641	Zhu 2018	S. lycopersicum				5.550908	4.720962	sll_vint
TS-642	Zhu 2018	S. lycopersicum				6.437733	5.218464	sll_vint
TS-643	Zhu 2018					5.911036	5.016177	
TS-644	Zhu 2018	S. lycopersicum				5.485126	4.695322	sll_vint
TS-645	Zhu 2018	S. lycopersicum				5.731802	4.694364	sll_vint
TS-646	Zhu 2018	S. lycopersicum				5.548615	4.735045	sll_vint
TS-647	Zhu 2018	S. lycopersicum				7.760801	6.258171	sp_x_sl
TS-648	Zhu 2018	S. lycopersicum				7.958357	6.617152	sll_vint
TS-649	Zhu 2018	S. lycopersicum				5.595601	4.676599	sll_vint
TS-65	Zhu 2018	S. lycopersi	MYS			6.205834	4.376738	slc_world
TS-650	Zhu 2018	S. lycopersicum				5.842802	4.745598	sll_vint
TS-651	Zhu 2018	S. lycopersicum				5.10718	4.140444	sp_x_sl
TS-652	Zhu 2018	S. lycopersicum				6.52665	5.151253	sp_x_sl
TS-653	Zhu 2018	S. lycopersicum				4.259311	3.428679	sll_modern
TS-654	Zhu 2018	S. lycopersicum				5.928979	4.753492	sp_x_sl
TS-655	Zhu 2018	S. lycopersicum				5.943358	4.924777	sll_vint
TS-656	Zhu 2018	S. lycopersicum				7.211696	5.80068	sll_modern
TS-657	Zhu 2018	S. lycopersicum				6.121591	4.98054	sp_x_sl
TS-658	Zhu 2018	S. lycopersicum				6.519747	5.142491	sp_x_sl
TS-659	Zhu 2018	S. lycopersicum				6.691528	5.432543	slc_world
TS-66	Zhu 2018	S. lycopersi	PER	-11.1167	-75.35	8.895402	6.47898	slc_pe
TS-67	Zhu 2018	S. lycopersi	SLV			8.569515	6.752668	sll_vint
TS-69	Zhu 2018	S. lycopersi	MEX			6.984224	5.196223	slc_ma
TS-7	Zhu 2018	S. lycopersi	USA			5.020732	3.439996	sp_x_sl
TS-71	Zhu 2018	S. lycopersicum var. cerasiforme				8.952108	6.814501	sp_x_sl
TS-72	Zhu 2018	S. lycopersi	ITA			8.390036	6.708488	sll_vint
TS-73	Zhu 2018	S. lycopersi	ITA			8.527866	6.295076	sll_vint
TS-74	Zhu 2018	S. lycopersi	PER			6.072316	4.637943	slc_pe
TS-75	Zhu 2018	S. lycopersicum var. cerasiforme				8.932221	6.954268	slc_world

TS-76	Zhu 2018	S. lycopersicum				8.265349	6.444416	sll_vint
TS-77	Zhu 2018	S. pimpinel	ECU	0.866667	-79.85	7.590679	5.136776	sp_pe
TS-78	Zhu 2018	S. lycopersi	RUS			9.017863	6.802695	sll_modern
TS-79	Zhu 2018					9.082742	5.443806	
TS-8	Zhu 2018	S. lycopersi	USA			5.122802	3.627238	sll_modern
TS-80	Zhu 2018					8.390279	5.462183	
TS-81	Zhu 2018	S. lycopersi	RUS			7.292213	5.875757	sll_vint
TS-83	Zhu 2018	S. lycopersi	COG			10.87257	8.407358	slc_world
TS-84	Zhu 2018	S. lycopersicum				10.20767	7.860283	slc_pe
TS-85	Zhu 2018	S. lycopersicum				9.755589	7.464979	sll_modern
TS-86	Zhu 2018	S. lycopersi	ITA			8.033897	5.424745	sll_vint
TS-88	Zhu 2018	S. lycopersi	ITA			9.005188	6.873619	sll_vint
TS-89	Zhu 2018	S. lycopersicum				8.826231	6.773963	
TS-9	Zhu 2018	S. lycopersi	GBR			5.019406	3.525169	sll_vint
TS-90	Zhu 2018	S. lycopersi	RUS			11.35934	8.587551	
TS-93	Zhu 2018	S. lycopersicum				7.684828	6.07699	sll_vint
TS-94	Zhu 2018					10.64015	5.789457	
TS-95	Zhu 2018	S. lycopersicum				8.724361	6.758391	sll_vint
TS-99	Zhu 2018	S. lycopersicum var. cerasiforme				10.06342	7.795174	sll_vint
BGV00458	varitome	S. lycopersi	COL			17.29336	13.85261	slc_co
BGV00589	varitome	S. lycopersi	ECU	-4.03028	-78.8992	11.54598	8.693103	slc_world
BGV00591	varitome	S. lycopersi	ECU	-3.82778	-78.7594	6.225455	4.590338	slc_ec
BGV00614	varitome	S. lycopersi	ECU	-3.87082	-79.6468	16.38623	12.10758	
BGV00617	varitome	S. lycopersi	ECU	-3.475	-80.025	15.10564	11.58722	slc_ec
BGV00620	varitome	S. pimpinel	ECU	-3.85833	-79.6583	15.4888	11.42358	sp_ec
BGV00622	varitome	S. lycopersi	ECU	-3.40667	-78.5728	13.52209	10.05912	slc_ec
BGV00622	varitome	S. lycopersi	ECU	-3.07583	-78.4789	14.46318	11.32839	slc_ec
BGV00623	varitome	S. lycopersi	ECU	-3.06667	-78.4667	18.48802	13.97006	slc_ec
BGV00623	varitome	S. lycopersi	ECU	-2.475	-78.175	11.94301	9.420501	slc_ec
BGV00623	varitome	S. lycopersi	ECU	-2.6	-78.1667	22.93203	17.7693	slc_ec
BGV00623	varitome	S. lycopersi	ECU	-4.01667	-78.8833	16.04711	13.23002	slc_ec
BGV00623	varitome	S. lycopersi	ECU	-3.06667	-78.4667	23.68724	19.04989	slc_ec
BGV00632	varitome	S. pimpinel	PER	-5.10238	-80.7652	6.450458	4.304	sp_pe
BGV00633	varitome	S. pimpinel	PER	-5.28524	-80.5527	8.036124	5.394539	sp_pe
BGV00634	varitome	S. pimpinel	PER	-5.15833	-79.175	12.12766	8.442825	sp_pe
BGV00635	varitome	S. pimpinel	PER	-5.58917	-79.9703	2.574504	1.678594	sp_pe
BGV00636	varitome	S. pimpinel	PER	-5.69561	-78.8144	12.21557	7.217473	sp_pe
BGV00637	varitome	S. pimpinel	PER	-4.86318	-80.7734	21.10173	15.77627	sp_x_sp
BGV00645	varitome	S. pimpinel	PER	-5.09651	-80.1605	14.19804	9.4601	sp_pe
BGV00645	varitome	S. pimpinel	PER	-5.2181	-80.1206	22.55628	15.13125	sp_pe
BGV00647	varitome	S. pimpinel	PER	-6.71278	-79.9098	29.54676	20.17249	sp_pe
BGV00675	varitome	S. lycopersi	ECU	-0.90869	-77.8078	16.94015	13.18059	slc_ec
BGV00676	varitome	S. lycopersi	ECU	-0.95722	-77.8158	19.30602	15.0988	slc_ec
BGV00676	varitome	S. lycopersi	ECU	-1.03056	-77.7308	14.53629	11.53683	slc_ec
BGV00677	varitome	S. lycopersi	ECU	-1.03528	-77.6686	7.371354	5.715651	sp_ec

BGV00677	varitome	S. lycopersi	ECU	-1.03528	-77.6686	7.876092	6.188571	slc_ec
BGV00677	varitome	S. lycopersi	ECU	-1.03139	-77.7311	9.07283	7.214926	slc_ec
BGV00679	varitome	S. lycopersi	ECU	-0.99242	-77.8152	16.93803	13.44227	slc_ec
BGV00680	varitome	S. lycopersi	ECU	-1.16583	-77.8569	7.515401	5.732748	slc_ec
BGV00682	varitome	S. lycopersi	ECU	-1.26333	-77.89	17.31172	13.2663	
BGV00682	varitome	S. lycopersi	ECU	-1.31667	-77.8833	32.18366	23.56371	slc_ec
BGV00685	varitome	S. lycopersi	ECU	-1.7	-77.8333	24.30285	19.49136	slc_ec
BGV00685	varitome	S. lycopersi	ECU	-1.71667	-77.8667	15.52809	12.69961	slc_ec
BGV00686	varitome	S. lycopersi	ECU	-1.80917	-77.8297	9.420263	7.455241	slc_ec
BGV00686	varitome	S. lycopersi	ECU	-1.85607	-77.8145	11.21593	8.954478	slc_ec
BGV00688	varitome	S. lycopersi	ECU	-2.3	-78.1167	7.106069	5.548696	slc_ec
BGV00689	varitome	S. lycopersi	ECU	-2.45864	-78.1772	15.15268	12.09472	slc_ec
BGV00689	varitome	S. lycopersi	ECU	-2.56861	-78.1708	11.51238	9.002741	slc_ec
BGV00690	varitome	S. lycopersi	ECU	-2.56861	-78.1708	6.936908	5.484709	slc_ec
BGV00690	varitome	S. lycopersi	ECU	-2.62278	-78.2027	5.714697	4.65422	slc_ec
BGV00690	varitome	S. lycopersi	ECU	-2.61667	-78.1833	42.16825	33.18244	slc_ec
BGV00690	varitome	S. lycopersi	ECU	-2.61667	-78.1833	0.005942	0.004587	slc_ec
BGV00691	varitome	S. lycopersi	ECU	-2.73417	-78.3192	20.46413	15.87092	slc_ec
BGV00692	varitome	S. lycopersi	ECU	-4.1225	-78.63	18.59748	13.8354	slc_ec
BGV00693	varitome	S. lycopersi	ECU	-4.1225	-78.63	24.62309	19.81543	slc_ec
BGV00693	varitome	S. lycopersi	ECU	-3.82389	-78.7681	14.30648	11.19373	slc_ec
BGV00701	varitome	S. lycopersi	ECU	-1.42472	-78.4356	14.09119	10.88849	slc_ec
BGV00701	varitome	S. lycopersi	ECU	-1.40278	-78.3011	10.29783	7.590394	slc_ec
BGV00702	varitome	S. lycopersi	ECU	-1.80972	-78.0331	18.01777	13.95967	slc_ec
BGV00710	varitome	S. pimpinel	ECU	0.99609	-79.55	15.75375	11.95111	sp_ec
BGV00711	varitome	S. pimpinel	ECU	0.98457	-79.5629	6.826511	5.066307	sp_ec
BGV00714	varitome	S. pimpinel	ECU	-0.18479	-79.5132	12.74179	9.720311	sp_ec
BGV00715	varitome	S. pimpinel	ECU	-0.12583	-79.5853	14.44543	11.18677	sp_ec
BGV00715	varitome	S. pimpinel	ECU	-0.07873	-79.7089	14.77989	11.40909	sp_ec
BGV00715	varitome	S. pimpinel	ECU	-0.04053	-79.7441	17.12702	12.38357	sp_ec
BGV00716	varitome	S. pimpinel	ECU	0.073686	-80.0523	19.01205	14.68149	sp_ec
BGV00716	varitome	S. pimpinel	ECU	0.073686	-80.0523	20.62193	16.05819	sp_ec
BGV00718	varitome	S. pimpinel	ECU	0.075231	-80.0274	20.18132	15.89742	sp_ec
BGV00719	varitome	S. pimpinel	ECU	-0.13628	-80.2281	8.015581	6.305886	sp_ec
BGV00719	varitome	S. pimpinel	ECU	-0.20157	-80.2639	12.08211	8.999273	sp_x_sp
BGV00733	varitome	S. pimpinel	ECU	-3.33333	-79.85	7.61684	5.395299	sp_ec
BGV00736	varitome	S. pimpinel	ECU	-3.32519	-79.5949	9.287456	6.962816	sp_ec
BGV00785	varitome	S. lycopersi	MEX	21.30667	-89.2597	4.942055	3.877821	sll_mx
BGV00785	varitome	S. lycopersi	MEX	21.07611	-89.6306	9.977293	8.124598	sll_mx
BGV00786	varitome	S. lycopersi	MEX	21.24722	-89.0431	10.69893	8.116653	sll_mx
BGV00786	varitome	S. lycopersi	MEX	21.41861	-88.6131	17.40977	13.58932	sll_mx
BGV00786	varitome	S. lycopersi	MEX	21.41861	-88.6131	11.24332	7.618303	sll_mx
BGV00786	varitome	S. lycopersi	MEX	21.41861	-88.6131	15.22547	12.11526	sll_mx
BGV00786	varitome	S. lycopersi	MEX	21.41861	-88.6131	23.20671	19.17038	sll_mx
BGV00786	varitome	S. lycopersi	MEX	20.88333	-89.75	21.38072	17.08047	sll_mx

BGV00787(	varitome	S. lycopersi	MEX	20.48472	-89.7131	18.61977	15.22803	sll_mx
BGV00787:	varitome	S. lycopersi	MEX	20.48472	-89.7131	9.484245	7.780494	sll_mx
BGV00787:	varitome	S. lycopersi	MEX	20.30278	-89.4183	5.679702	4.631142	sll_mx
BGV00787!	varitome	S. lycopersi	MEX	21.00833	-89.0083	8.579358	6.966827	sll_mx
BGV00787(	varitome	S. lycopersi	MEX	20.69056	-88.2014	14.43548	11.7625	sll_mx
BGV00787!	varitome	S. lycopersi	MEX	19.45833	-89.7314	18.14719	15.16397	sll_mx
BGV00789·	varitome	S. lycopersi	MEX	20.17438	-98.0594	30.45317	24.58271	slc_ma
BGV00789!	varitome	S. lycopersi	MEX	20.27222	-97.9547	20.84167	16.95699	sll_mx
BGV00789!	varitome	S. lycopersi	MEX	20.17472	-98.0525	18.5548	14.72729	slc_ma
BGV00790(	varitome	S. lycopersi	MEX	20.17472	-98.0525	18.75458	15.0407	slc_world
BGV00790:	varitome	S. lycopersi	MEX	20.17472	-98.0525	14.95911	11.27732	slc_ma
BGV00790:	varitome	S. lycopersi	MEX	20.17472	-98.0525	19.19628	14.61164	slc_ma
BGV00790!	varitome	S. lycopersi	MEX	21.0525	-98.5078	7.877734	6.127787	slc_ma
BGV00790!	varitome	S. lycopersi	MEX	21.14028	-98.42	16.32733	12.7487	slc_ma
BGV00791(	varitome	S. lycopersi	MEX	21.98194	-99.0097	10.42179	8.198153	slc_ma
BGV00791:	varitome	S. lycopersi	MEX	21.26111	-98.7917	9.543288	7.784657	slc_ma
BGV00791!	varitome	S. lycopersi	MEX			5.130832	4.077885	slc_ma
BGV00792(	varitome	S. lycopersi	MEX	21.38611	-98.9903	17.366	13.77361	slc_ma
BGV00792:	varitome	S. lycopersi	MEX	21.05417	-104.484	15.78248	12.06542	slc_ma
BGV00792:	varitome	S. lycopersi	MEX	24.79917	-107.384	12.49257	9.54055	slc_ma
BGV00793:	varitome	S. lycopersi	MEX	25.91833	-109.171	16.07505	12.41932	slc_ma
BGV00793:	varitome	S. lycopersi	MEX	22.39694	-105.457	14.42032	10.04408	slc_ma
BGV00793·	varitome	S. lycopersi	MEX	21.805	-105.346	15.82788	12.87952	slc_ma
BGV00793!	varitome	S. lycopersi	MEX	21.80488	-105.347	15.77064	10.69338	slc_ma
BGV00793(	varitome	S. lycopersi	MEX	21.00833	-89.5083	23.2032	16.59832	sll_mx
BGV00798:	varitome	S. lycopersi	PER	-9.15306	-74.7764	20.47816	15.6288	slc_pe
BGV00798!	varitome	S. lycopersi	PER	-13.6336	-72.8939	32.98774	25.76636	slc_pe
BGV00799(	varitome	S. lycopersi	PER	-13.9197	-72.175	19.06756	15.07347	slc_pe
BGV00799:	varitome	S. pimpinel	PER	-12.6361	-73.0912	29.61685	24.36822	sp_x_sl
BGV00803(	varitome	S. lycopersi	PER	-11.252	-74.6384	18.69843	14.76351	slc_pe
BGV00803:	varitome	S. lycopersi	PER	-12.6167	-73.7917	12.05136	9.458054	slc_pe
BGV00804:	varitome	S. lycopersi	PER	-12.6167	-73.7833	15.06289	12.19686	slc_pe
BGV00804:	varitome	S. lycopersi	PER	-12.47	-73.83	11.09578	8.276026	sp_x_sl
BGV00805:	varitome	S. lycopersi	MEX	25.25	-98.3167	13.33774	10.43701	slc_ma
BGV00805!	varitome	S. lycopersi	MEX	18.51667	-88.5333	28.09467	23.01371	
BGV00806:	varitome	S. lycopersi	MEX	19.04178	-96.2427	13.2515	10.06568	slc_world
BGV00806!	varitome	S. lycopersi	PER	-6.65	-76.3667	5.086113	4.081053	slc_pe
BGV00806:	varitome	S. lycopersi	MEX	16.85193	-99.8259	3.829522	3.077513	slc_ma
BGV00807(	varitome	S. lycopersi	MEX	22.31136	-97.8608	3.085763	2.368456	slc_ma
BGV00807:	varitome	S. lycopersi	PER	-17.1361	-71.8178	3.963261	3.067825	slc_pe
BGV00809!	varitome	S. lycopersi	PER	-6.14167	-77.0833	3.343137	2.577289	slc_pe
BGV00809(	varitome	S. lycopersi	PER	-6.27167	-76.7733	4.493674	3.554755	slc_pe
BGV00809!	varitome	S. lycopersi	PER	-6.38972	-76.6342	2.701556	2.214738	slc_pe
BGV00810(	varitome	S. lycopersi	PER	-5.90833	-77.7833	3.919656	3.064356	slc_pe
BGV00810(	varitome	S. lycopersi	PER	-14.2583	-68.8297	4.377741	3.59972	slc_pe

BGV00810[illegible]	varitome	S. lycopersi	PER	-12.7675	-71.3664	3.327915	2.754918	
BGV00818[illegible]	varitome	S. lycopersi	PER	-9.31287	-76.0094	2.950559	2.310723	slc_pe
BGV00821[illegible]	varitome	S. lycopersi	CRI	10.01667	-84.2167	2.995301	2.354234	slc_ma
BGV00821[illegible]	varitome	S. lycopersi	CRI	11.06667	-85.6333	23.4538	18.28849	slc_ma
BGV00822[illegible]	varitome	S. lycopersi	SLV	13.81667	-89.4	6.227646	5.21414	slc_ma
BGV00822[illegible]	varitome	S. lycopersi	HND	13.91667	-87.2	12.77627	10.47705	slc_ma
BGV00822[illegible]	varitome	S. lycopersi	NIC	12.05	-85.45	23.91967	19.98713	sll_mx
BGV00822[illegible]	varitome	S. lycopersi	NIC	12.01667	-84.65	26.68235	20.52603	sp_x_sl
BGV00834[illegible]	varitome	S. lycopersi	SLV			15.58619	11.87984	slc_ma
BGV00834[illegible]	varitome	S. lycopersi	CRI	9.930158	-84.0912	20.29461	16.20478	slc_ma
BGV00835[illegible]	varitome	S. lycopersi	CRI	9.930158	-84.0912	17.99195	15.00514	slc_ma
BGV01261[illegible]	varitome	S. lycopersi	PER	-13.1629	-74.2168	11.52202	8.952233	sp_x_sl
BGV01261[illegible]	varitome	S. lycopersi	MEX	20.45606	-97.3168	11.8927	9.883488	slc_ma
BGV01261[illegible]	varitome	S. lycopersi	COL	6.066667	-74.2167	12.84603	10.26274	
BGV01262[illegible]	varitome	S. lycopersi	PER	-12.8645	-72.6965	16.97296	13.22412	sp_x_sl
BGV01262[illegible]	varitome		COL			19.18864	12.4073	sp_x_sl
BGV01262[illegible]	varitome	S. lycopersi	COL			18.52318	15.35125	slc_ma
BGV01263[illegible]	varitome	S. lycopersi	ECU	-2.73935	-79.9124	30.09542	23.59317	slc_ec
BGV01264[illegible]	varitome	S. lycopersi	PER	-13.5526	-72.6069	13.09567	10.54563	slc_pe
BGV01313[illegible]	varitome	S. lycopersi	CRI	9.896162	-83.6576	6.132694	4.944034	slc_ma
BGV01316[illegible]	varitome	S. lycopersi	PER	-6.21667	-76.85	14.07967	11.20788	slc_pe
BGV01317[illegible]	varitome	S. lycopersi	COL	3.662531	-76.2756	13.3241	10.61118	slc_co
BGV01394[illegible]	varitome	S. lycopersi	PER	-12.9092	-71.4036	27.06502	21.97327	slc_pe
BGV01450[illegible]	varitome	S. lycopersi	PER	-6.3898	-76.6332	6.194608	4.780896	slc_pe
BGV01451[illegible]	varitome	S. lycopersi	PER	-6.42111	-76.5177	12.30514	9.757906	slc_pe
BGV01451[illegible]	varitome	S. lycopersi	PER	-6.42111	-76.5177	16.94256	14.00766	slc_pe
BGV01451[illegible]	varitome	S. lycopersi	PER	-6.44658	-76.4738	8.292271	6.685629	slc_pe
BGV01451[illegible]	varitome	S. lycopersi	PER	-6.44944	-76.4717	13.82497	11.43017	slc_pe
BGV01451[illegible]	varitome	S. lycopersi	PER	-6.44944	-76.4717	14.03014	11.56734	slc_pe
BGV01452[illegible]	varitome	S. lycopersi	PER	-6.43778	-76.5806	12.80619	10.01501	slc_pe
BGV01538[illegible]	varitome	S. pimpinel	PER	-5.65807	-78.6882	17.01208	12.64841	sp_montar
BGV01538[illegible]	varitome	S. pimpinel	PER	-5.86638	-78.2785	7.303117	5.5004	sp_montar
BGV01572[illegible]	varitome	S. lycopersi	PER	-6.52611	-76.3011	6.451354	5.34295	slc_pe
BGV01572[illegible]	varitome	S. lycopersi	PER	-6.32444	-76.6831	12.41743	10.28878	slc_pe
BGV01573[illegible]	varitome	S. lycopersi	PER	-6.1274	-76.8439	11.56897	9.301835	slc_pe
BGV01573[illegible]	varitome	S. lycopersi	PER	-6.44658	-76.4738	14.16327	11.74175	slc_pe
CATIE-111[illegible]	varitome		HND			15.97838	12.94452	slc_ma
LA0767	varitome	S. lycopersi	GTM	14.83333	-91.5167	13.82345	11.30587	slc_ma
LA1712	varitome	S. lycopersi	CRI	9.15	-83.5667	13.16337	10.61965	slc_ma
LA2309	varitome	S. lycopersi	PER	-6.825	-76.375	15.81709	12.23488	slc_pe
LA2697	varitome	S. lycopersi	COL			6.762227	5.532674	slc_ma
PAS01447[illegible]	varitome		PER	-5.69333	-78.8017	12.69413	8.933605	sp_montar
PI129026	varitome			-2.16061	-79.9075	17.74875	13.03155	slc_ec
PI129033	varitome			-2.20154	-79.9076	9.965547	7.510184	slc_ec
PI129088	varitome		COL	4.542356	-75.7157	7.981317	6.416269	slc_co

PI378994	varitome	S. lycopersi	PER	-11.9828	-76.7675	7.14336	5.228309	sp_x_sl
PI406890	varitome	S. lycopersi	HND	14.03333	-86.5333	5.98994	4.774212	
PI487625	varitome	S. pimpinel	CRI			10.91439	8.668028	
Tegucigalp	varitome					9.91185	7.065774	sll_vint
Voyage	varitome					4.352049	3.299419	slc_ma

ater than 57
Genetic classification 2
slc_ec_n_600m
sp_ec_n_wet_forest
sp_pe_n_hills
sll_old_cultivars
slc_ec_n_600m
sp_x_sl

sll_modern
sll_vint
sp_x_sl_cherry_cultivars
sll_vint
slc_world
sp_x_sl_cherry_cultivars
sll_vint
sp_x_sl_cherry_cultivars
sll_modern
sp_x_sl_cherry_cultivars
sll_vint
sll_old_cultivars
sp_x_sl
sp_pe_desert_x_sp_pe_n_inter_andean
sp_pe_desert_x_sp_pe_n_inter_andean
sll_vint
sll_vint
sll_vint
sll_vint
sll_vint
sll_vint
sll_vint
sll_vint
sll_old_cultivars
sll_vint
sll_vint_small

sll_modern
sll_old_cultivars
sll_modern
sll_vint

sp_x_sl
sp_pe_desert

sp_pe_desert
sll_vint
sll_vint
sp_x_sl
sll_vint_small
sll_vint
slc_world
sp_x_sl
sll_vint
sll_vint
slc_world
slc_mx_sinaloa
sll_vint
sll_vint
sp_x_sl
sp_x_sl_cherry_cultivars
sll_modern
sll_vint_small
slc_pe_s
sll_vint
sll_vint
sll_vint
sll_old_cultivars

sp_pe_n_hills
sll_old_cultivars
sll_old_cultivars
sll_vint

sll_vint
sll_modern
sll_modern
sp_x_sl
slc_ca
sp_x_sl_cherry_cultivars

sll_vint

sll_vint
sll_vint

sll_vint_small

slc_world
sp_x_sl

slc_pe_n

slc_world
sll_vint
sll_vint
sll_vint_small
sll_modern
sll_vint

sll_modern

sll_old_cultivars
sp_x_sl
sll_vint_small
slc_mx
sp_pe_n_inter-andean

sp_x_sl
sll_vint

sll_vint
sll_modern
sll_vint
slc_mx

sll_modern
sll_vint

sll_vint
sll_vint
sll_vint
sll_vint
sll_old_cultivars
sll_vint
sll_vint

sll_old_cultivars
slc_pe_n
sll_modern
sp_x_sl_cherry_cultivars
sll_vint_small

sll_modern
sll_vint
sll_vint
sll_vint
sll_vint
sll_vint
sll_vint
sll_old_cultivars

sll_vint
sll_vint_small
sll_modern
sp_x_sl

sll_modern
sll_modern
sll_modern
sll_vint_small

slc_world
slc_ec_guayaquil

slc_mx

sp_x_sl
sll_old_cultivars

sll_modern
slc_mx_sinaloa
slc_pe_s
slc_world

sp_pe_desert_x_sp_pe_n_inter_andean
sll_modern
sll_vint
slc_pe_s
sp_x_sl
sll_mx
slc_world
sll_vint_small
sll_vint_small

sp_x_sl_cherry_cultivars
sll_vint
slc_pe_n

sll_vint
sll_vint
sp_x_sl
slc_pe_s
slc_world
sll_vint
sll_vint_small
slc_world

sp_x_sl_cherry_cultivars

sll_modern
slc_world
slc_pe_n
sll_modern
sll_modern

sll_modern
sll_modern
sll_modern
sll_modern
sll_modern

sll_modern
sll_modern
sll_modern
sll_modern

sll_modern
sll_modern
sll_modern
sll_modern
sll_modern
sp_x_sl
slc_ca
sp_x_sl
sp_pe_desert_x_sp_pe_n_inter_andean
sp_ec_s_dry_forest
sp_pe_desert
sp_pe_desert
sp_pe_x_sp_ec
sp_pe_desert
sp_x_sl
sp_pe_desert_x_sp_pe_n_inter_andean

sp_pe_n_inter-andean
sp_pe_desert_x_sp_pe_n_inter_andean
sp_pe_desert
sp_x_sl
sp_pe_desert
sp_pe_desert
sp_x_sl

sp_pe_desert_x_sp_pe_n_inter_andean
sll_old_cultivars
sp_x_sl
sp_x_sl
sp_x_sl
sp_pe_desert
sp_pe_desert
sp_x_sl
sp_pe_desert

sp_pe_desert
sll_vint
sll_vint
sp_x_sl_cherry_cultivars
sll_vint
sll_vint
sll_vint
sll_vint

sll_vint

sll_vint
sll_vint
sll_vint
sll_vint_small
sll_vint
sll_vint
sll_vint
sp_x_sl_cherry_cultivars
sll_vint

sll_vint
sll_vint

sll_old_cultivars
sll_vint

sll_vint
sll_vint
sll_vint
sp_x_sl_cherry_cultivars
sll_vint
sll_vint

sll_vint
sll_vint
sll_vint_small

sp_x_sl_cherry_cultivars
sll_vint
slc_ec_guayaquil
sll_vint
sll_vint

sll_vint
sll_vint
sll_old_cultivars
slc_mx
sp_x_sl
sll_vint
sll_old_cultivars

sll_vint
slc_world
sll_old_cultivars
sll_vint

sll_vint
sp_x_sl_cherry_cultivars
sll_old_cultivars
sll_vint

sll_vint
sll_vint

sll_vint
sll_vint_small
sll_vint
sll_vint
sll_vint
sll_old_cultivars
sll_vint
sll_vint
sll_vint
slc_pe_s
sll_vint_small
sll_old_cultivars
sll_vint
sll_old_cultivars
sll_vint_small
sll_vint
sll_old_cultivars
sll_vint
sll_vint
sll_vint
sp_x_sl
sll_old_cultivars
sll_vint
sll_vint
sll_vint

sll_vint
sll_vint
sll_modern
sll_vint
sll_vint
sll_vint
sll_vint
sll_vint
sp_x_sl_cherry_cultivars
sll_vint
sll_vint

sp_x_sl_cherry_cultivars
sp_x_sl_cherry_cultivars
sll_vint
sll_vint
sll_old_cultivars
sll_modern
sp_x_sl_cherry_cultivars
sll_old_cultivars
sll_vint
sll_vint
slc_mx
sll_vint
sll_vint
sll_vint
sp_x_sl_cherry_cultivars

sll_vint_small
sll_vint
sll_vint
sp_x_sl
sll_modern
sll_vint
sll_vint
sll_old_cultivars
sll_vint
sll_vint
sll_vint
sll_vint
sll_vint_small
sll_vint
sll_vint
sll_old_cultivars
sp_x_sl
sll_vint_small
sll_vint
sll_modern
sll_vint_small
sll_vint
sp_x_sl_cherry_cultivars
sll_vint
sll_modern
sp_x_sl_cherry_cultivars
sll_vint
sll_vint
sp_x_sl_cherry_cultivars

sll_vint
sp_x_sl_cherry_cultivars
slc_world
slc_world
sll_modern
sll_modern
sll_modern
sll_modern
sll_modern
sll_modern
sll_modern
sll_modern
sll_modern

sll_modern
sll_vint
sll_vint

sll_vint
sll_vint
sll_vint
sp_x_sl_cherry_cultivars
sll_vint
sll_vint
slc_world
sll_vint
sp_x_sl_cherry_cultivars
sp_x_sl_cherry_cultivars
sll_modern
sp_x_sl_cherry_cultivars
sll_vint
sll_modern
sp_x_sl_cherry_cultivars
sp_x_sl_cherry_cultivars
slc_world
slc_pe_s
sll_vint_small
slc_mx
sp_x_sl
sp_x_sl_cherry_cultivars
sll_vint_small
sll_vint_small
slc_pe_s
slc_world

sll_vint
sp_pe_desert
sll_modern

sll_modern

sll_vint
slc_world
slc_pe_n
sll_modern
sll_vint
sll_vint_small

sll_old_cultivars

sll_vint_small

sll_old_cultivars
sll_vint_small
slc_co
slc_world
slc_ec_s_1000m

slc_ec_guayaquil
sp_ec_s_dry_forest
slc_ec_c_800m
slc_ec_c_800m
slc_ec_c_800m
slc_ec_c_800m
slc_ec_c_800m
slc_ec_s_1000m
slc_ec_c_800m
sp_pe_n_hills
sp_pe_n_hills
sp_pe_n_hills
sp_pe_n_hills
sp_pe_desert
sp_pe_x_sp_ec
sp_pe_n_hills
sp_pe_n_hills
sp_pe_desert
slc_ec_n_600m
slc_ec_n_600m
slc_ec_n_600m
sp_ec_n_wet_forest

slc_ec_n_600m
slc_ec_n_600m
slc_ec_n_600m
slc_ec_n_600m

slc_ec_n_600m
slc_ec_n_600m
slc_ec_c_800m
slc_ec_c_800m
slc_ec_c_800m
slc_ec_c_800m
slc_ec_c_800m
slc_ec_c_800m
slc_ec_c_800m
slc_ec_c_800m
slc_ec_c_800m
slc_ec_c_800m
slc_ec_c_800m
slc_ec_s_1000m
slc_ec_s_1000m
slc_ec_s_1000m
slc_ec_c_800m
slc_ec_c_800m
slc_ec_c_800m
sp_ec_n_wet_forest
sp_ec_n_wet_forest
sp_ec_n_wet_forest
sp_ec_s_dry_forest
sp_ec_n_wet_forest
sp_ec_n_wet_forest
sp_ec_n_wet_forest
sp_ec_n_wet_forest
sp_ec_n_wet_forest
sp_ec_n_wet_forest
sp_pe_x_sp_ec
sp_ec_s_dry_forest
sp_ec_s_dry_forest
sll_mx
sll_mx
sll_mx
sll_mx
sll_mx
sll_mx
sll_mx
sll_mx

sll_mx
sll_mx
sll_mx
sll_mx
sll_mx
sll_mx
slc_mx
sll_mx
slc_mx
slc_world
slc_mx
slc_mx
slc_mx
slc_mx
slc_mx
slc_mx
slc_mx
slc_mx
slc_mx
slc_mx_sinaloa
slc_mx_sinaloa
slc_mx_sinaloa
slc_mx_sinaloa
slc_mx_sinaloa
sll_mx
slc_pe_s
slc_pe_s
slc_pe_s
sp_x_sl
slc_pe_s
slc_pe_s
slc_pe_s
sp_x_sl
slc_mx

slc_world
slc_pe_n
slc_mx
slc_mx
slc_pe_s
slc_pe_n
slc_pe_n
slc_pe_n
slc_pe_n
slc_pe_s

slc_pe_s
slc_ca
slc_mx
slc_mx
slc_mx
sll_mx
sp_x_sl
slc_ca
slc_ca
slc_ca
sp_x_sl
slc_mx

sp_x_sl
sp_x_sl
slc_ca
slc_ec_guayaquil
slc_pe_s
slc_mx
slc_pe_n
slc_co
slc_pe_s
slc_pe_n
slc_pe_n
slc_pe_n
slc_pe_n
slc_pe_n
slc_pe_n
slc_pe_n
sp_pe_n_inter-andean
sp_pe_n_inter-andean
slc_pe_n
slc_pe_n
slc_pe_n
slc_pe_n
slc_mx
slc_ca
slc_ca
slc_pe_n
slc_mx_sinaloa
sp_pe_n_inter-andean
slc_ec_guayaquil
slc_ec_guayaquil
slc_co

sp_x_sl

sll_vint
slc_ca
