# Supplementary material for: Haplotype analyses reveal novel insights into tomato history and domestication driven by long-distance migrations and latitudinal adaptations
Source: Hortic Res. 2022 Feb 19;9:uhac030. doi: 10.1093/hr/uhac030 (PMC8976693; doi:10.1093/hr/uhac030)
Supplement: Web_Material_uhac030 [file web_material_uhac030.zip › Supplementary Table 4.pdf]

Evolutionary model 1:	SP Pe -> SLC Pe -> SLC MA	
Evolutionary model 2:	SP Pe -> SLC MA -> SLC Pe	

Optimized parameters: mean value after 100 boostraps (95% confidence interval)			Parameter meanir
	**Model 1**	**Model 2**	
Population 1	SP Pe	SP Pe	
Population 2	SLC Pe	SLC MA	
Population 3	SLC MA	SLC Pe	
time_before_second_pop_creation	12.77 (6.51-14.48)	12.13 (7.10-14.48)	Time ellapsed afte
time_from_pop3_till_present	0.40 (0.11-0.79)	0.21 (0.14-0.33)	Time ellapsed sin
pop1_size	6.06 (3.14-8.05)	6.36 (3.50-8.05)	Final population 1
pop2_size	1.09 (0.57-1.66)	1.50 (0.74-1.98)	Final population 2
pop3_size	1.96 (0.56-5.40)	1.21 (0.79-1.96)	Final population 3
pop2_initial_size	0.03 (0.01-0.10)	0.02 (0.01-0.10)	Size of the migrati
pop3_initial_size	0.04 (0.01-0.10)	0.04 (0.01-0.10)	Size of the migrati
growth_time	0.01 (0.00-0.01)	0.01 (0.00-0.01)	Time ellapsed sin
inbreeding_coef1	0.67	0.67	Inbreeding for pop
inbreeding_coef2	0.96	0.98	Inbreeding for pop
inbreeding_coef3	0.98	0.96	Inbreeding for pop
migration12	0.03 (0.01-0.08)	0.03 (0.01-0.07)	Migration rate fror
migration13	0.03 (0.01-0.07)	0.03 (0.01-0.14)	Migration rate fror
migration23	0.13 (0.07-0.23)	0.02 (0.01-0.05)	Migration rate fror
migration32	0.11 (0.01-0.48)	0.46 (0.31-0.56)	Migration rate fror
migration21	0.21 (0.02-0.55)	0.16 (0.02-0.51)	Migration rate fror
migration31	0.21 (0.01-0.56)	0.16 (0.01-0.49)	Migration rate fror
log(likelihood):	-28547.9	-18272.2	

ng

er the second population was created (by a migration to a new location from the first one) and before the third
ce the creation of the third population (by a migration from the second one) till present day (2Ne units)
size (relative to the original size of population 1)
size (relative to the original size of population 1)
size (relative to the original size of population 1)
ion that founded population 2 (relative to the original size of population1)
ion that founded population 3 (relative to the original size of population1)
ce the migrants arrived in a new location till the population adquired its final size (2Ne units)
pulation 1 (This parameter was not optimized by maximum likelihood)
pulation 2 (This parameter was not optimized by maximum likelihood)
pulation 3 (This parameter was not optimized by maximum likelihood)
n population 1 to population 2 (2Ne units)
n population 1 to population 3 (2Ne units)
n population 2 to population 3 (2Ne units)
n population 3 to population 2 (2Ne units)
n population 2 to population 1 (2Ne units)
n population 3 to population 1 (2Ne units)

appeared (2Ne units)
