# Supplementary material for: Haplotype analyses reveal novel insights into tomato history and domestication driven by long-distance migrations and latitudinal adaptations
Source: Hortic Res. 2022 Feb 19;9:uhac030. doi: 10.1093/hr/uhac030 (PMC8976693; doi:10.1093/hr/uhac030)
Supplement: Web_Material_uhac030 [file web_material_uhac030.zip › Supplementary Table 5.pdf]

Region	Size (kb)	Number of genes	Introgressed in	Possible SP origir
SL4.0ch01:90,520,618-90,839,177	318	31	SLC Ec	SP Ec
SL4.0ch02:10,710,483-27,309,754	16599	401	SLC Ec	SP Pe
SL4.0ch03:51,304,614-52,313,086	1008	43	SLC Ec	SP Ec
SL4.0ch04:46,687,437-49,496,049	2808	97	SLC Ec and SLC	SP Pe or SP Ec
SL4.0ch04:18,101,627-19,361,664	1260	22	SLC Ec and SLC	SP Pe or SP Ec
SL4.0ch05:5,285,926-5,453,251	167	17	SLC Ec	SP Pe
SL4.0ch06:31,003,139-31,108,535	105	7	SLC Ec	SP Ec
SL4.0ch06:31,448,155-31,658,163	210	7	SLC Ec	SP Pe or SP Ec
SL4.0ch06:3,846,946-4,683,921	836	30	SLC Ec	SP Ec
SL4.0ch07:56,749,054-56,801,963	52	5	SLC Ec	SP Pe or SP Ec
SL4.0ch08:7,331,324-49,663,802	42332	593	SLC Pe	SP Pe
SL4.0ch11:12,901,633-43,568,128	30666	605	SLC Ec	SP Ec
SL4.0ch11:6,247,520-6,301,792	54	3	SLC Ec	SP Ec

Biological functior	Genes with biological functionts that might indicate an involvement in latitudinal adaptation
Defense, root dev	Solyc01g112320: FAR1-RELATED
Many	Solyc02g032185: Nuclear factor Y, might regulate flowering; Solyc02g021650: DNA damag
Defense, seed do	Solyc03g095720: Transcription elongation factor, involved in the control of seed dormancy
flowering, morpho	Solyc04g050720:PHD finger-containing protein. Interacts with BDT1, acts with other PHD p
flowering, morpho	Solyc04g160300: Agamous like; Solyc04g026030: spermidine synthase, might induce early
flowering, morpho	Solyc05g056620: Macrocalyx, agamous-like 8
chloroplast develo	Solyc06g050620: chloroplast development
defense, seconda	Solyc06g050900: response to nematodes
vegetative and flo	Solyc06g161130, Solyc06g009867, Solyc06g009870, Solyc06g009880, Solyc06g009893,
light response	Solyc07g043270: FAR-RED ELONGATED HYPOCOTYL 3
Many	Solyc08g060840: FAR-RED IMPAIRED RESPONSE 1; Solyc08g061130: promotes photor
Many	
trichome anormality	

ge-binding protein 1b. Component of light signal transduction machinery. Involved in repression of photomorpl
and germination
proteins to associate with flowering genes
y flowering

Solyc06g009897, Solyc06g160940, Solyc06g009940; Solyc06g160940: Agamous like; Solyc06g009950: Phc

norphogenesis in light

hogenesis in darkness

otosystem I P700 chlorophyll a apoprotein A2
