# Supplementary figures and images for: Haplotype analyses reveal novel insights into tomato history and domestication driven by long-distance migrations and latitudinal adaptations
Source: Hortic Res. 2022 Feb 19;9:uhac030. doi: 10.1093/hr/uhac030 (PMC8976693; doi:10.1093/hr/uhac030)

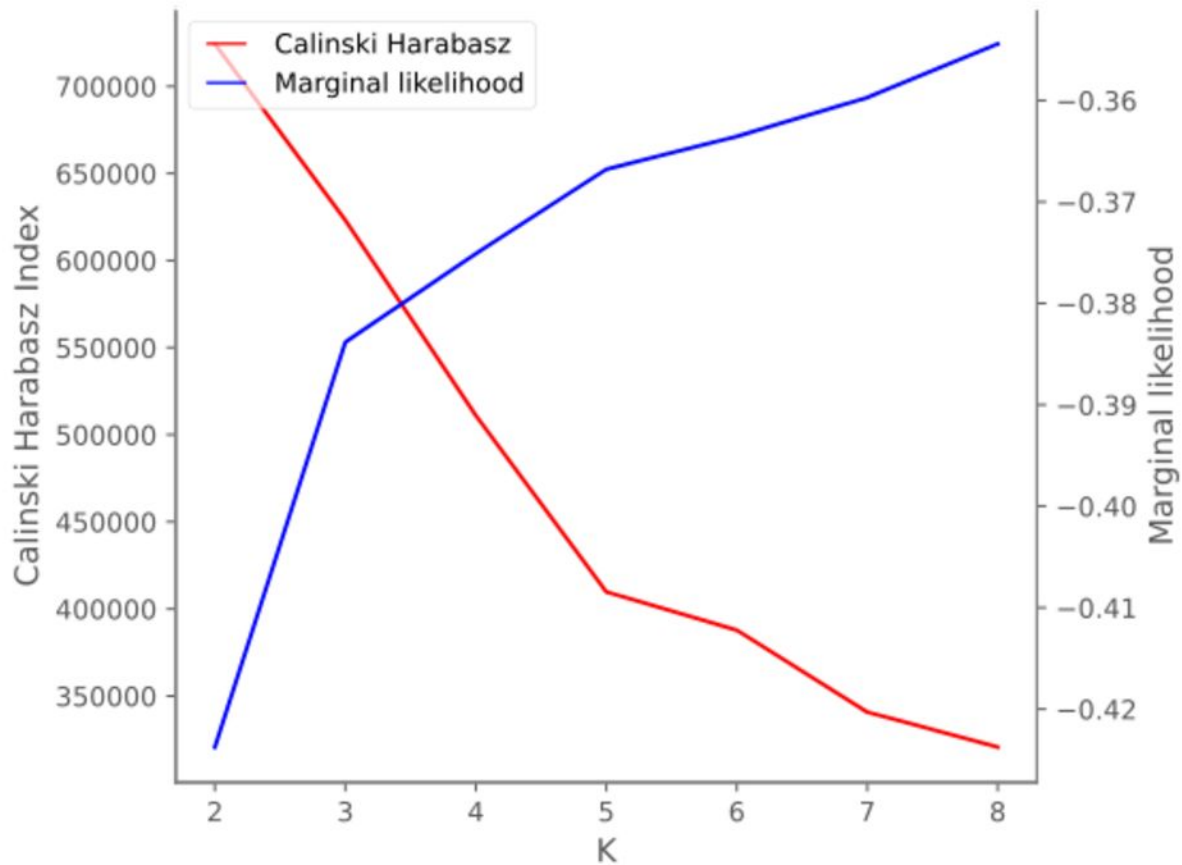

Supplement: Web_Material_uhac030 [file web_material_uhac030.zip › Supplementary figure 1.pdf]

LD: 0.25

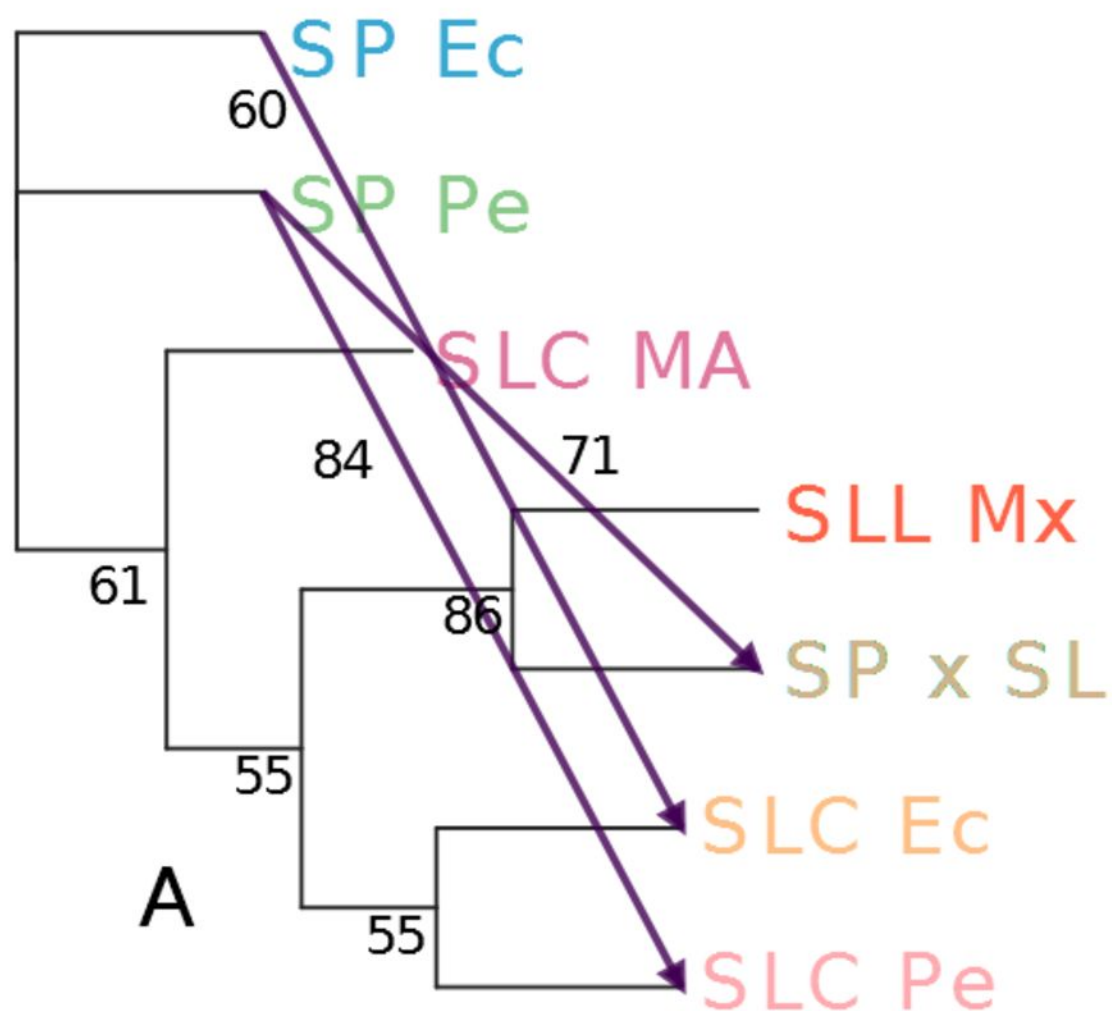

LD: 0.35

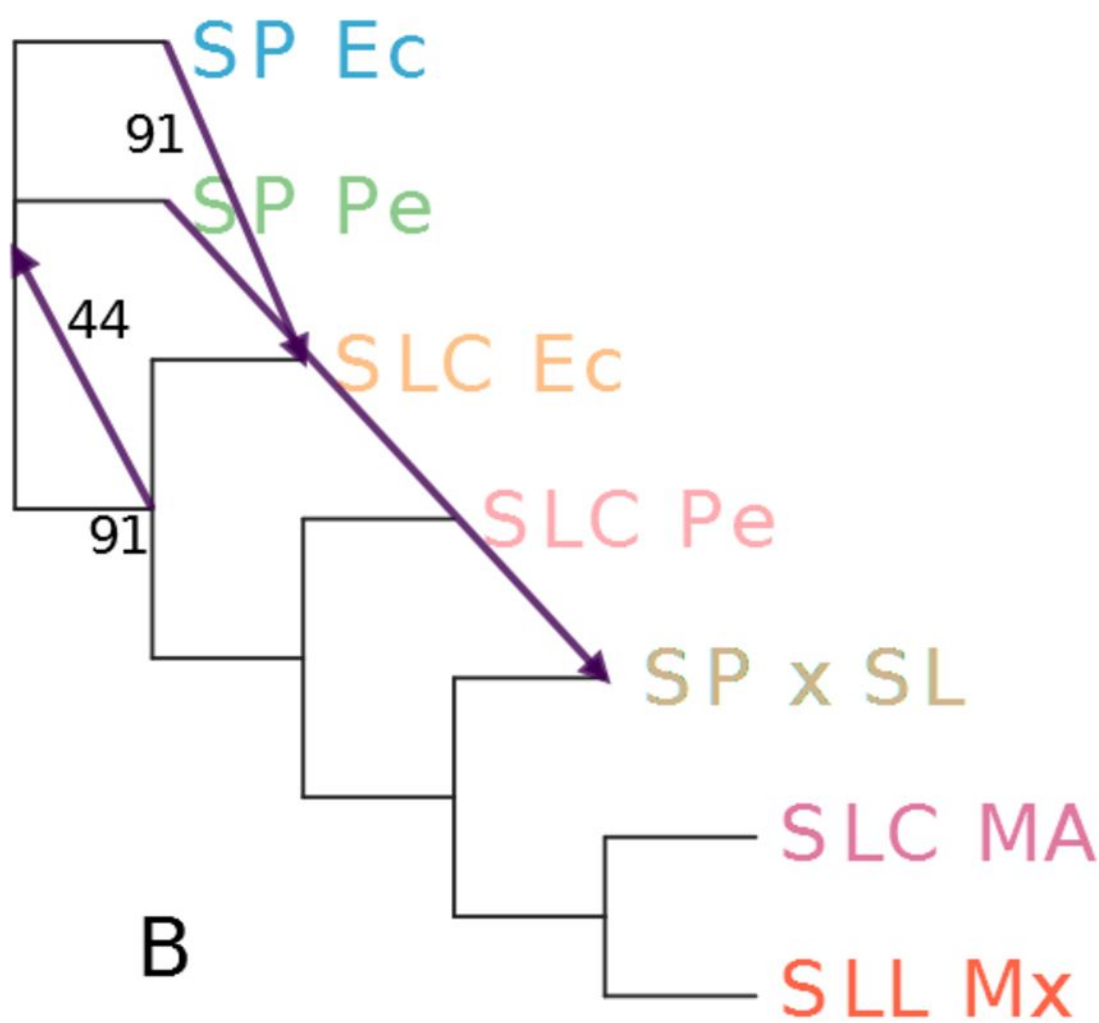

Supplement: Web_Material_uhac030 [file web_material_uhac030.zip › Supplementary figure 10.pdf]

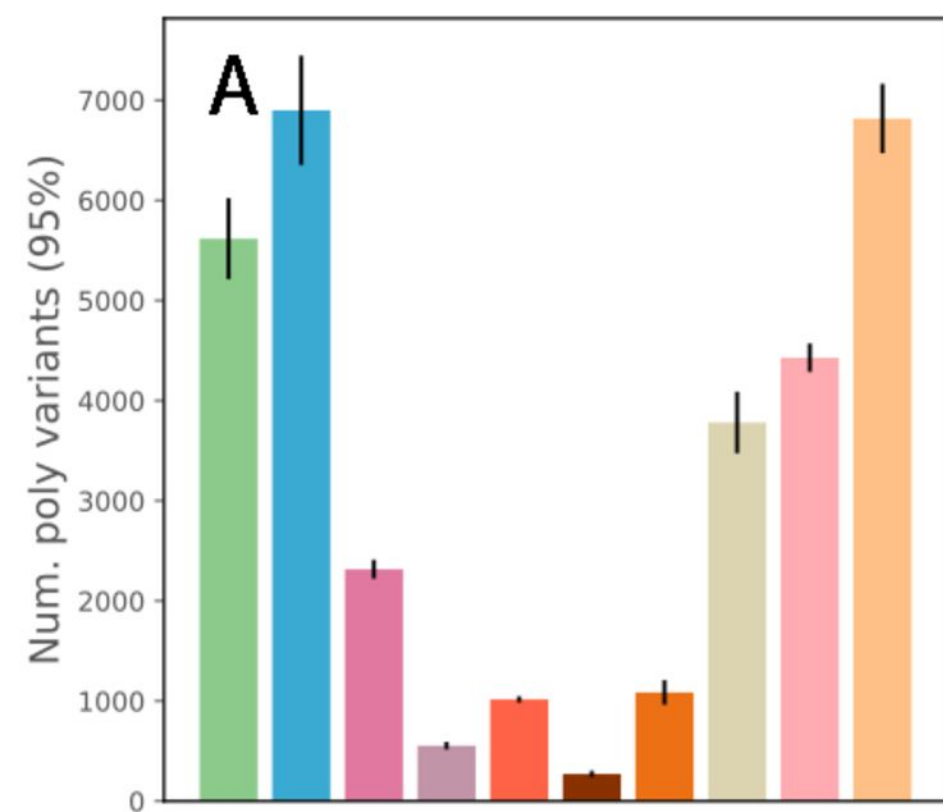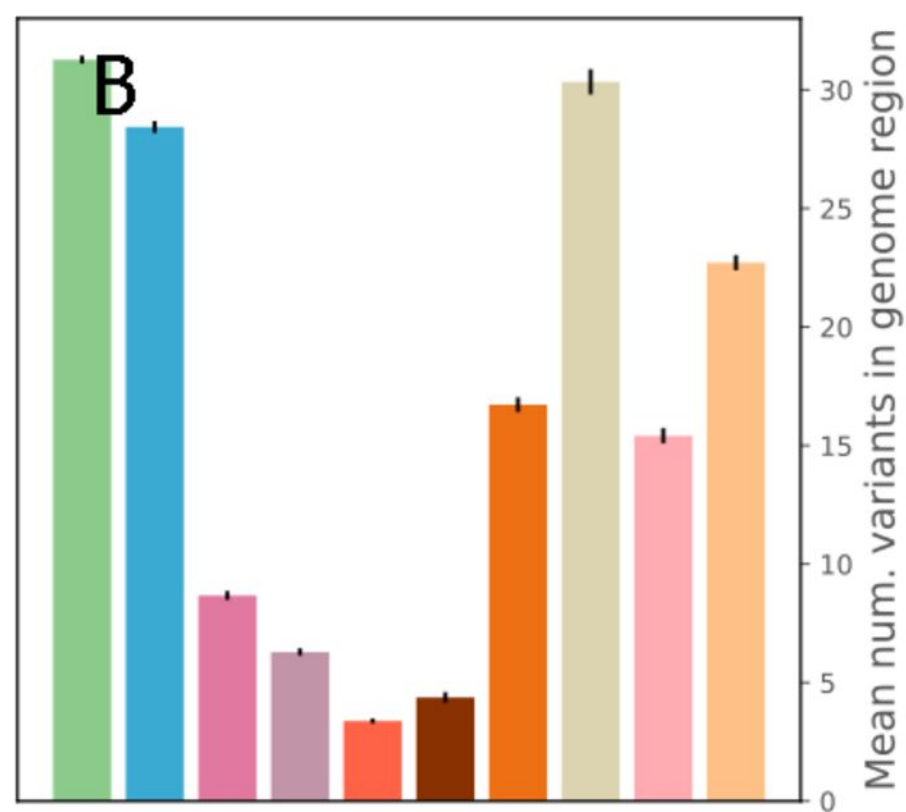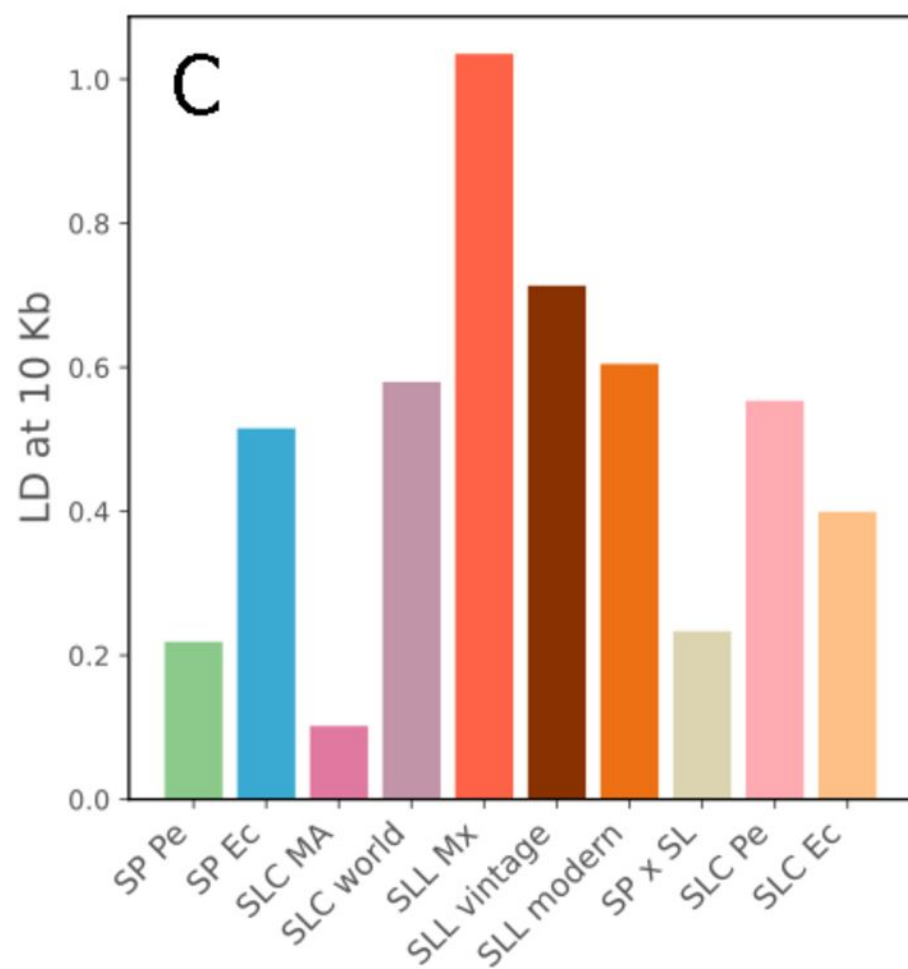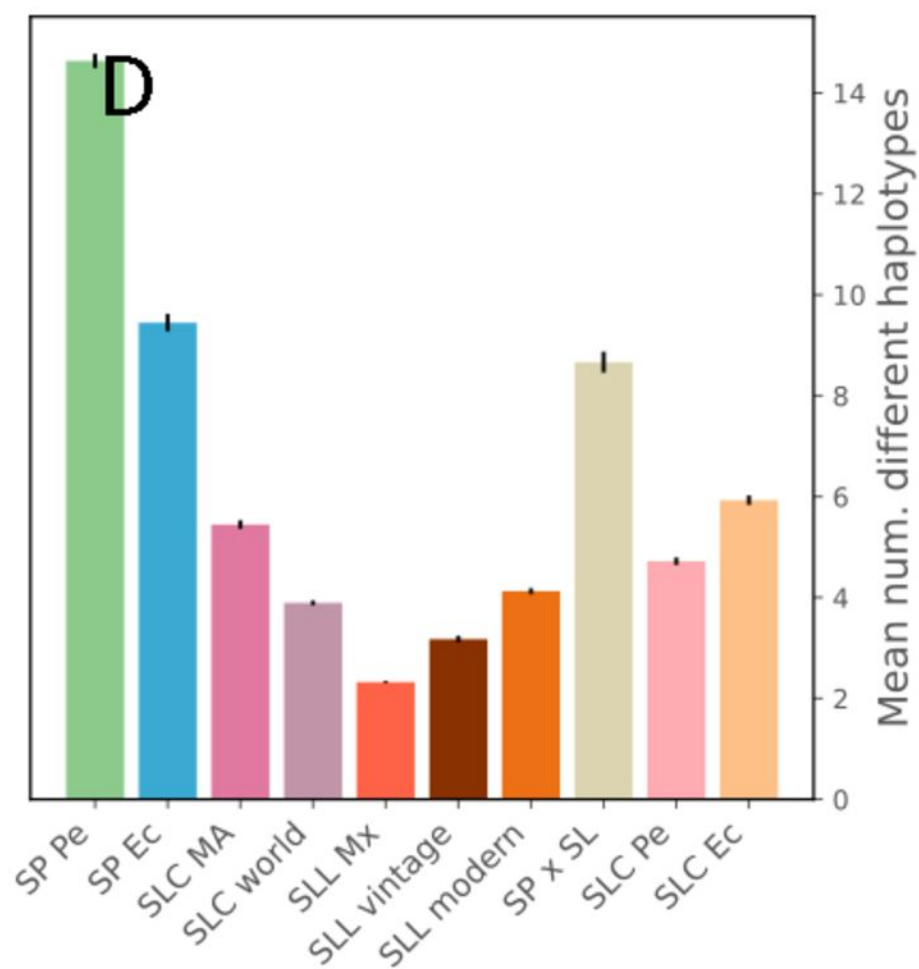

Supplement: Web_Material_uhac030 [file web_material_uhac030.zip › Supplementary figure 11.pdf]

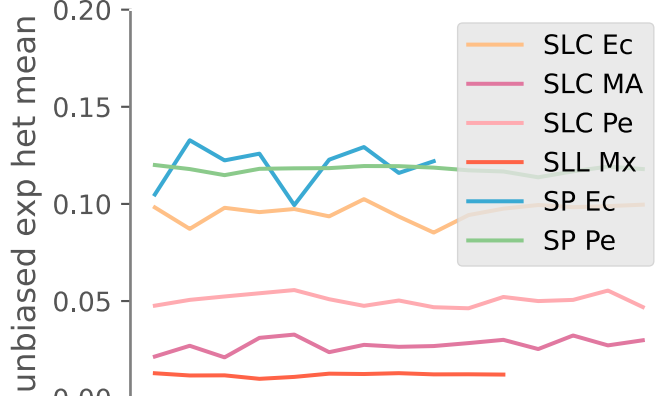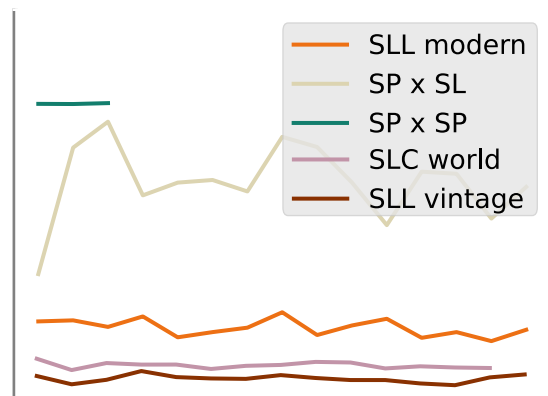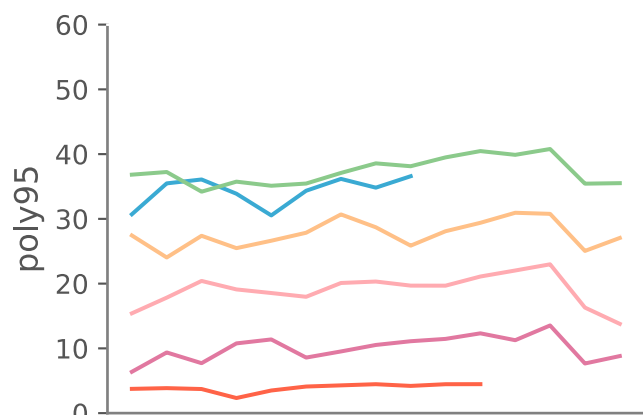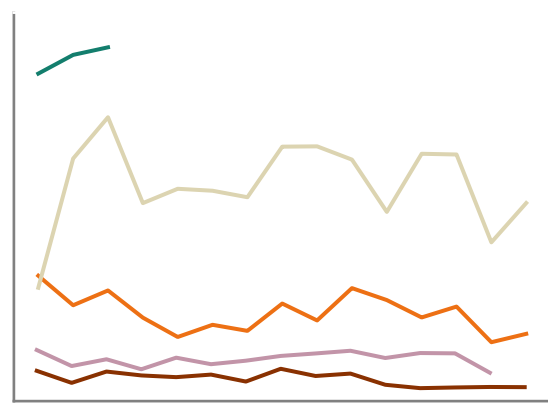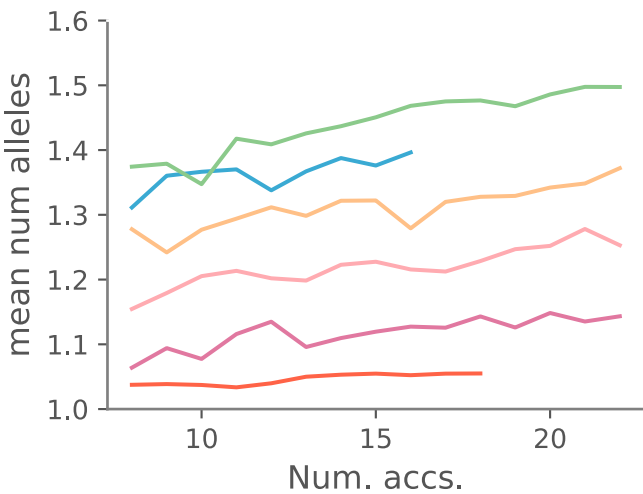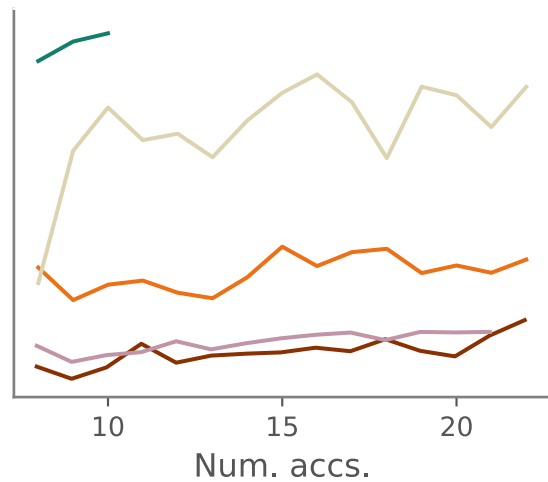

Supplement: Web_Material_uhac030 [file web_material_uhac030.zip › Supplementary figure 12.pdf]

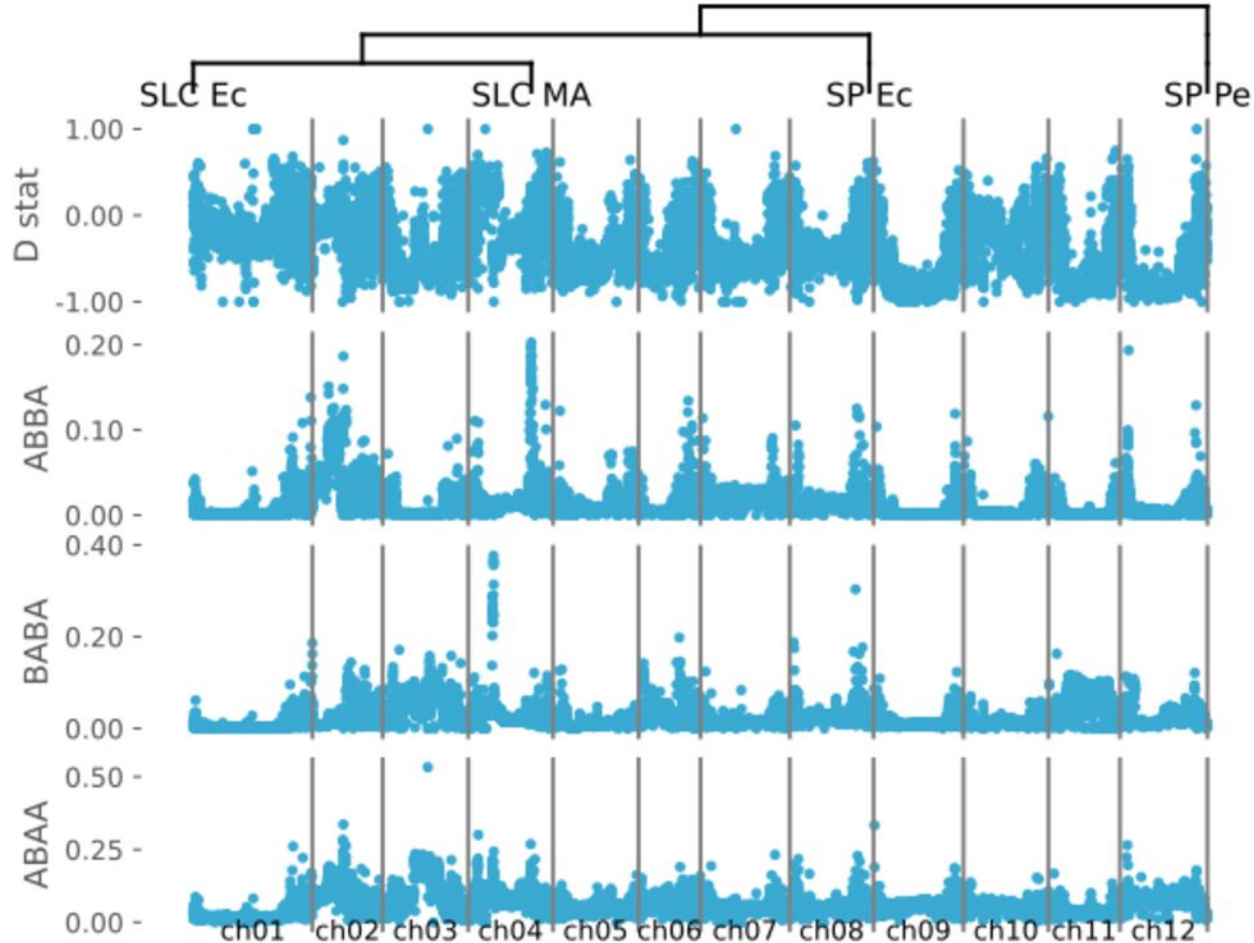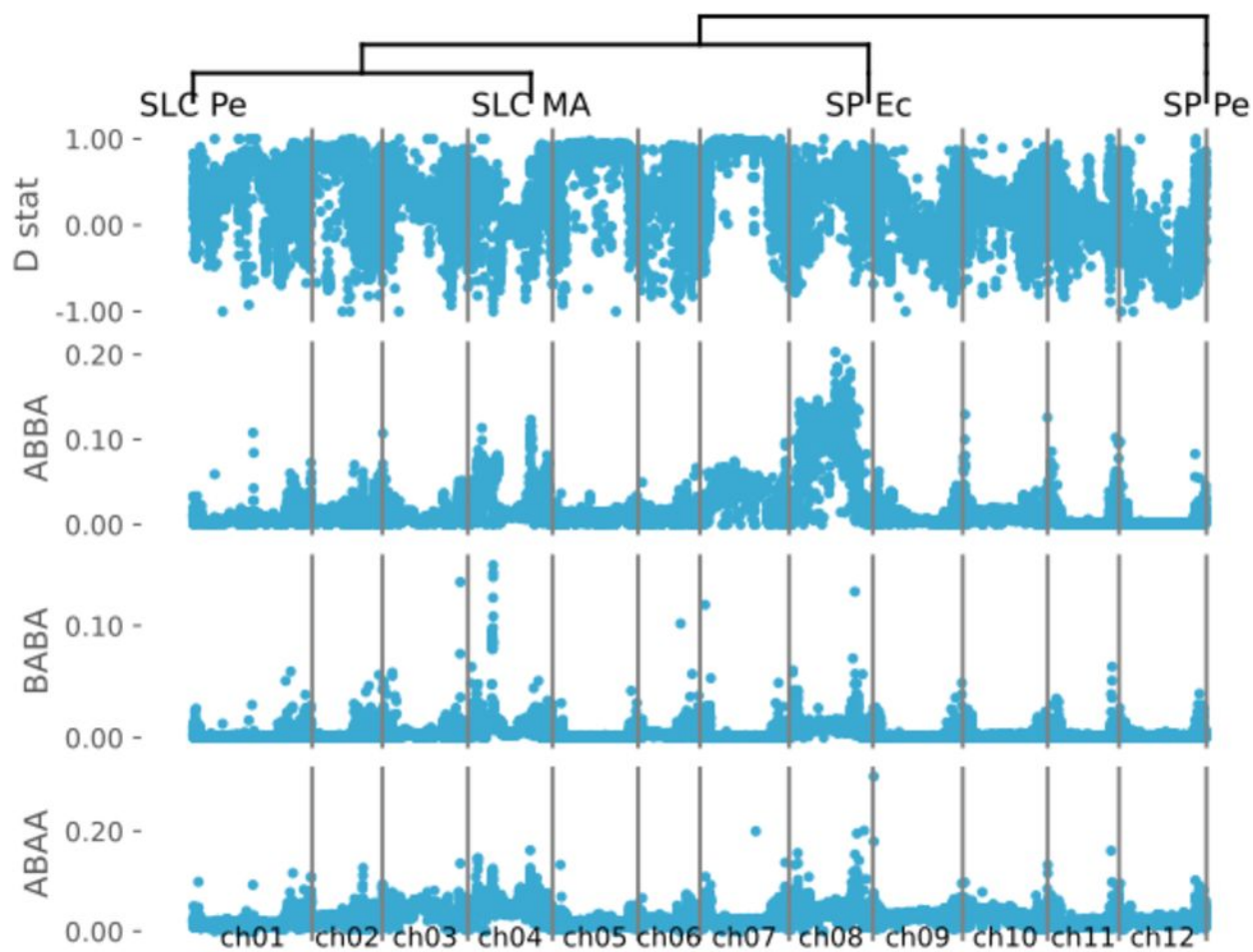

Supplement: Web_Material_uhac030 [file web_material_uhac030.zip › Supplementary figure 13.pdf]

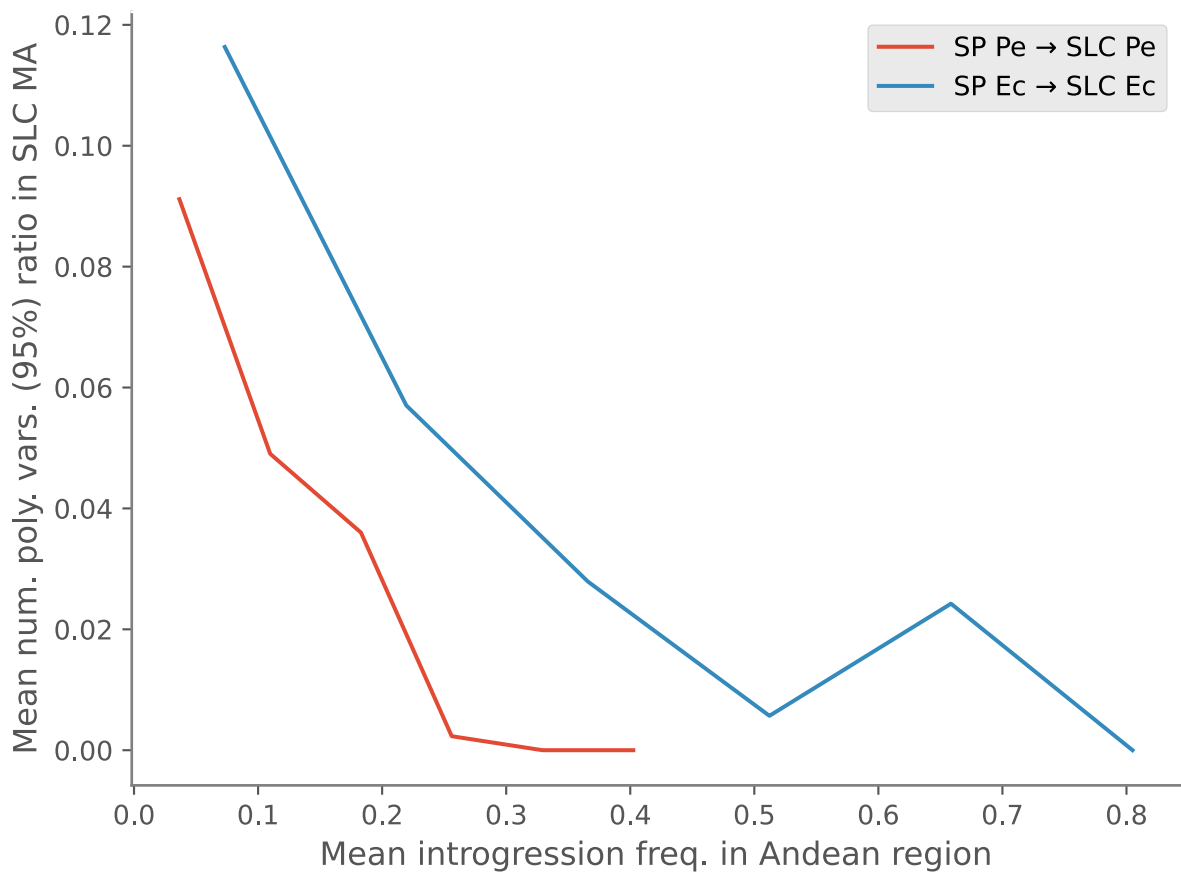

Supplement: Web_Material_uhac030 [file web_material_uhac030.zip › Supplementary figure 14.pdf]

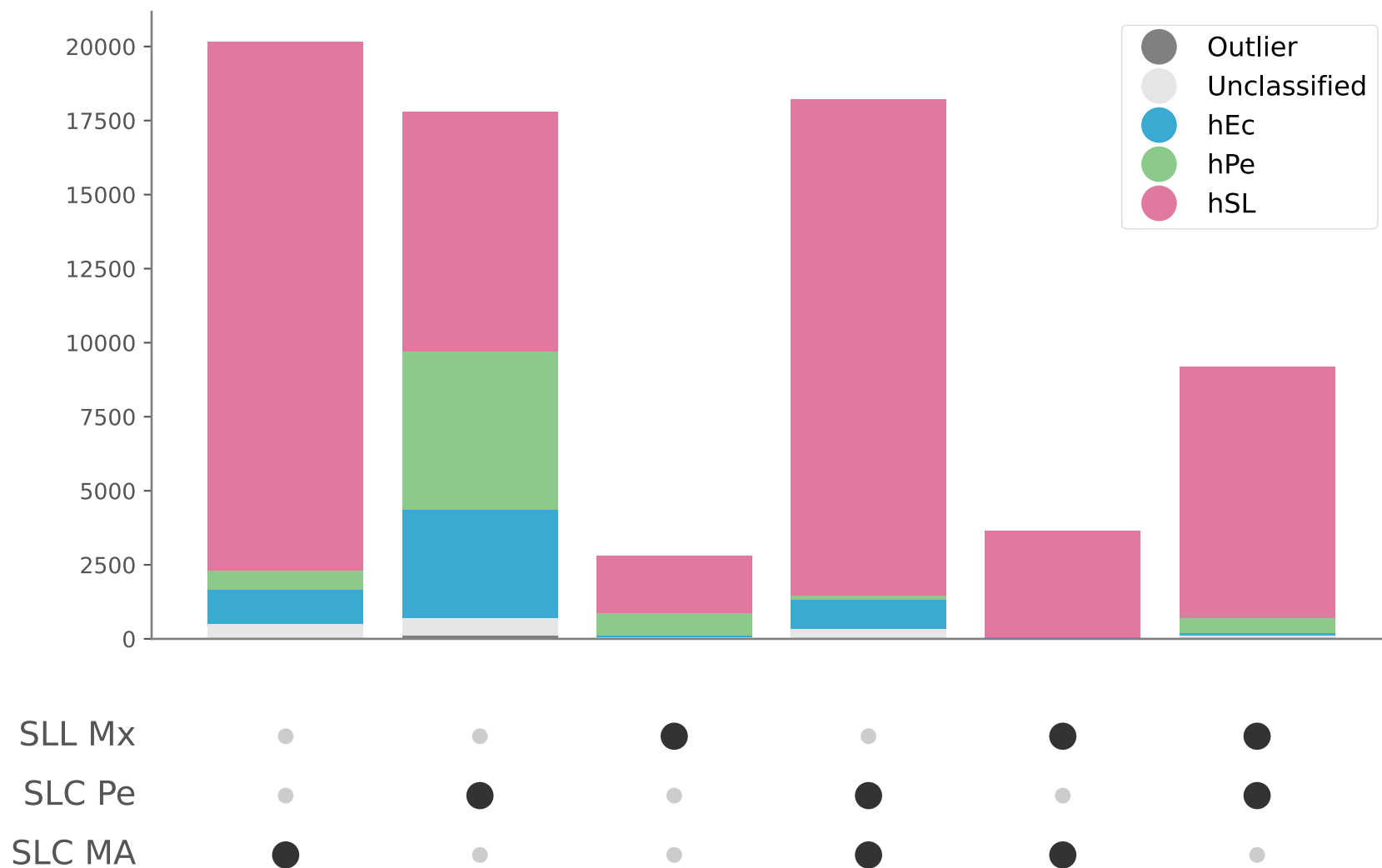

Supplement: Web_Material_uhac030 [file web_material_uhac030.zip › Supplementary figure 15.pdf]

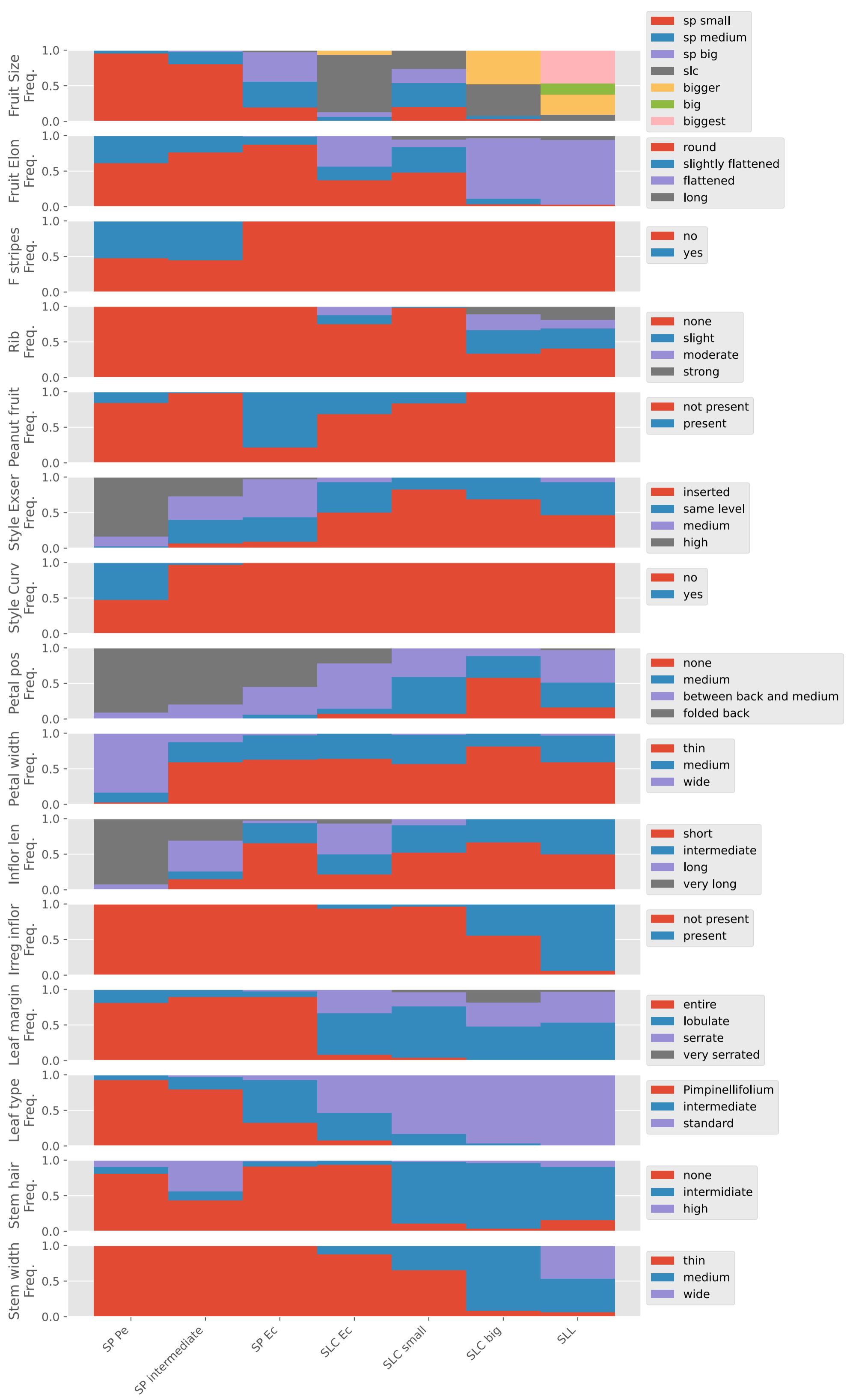

Supplement: Web_Material_uhac030 [file web_material_uhac030.zip › Supplementary figure 17.pdf]

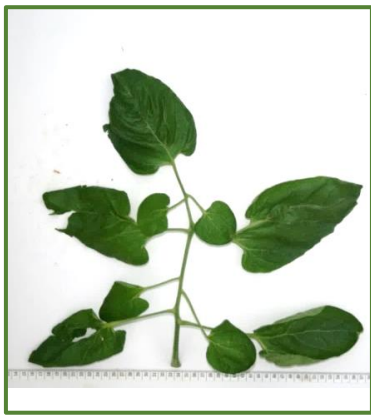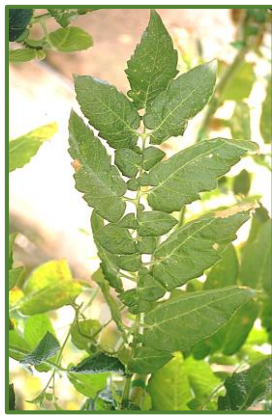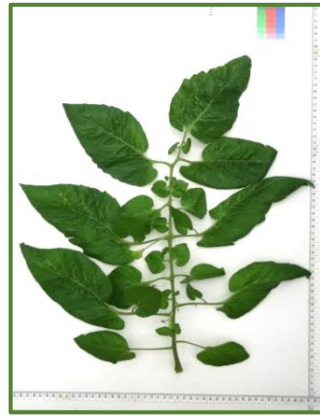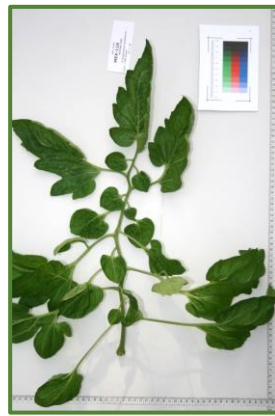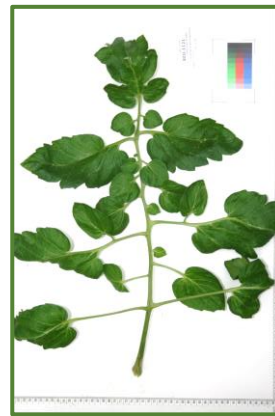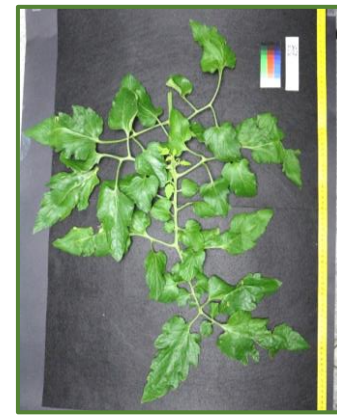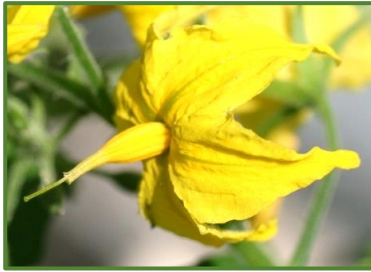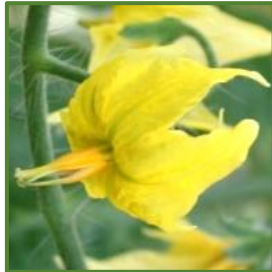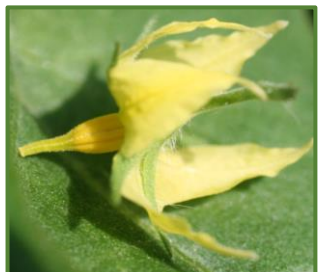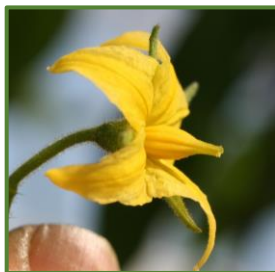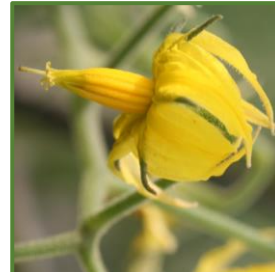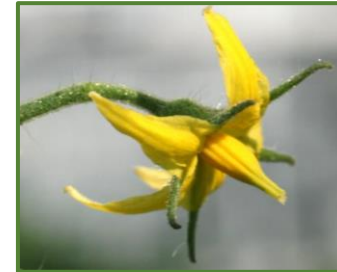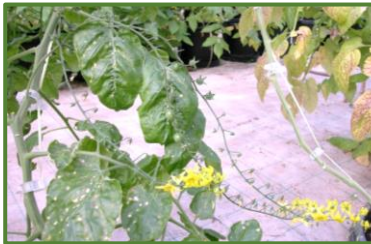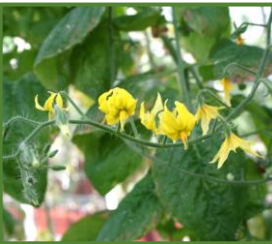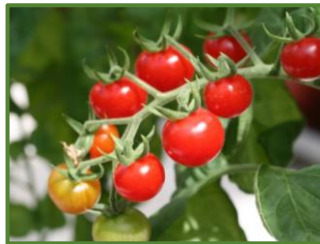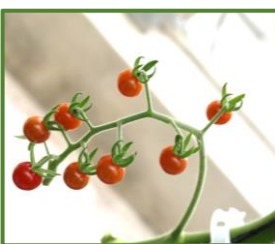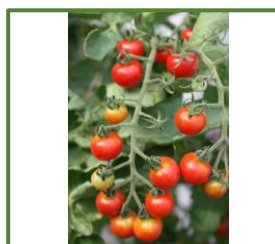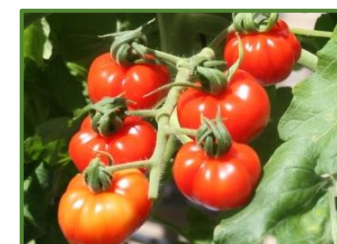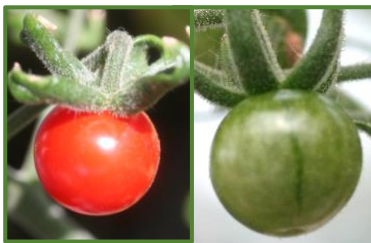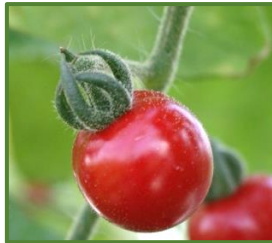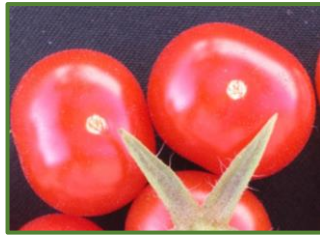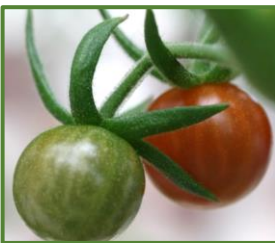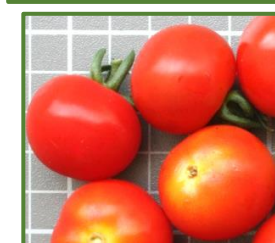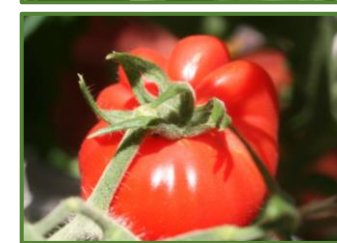

Peruvian SP

Intermediate  
SP

Northern  
Ecuadorian  
SP

SLC-Small

SLC- Ecu

SLC- big

Supplement: Web_Material_uhac030 [file web_material_uhac030.zip › Supplementary figure 18.pdf]

## Slide 1
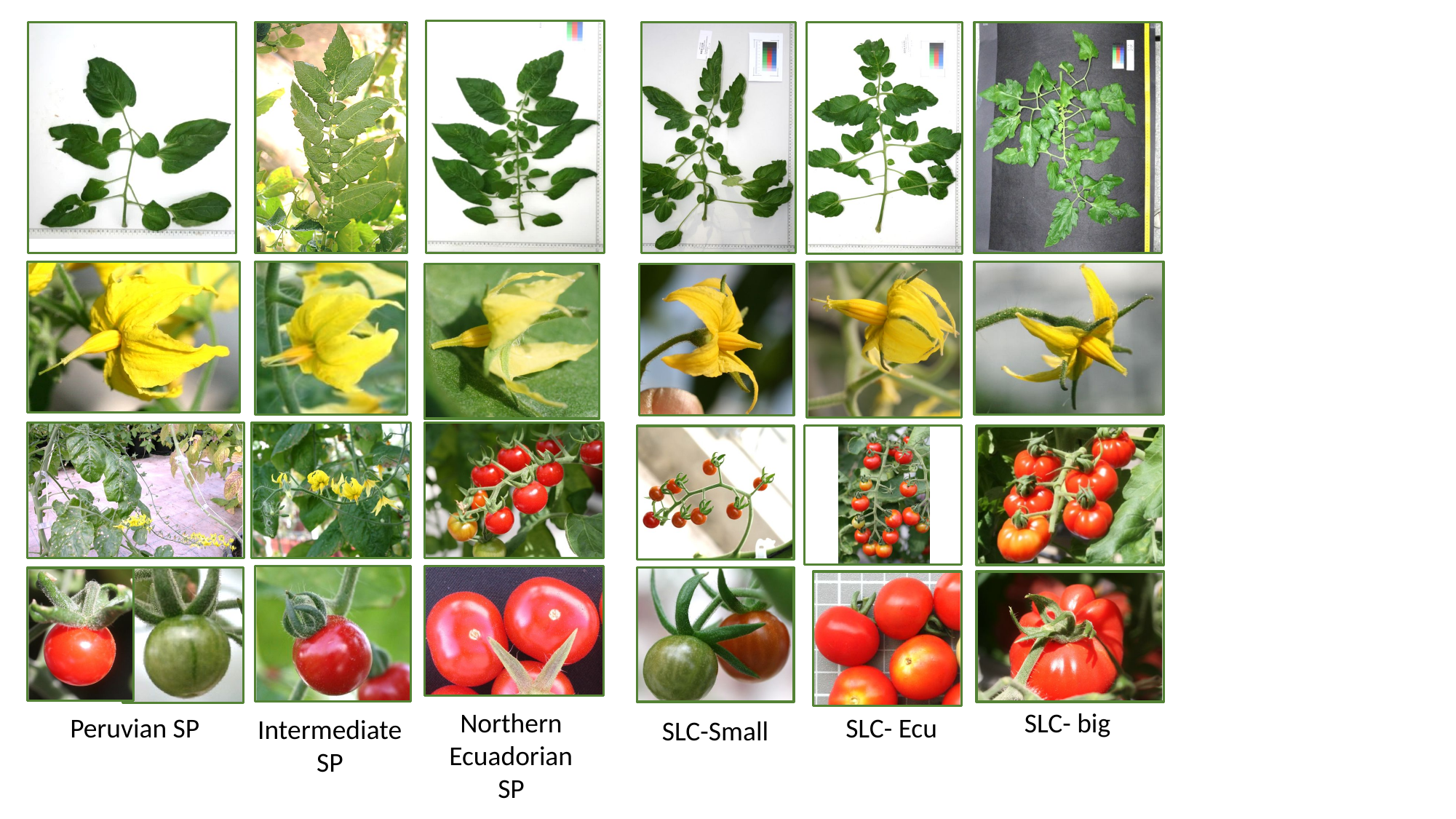

SLC- big
Northern Ecuadorian SP
Peruvian SP
SLC- Ecu
Intermediate SP
SLC-Small

Supplement: Web_Material_uhac030 [file web_material_uhac030.zip › Supplementary figure 18.pptx]

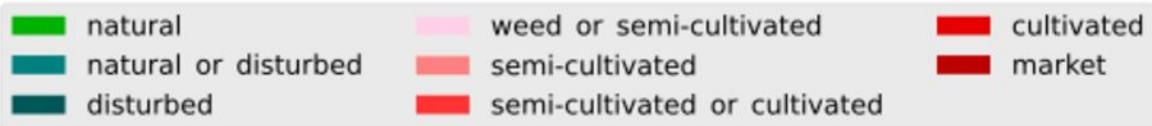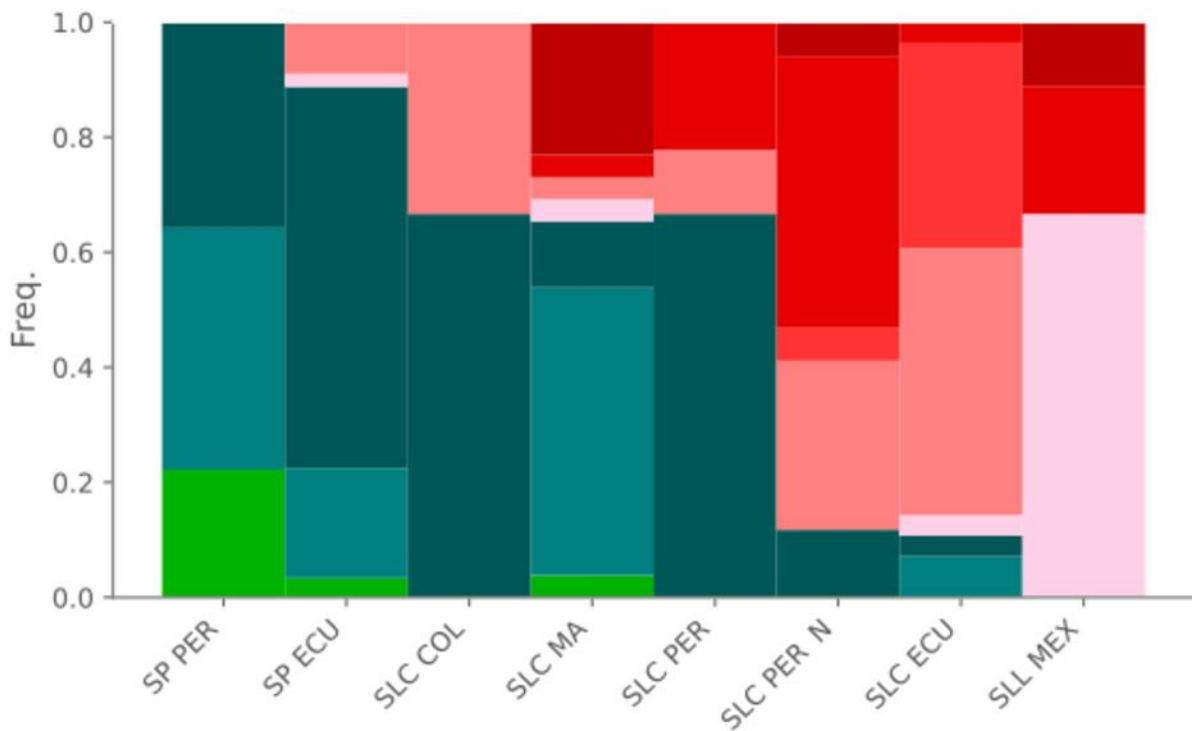

Supplement: Web_Material_uhac030 [file web_material_uhac030.zip › Supplementary figure 19.pdf]

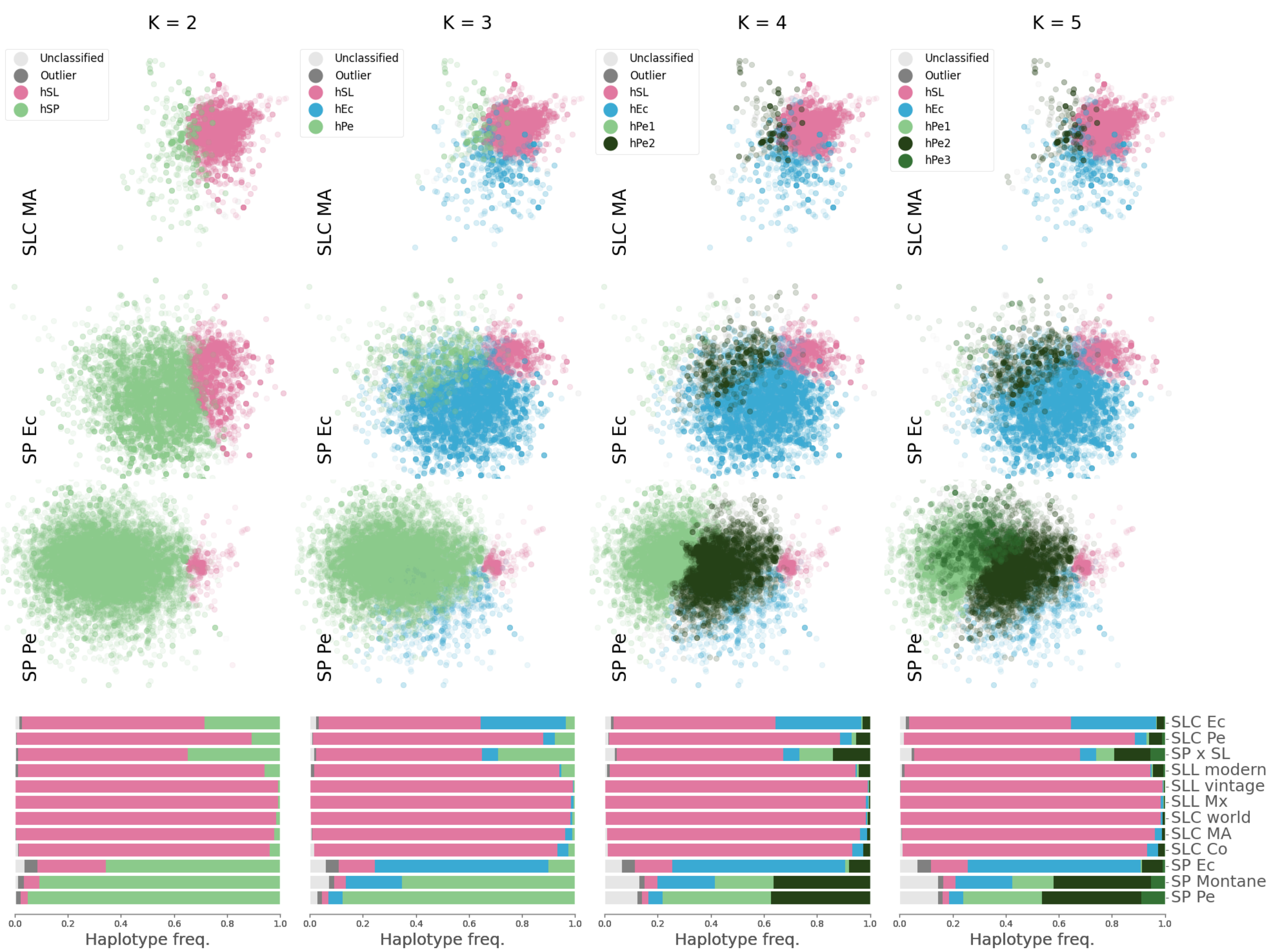

Supplement: Web_Material_uhac030 [file web_material_uhac030.zip › Supplementary figure 2.pdf]

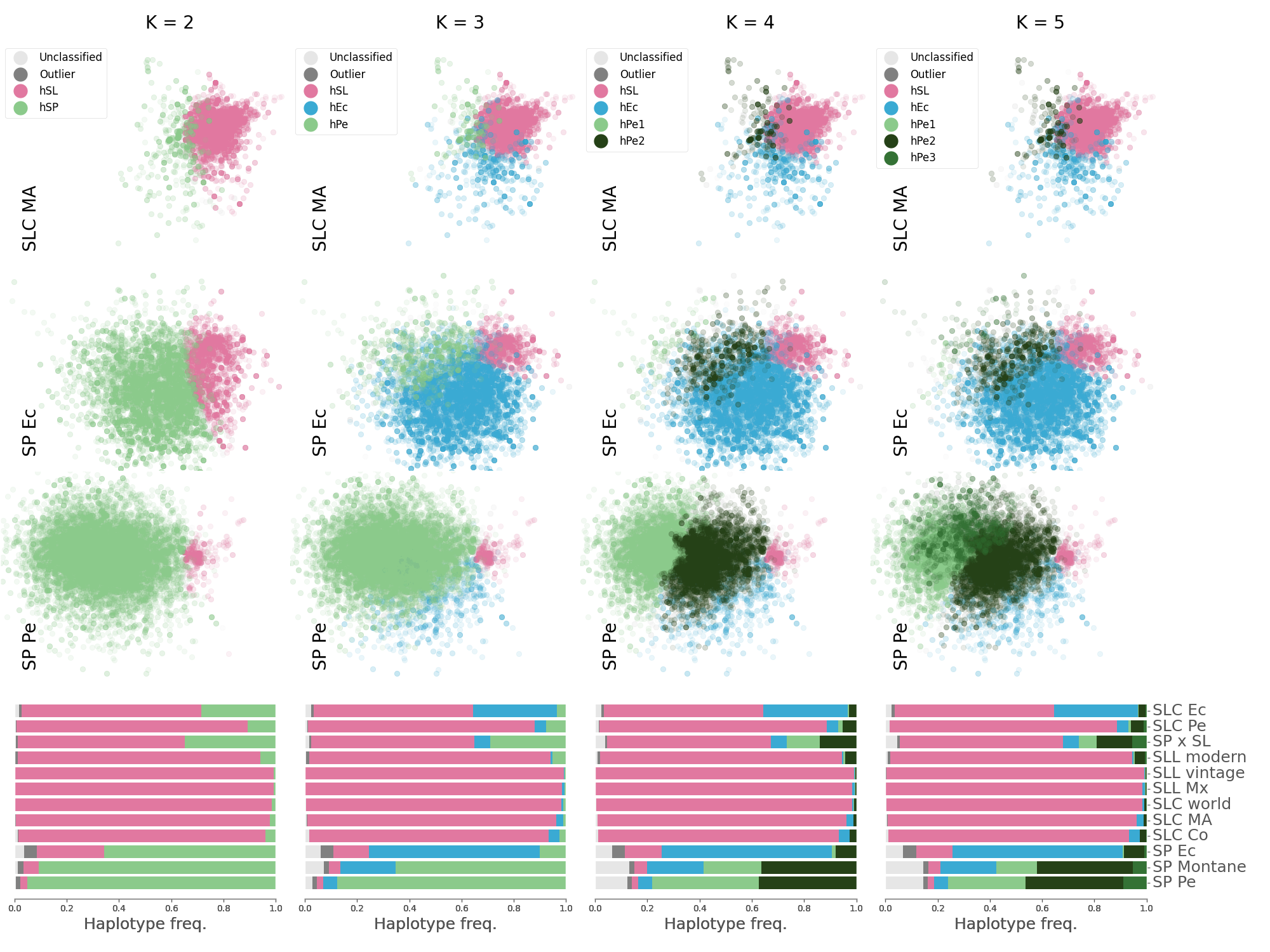

Supplement: Web_Material_uhac030 [file web_material_uhac030.zip › Supplementary figure 2.png]

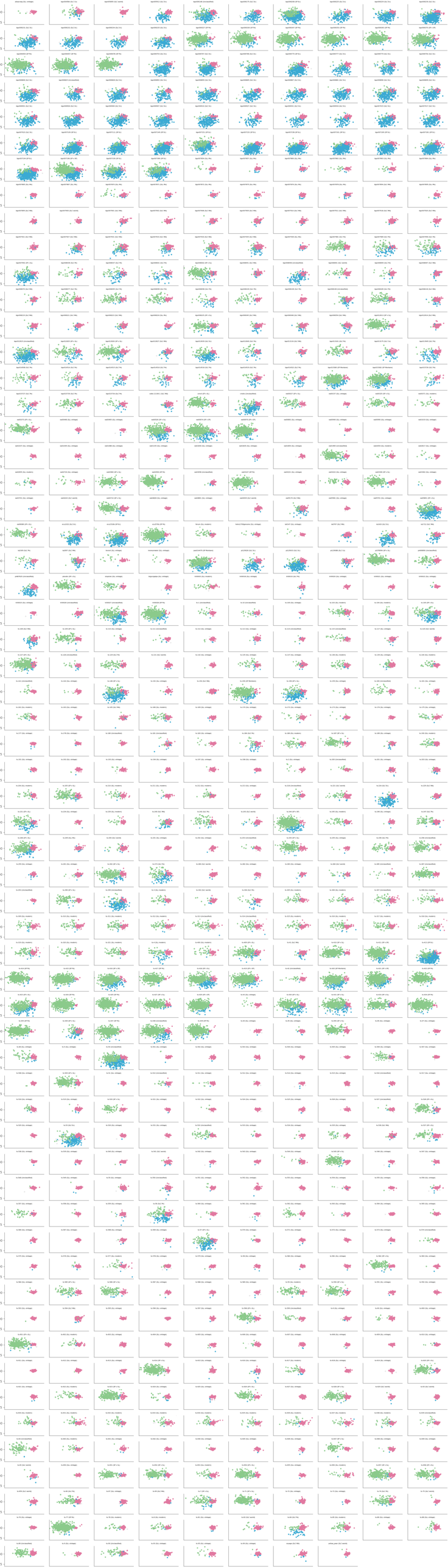

Supplement: Web_Material_uhac030 [file web_material_uhac030.zip › Supplementary figure 3.pdf]

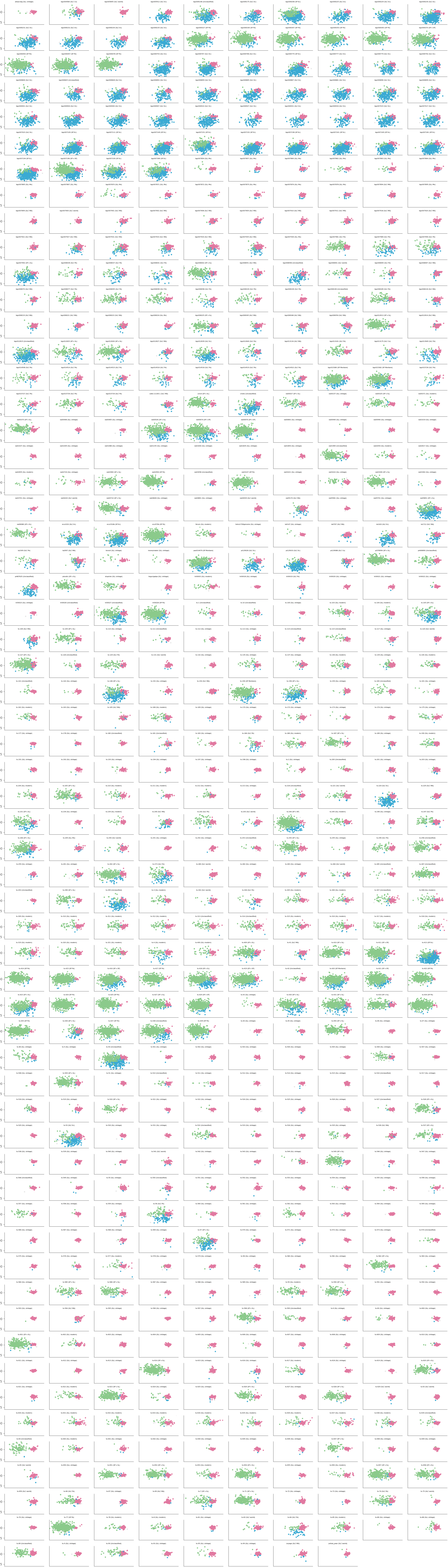

Supplement: Web_Material_uhac030 [file web_material_uhac030.zip › Supplementary figure 3.png]

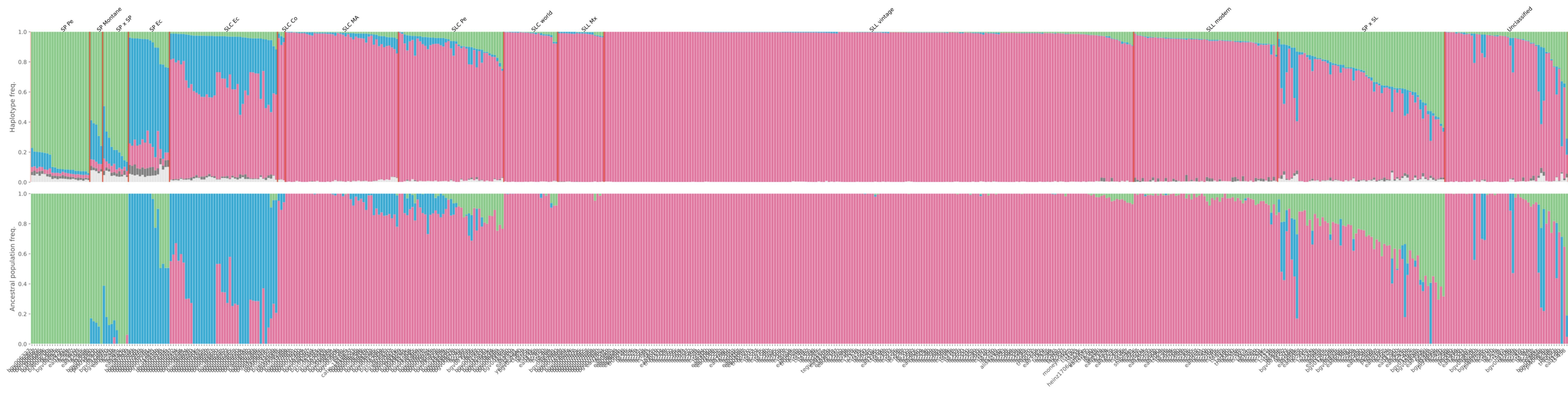

Supplement: Web_Material_uhac030 [file web_material_uhac030.zip › Supplementary figure 4.pdf]

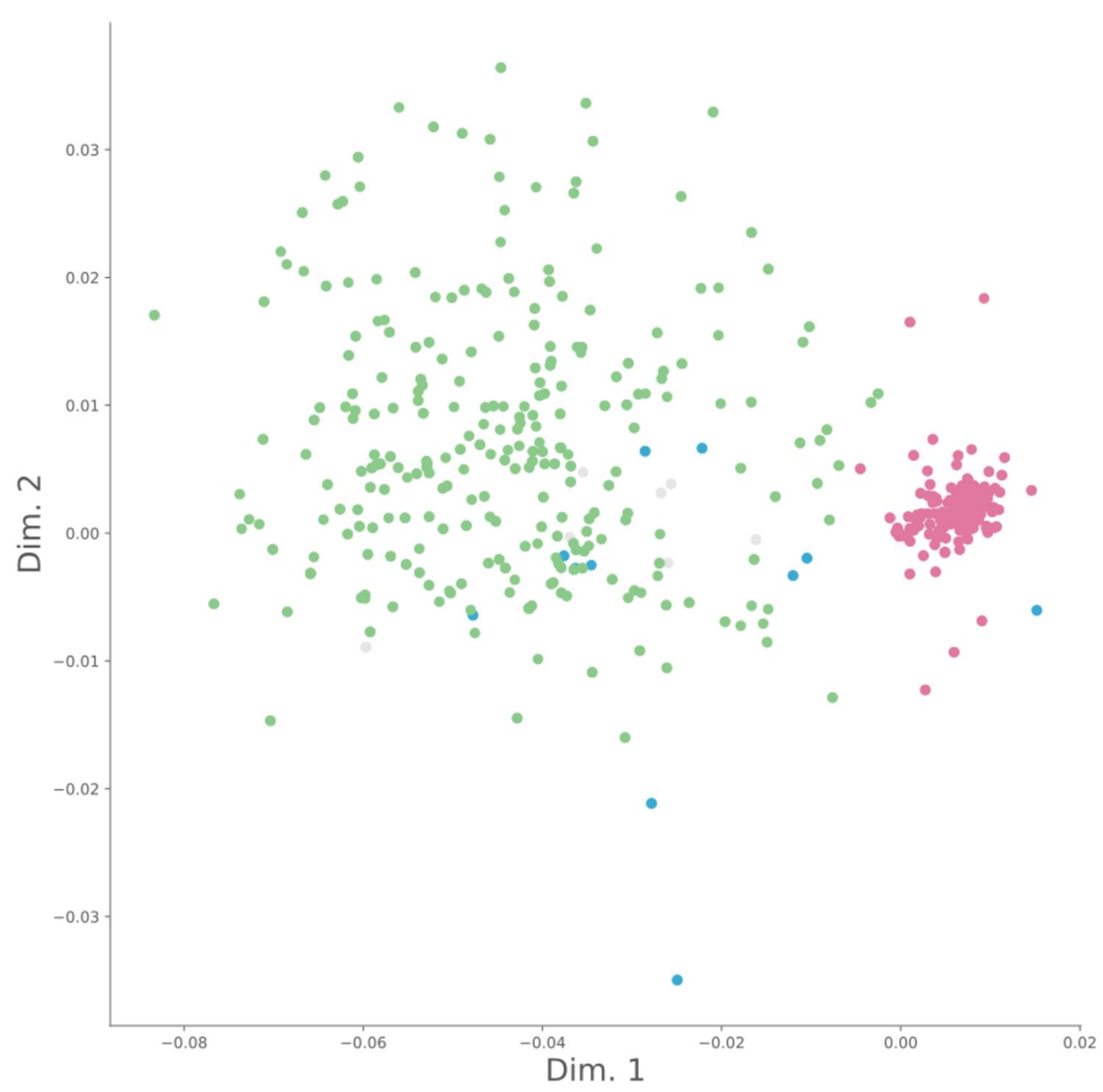

Supplement: Web_Material_uhac030 [file web_material_uhac030.zip › Supplementary figure 5.pdf]

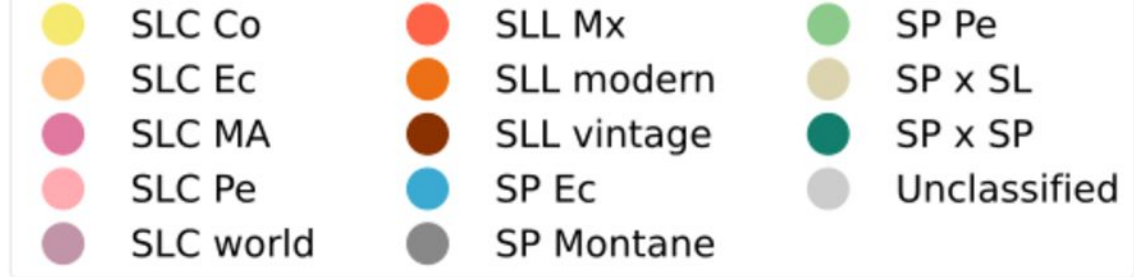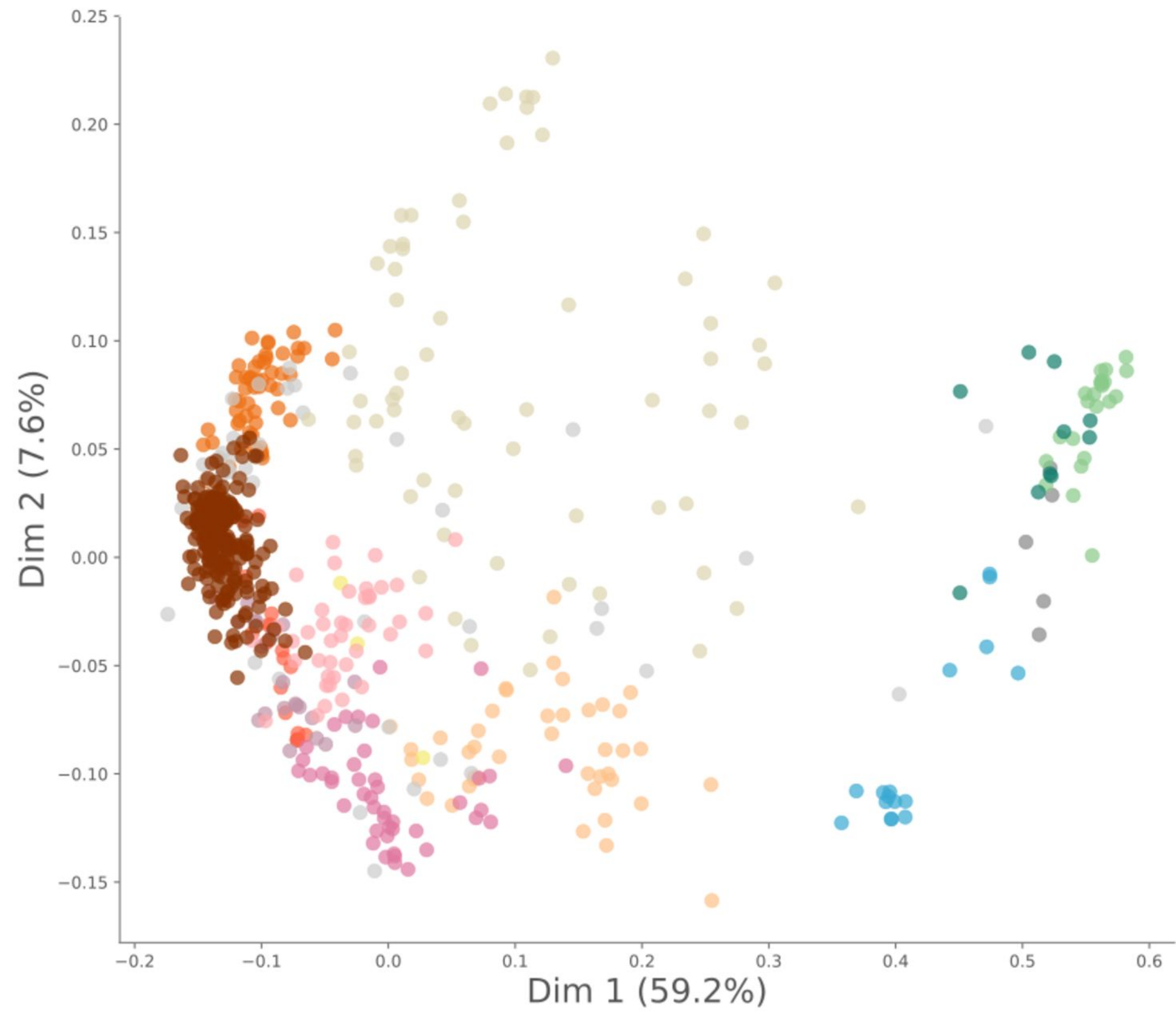

Supplement: Web_Material_uhac030 [file web_material_uhac030.zip › Supplementary figure 6.pdf]

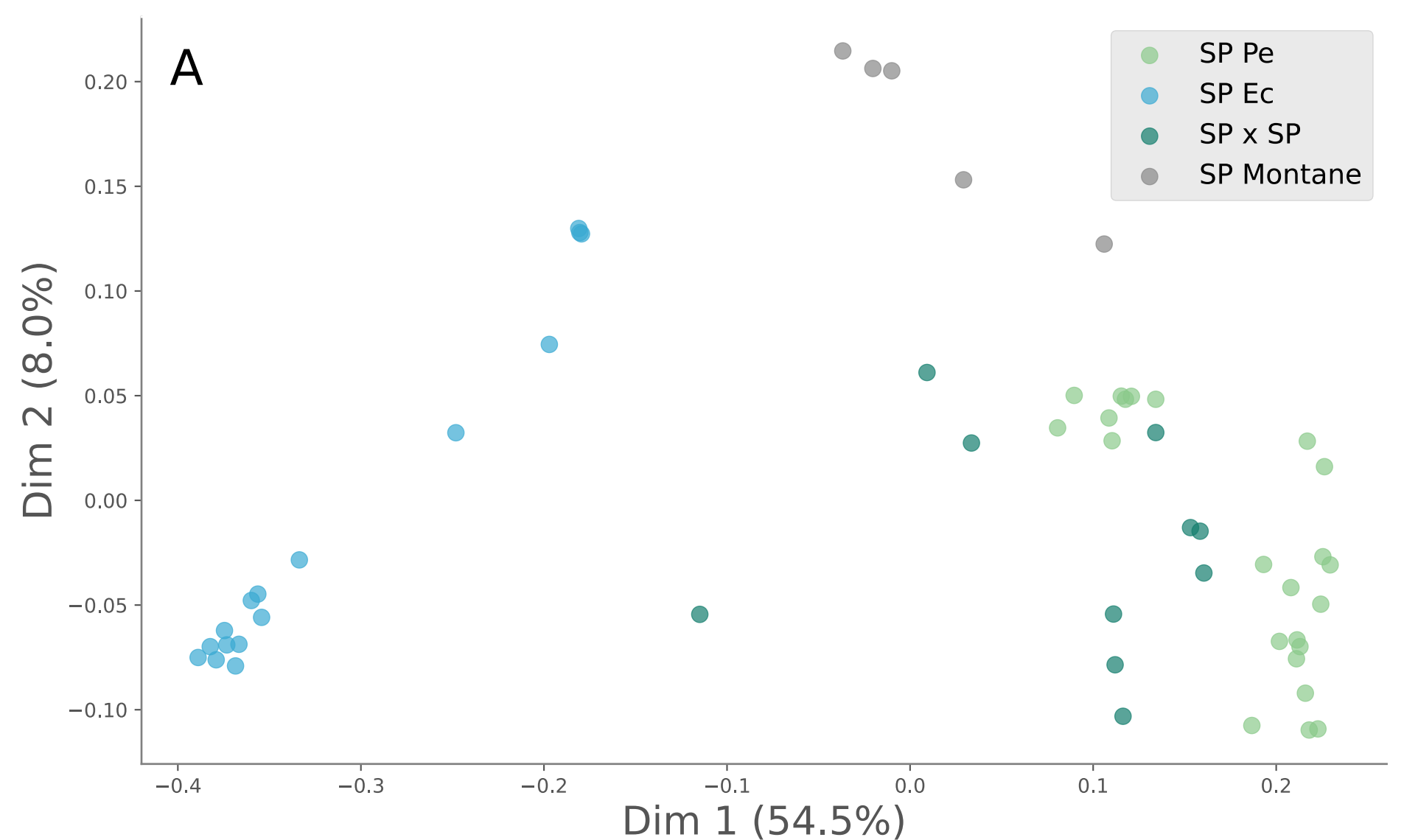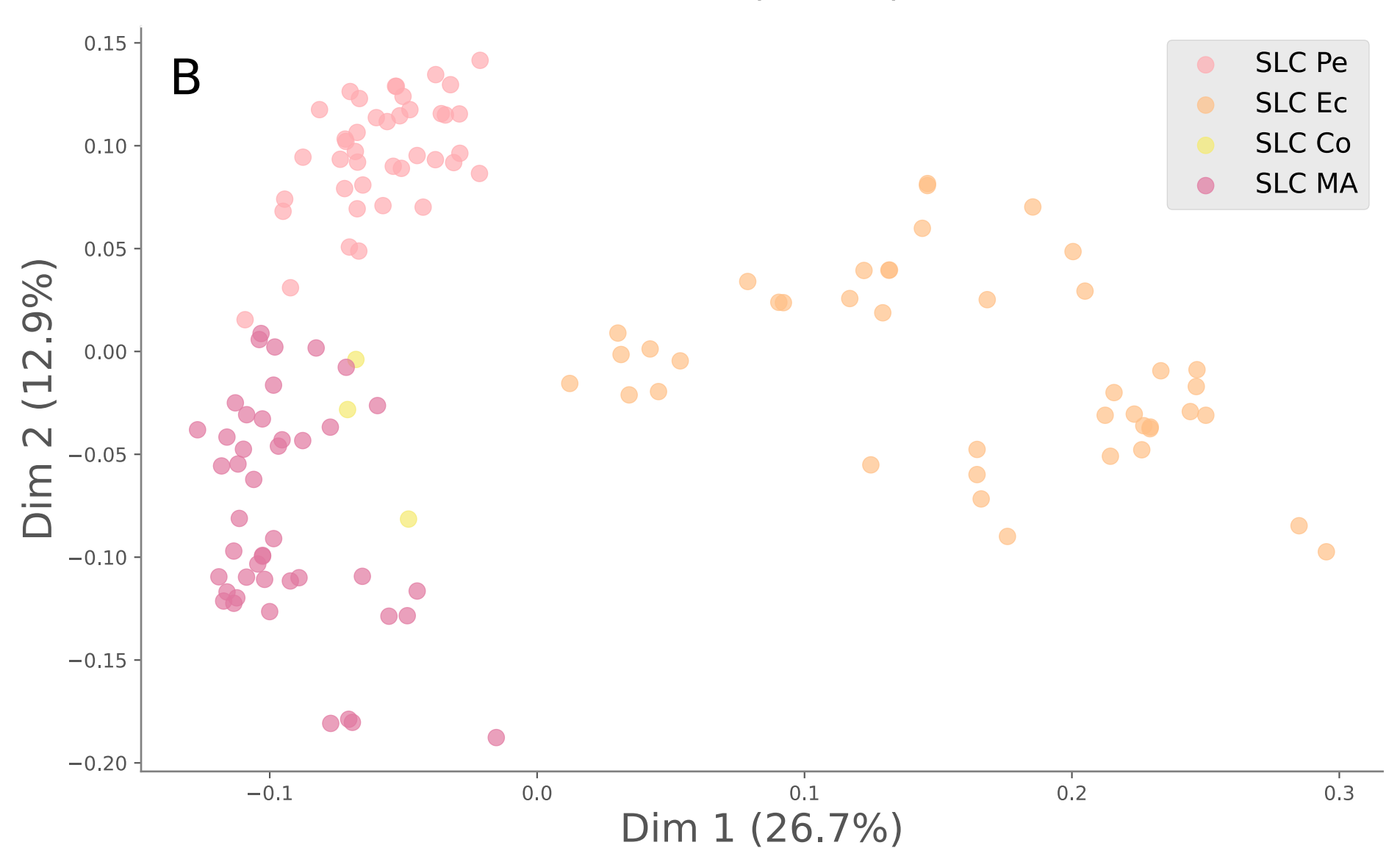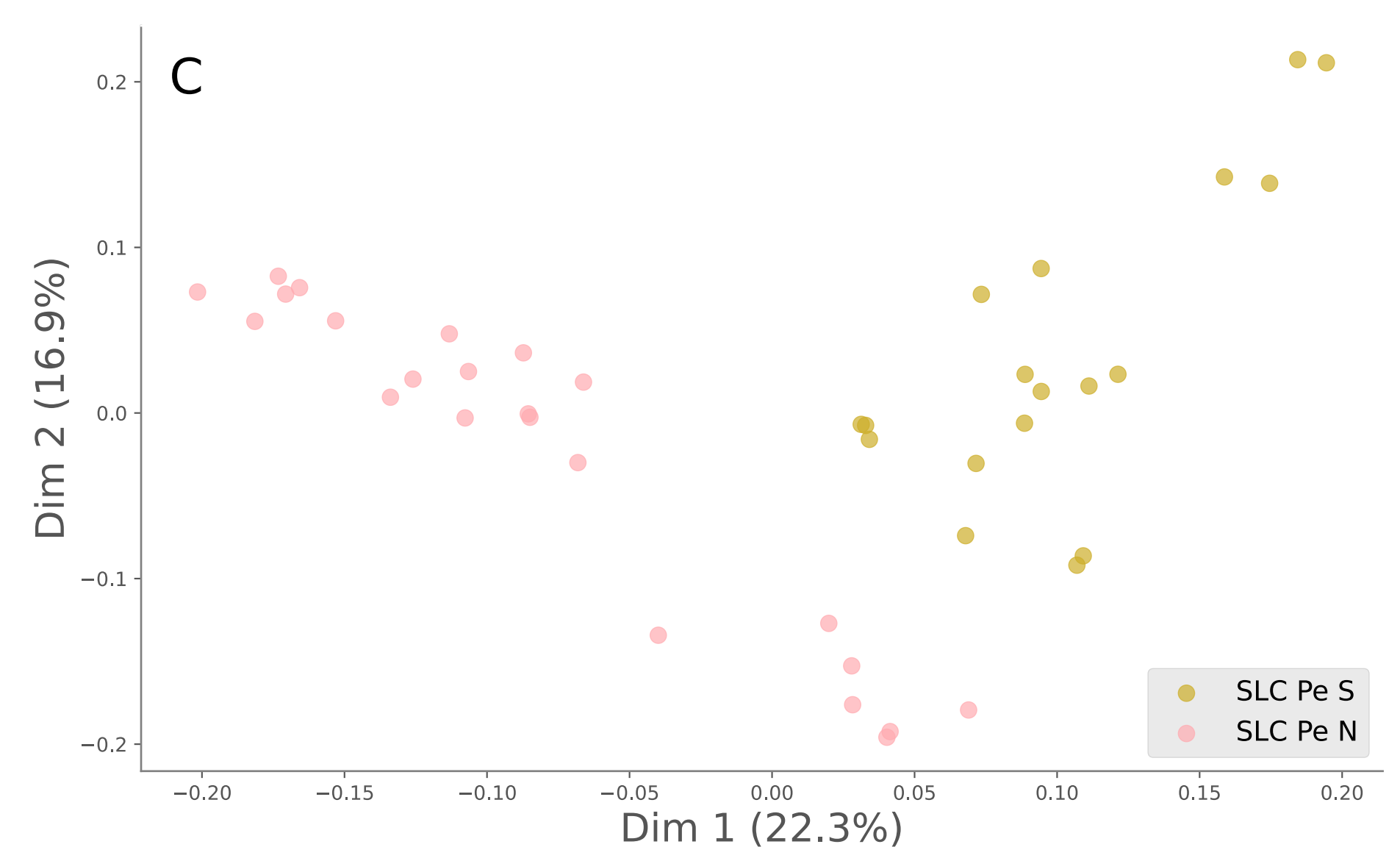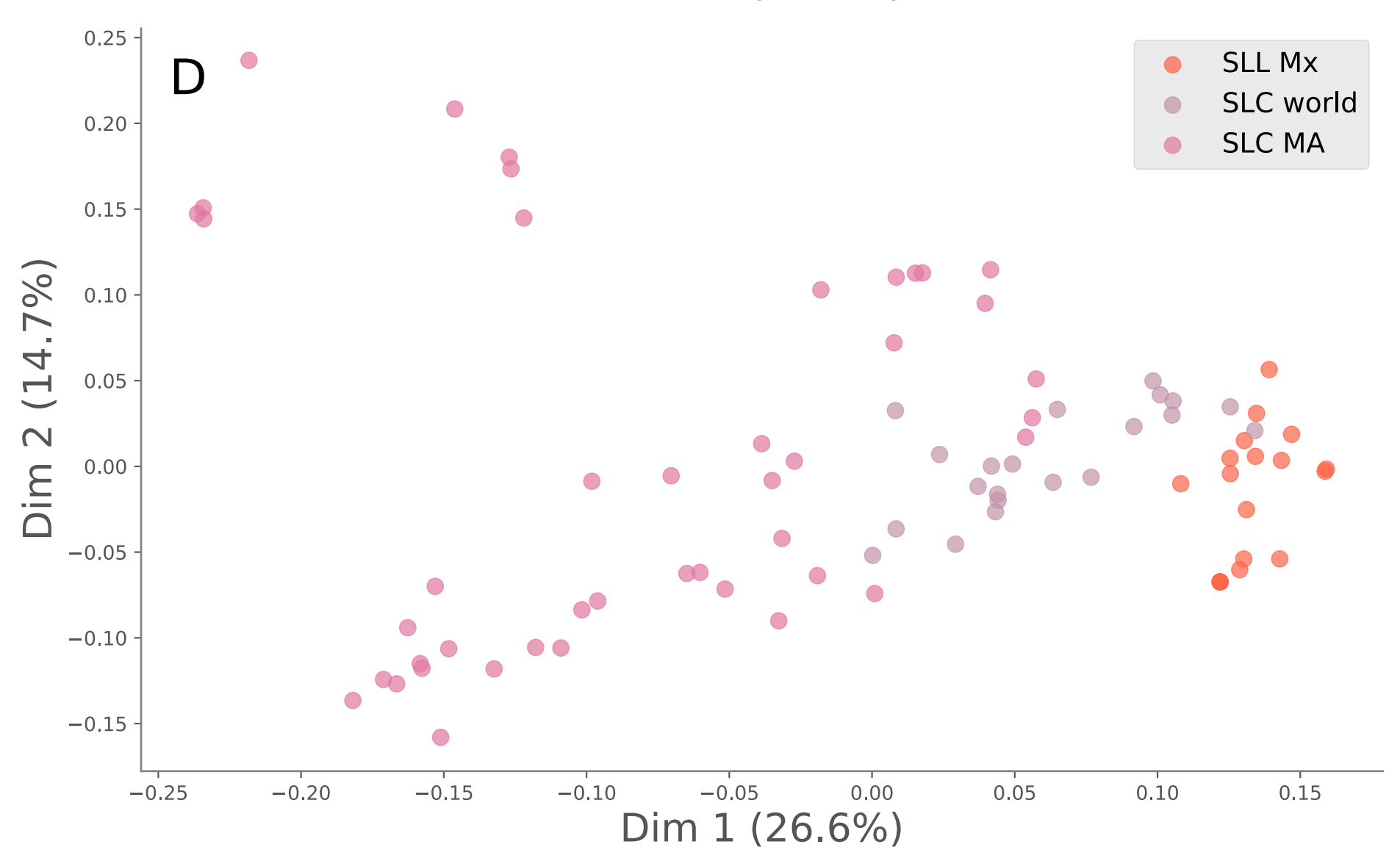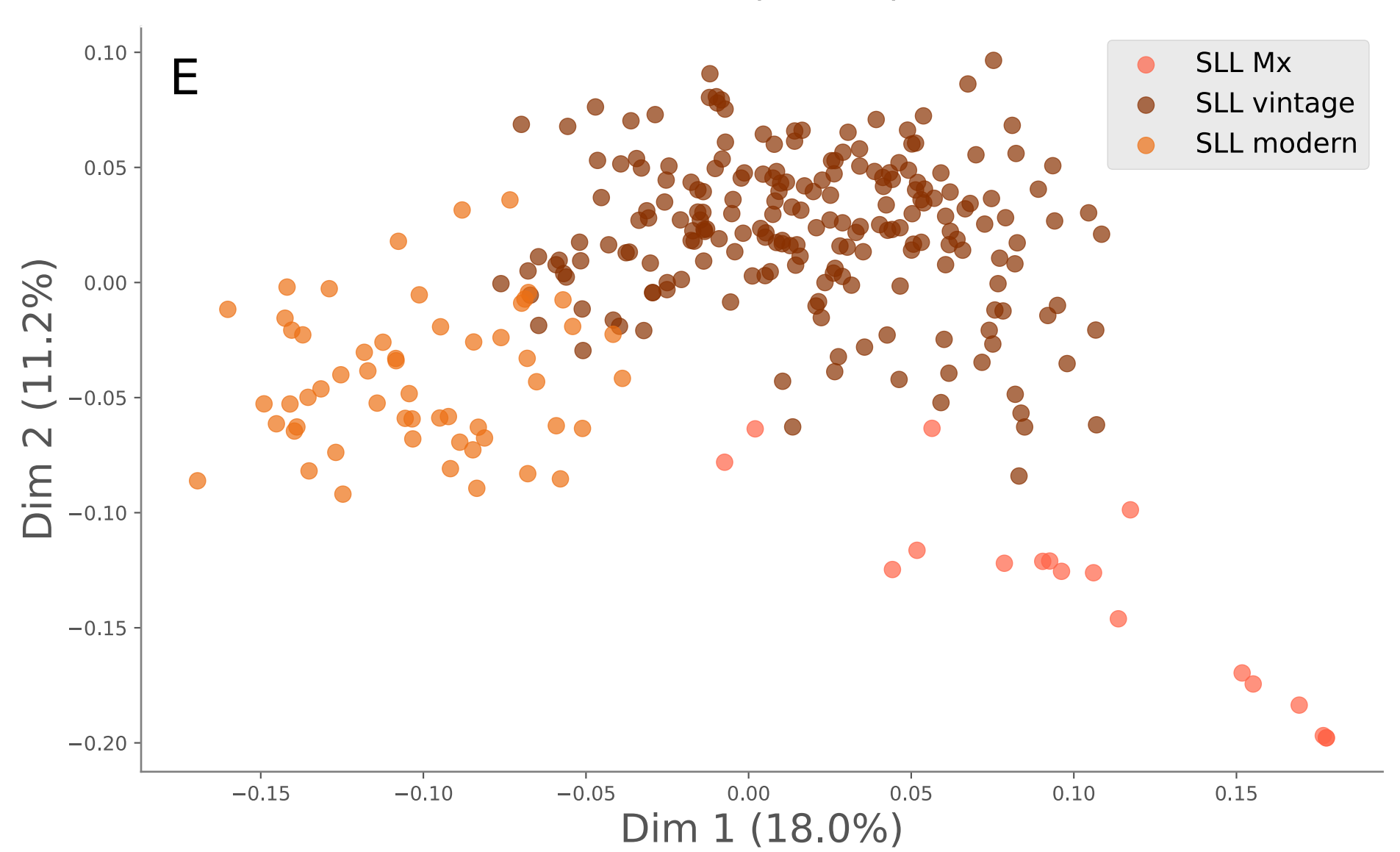

Supplement: Web_Material_uhac030 [file web_material_uhac030.zip › Supplementary figure 7.pdf]

Current study classification

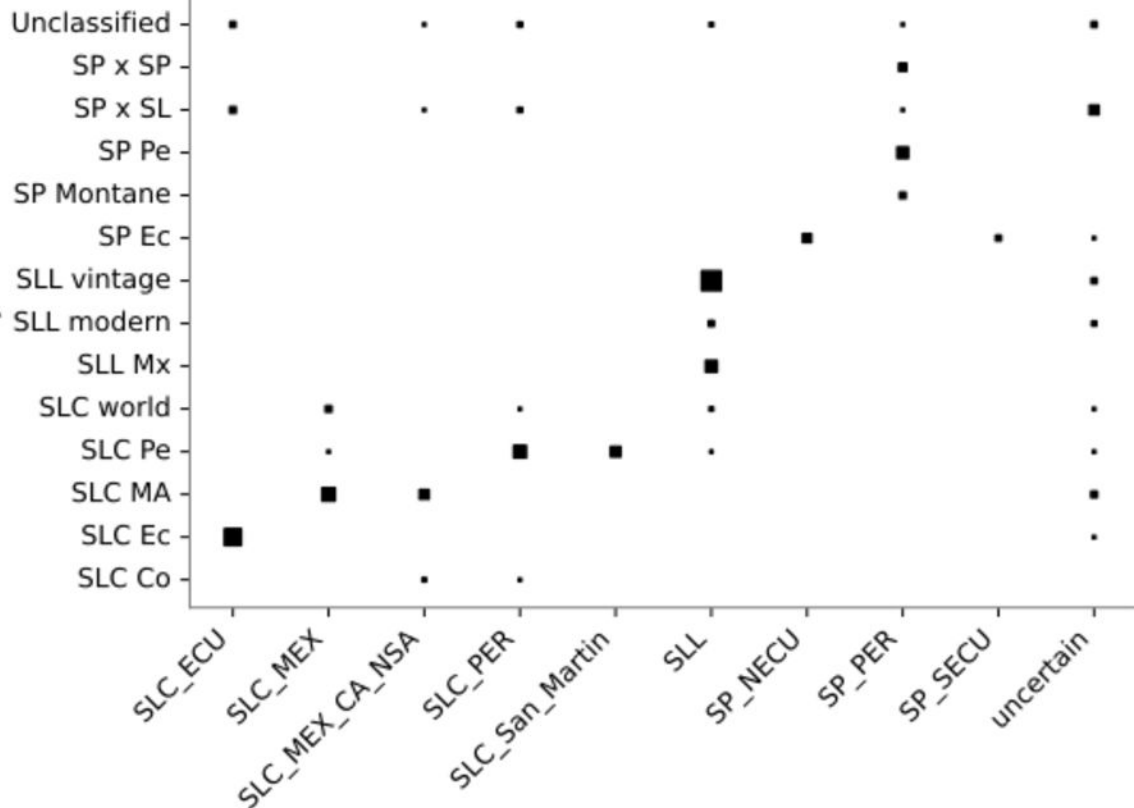

Razifard et al. classification

Supplement: Web_Material_uhac030 [file web_material_uhac030.zip › Supplementary figure 8.pdf]

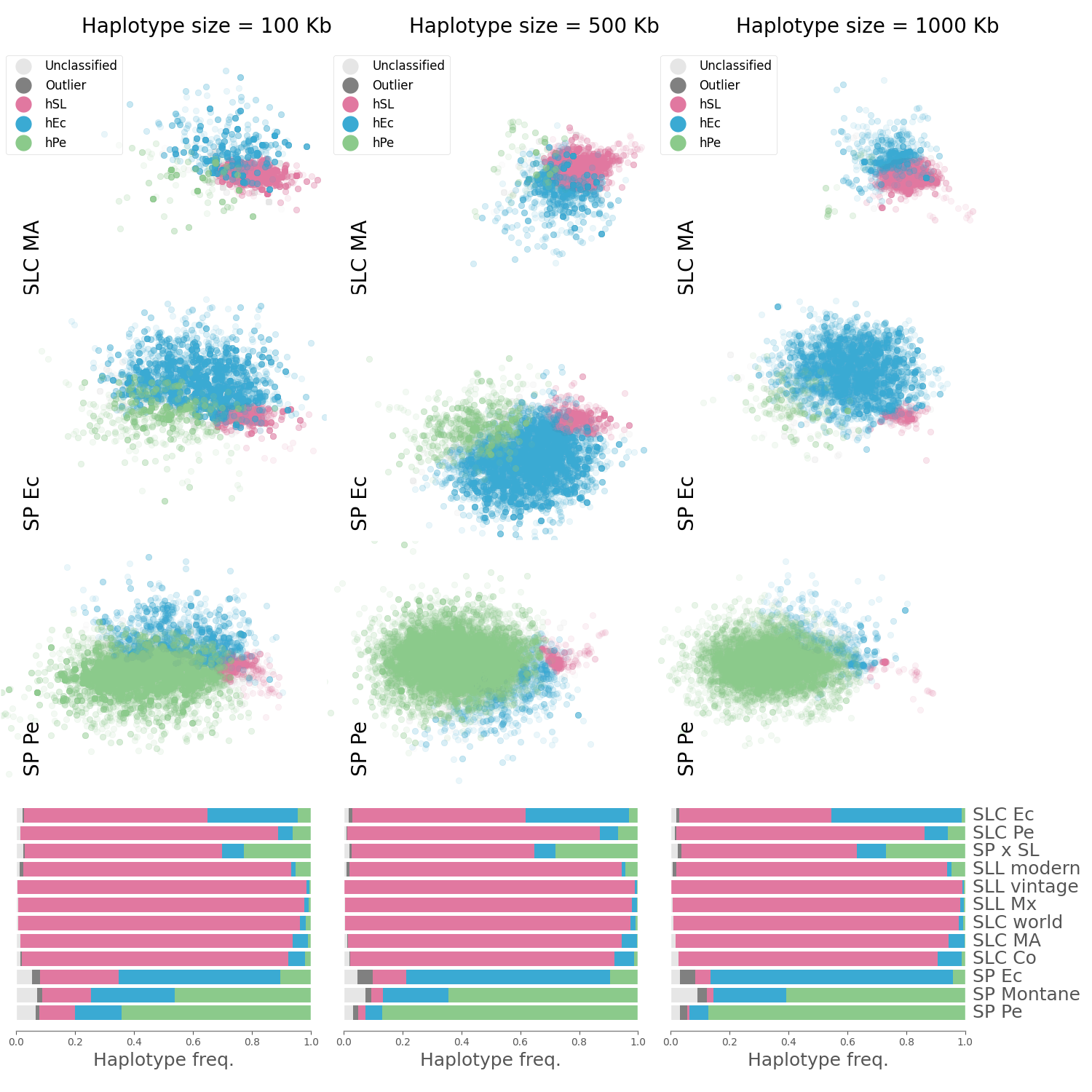

Supplement: Web_Material_uhac030 [file web_material_uhac030.zip › Supplementary figure 9.png]
